# Introns increase gene expression in *Caenorhabditis elegans* by a mechanism that must be at least partly different than in plants

**DOI:** 10.1038/s41598-025-99739-6

**Published:** 2025-05-07

**Authors:** Alan B. Rose, Aaron Baer, Isaac Shaker, J. Grey Monroe, Ian Korf, Lesilee S. Rose

**Affiliations:** 1 Department of Molecular and Cellular Biology, University of California, Davis, 95616 USA; 2 Department of Plant Sciences, University of California, Davis, 95616 USA; 3 Genome Center, University of California, Davis, 95616 USA

**Keywords:** Introns, Gene expression, Nematode, mRNA accumulation, Intron-mediated enhancement, Gene expression, Gene regulation

## Abstract

The wide diversity of organisms in which introns stimulate gene expression suggests that this is an ancient phenomenon. However, the mechanisms through which introns boost expression remain poorly understood, and the degree the which the action of introns is evolutionarily conserved is unknown. Here we compared the effect on expression of introns at different positions and tested ten different introns at the same location in a reporter gene in single-copy transgenic nematodes. The introns boosted expression most when near the start of the gene, as previously observed in several organisms. All ten introns tested at the same position increased mRNA accumulation 10- to 17-fold, in contrast to plants where introns vary widely in their effect on expression and relatively few increase mRNA levels 10-fold or more. These results suggest that some aspects of the mechanisms through which introns boost expression are fundamentally different in nematodes and plants.

**Supplementary Information:**

The online version contains supplementary material available at 10.1038/s41598-025-99739-6.

## Introduction

Within 2 years of the discovery that protein-coding sequences in eukaryotic genes can be interrupted by intervening sequences (introns)^1^, it was found that introns can increase the expression of the genes in which they are located^2^. While some introns are known to contain enhancer elements^3^, introns can also have a more general effect on expression with properties that differ from those of enhancers. For many genes, expression declines, in some cases to undetectable levels, if the introns they normally contain are deleted, while the expression of a naturally intronless gene often goes up substantially if an intron from another gene is inserted^4^. Such findings have been reported in a diverse range of organisms^5–10^, suggesting that the way in which introns boost expression is widely conserved. However, this phenomenon has been explored in just a few organisms and is well understood in none. Measuring expression solely as the enzymatic activity or fluorescent protein produced from a reporter gene is common but does not reveal which of the many aspects of gene expression is being influenced by an intron. Therefore, it remains unclear if the actual mechanism through which introns boost expression is the same in all organisms or varies from group to group. Even within a single organism it is risky to assume that all introns affect expression in the same way, or that any intron has only one effect on expression.

The known interconnections between many of the various steps of transcription, mRNA processing, export and translation provides multiple opportunities for synergistic effects that increase the overall efficiency of gene expression^11–16^. In mammalian cells, triose phosphate isomerase intron 6 located near the 5’ end of a luciferase reporter gene increases mRNA accumulation roughly 13-fold, while the same intron near the 3’ end of the gene has a 2-fold effect^17^. The increase in mRNA abundance is likely caused by an increase in transcription^18^, as a 5’ splice site enhances the recruitment of transcription initiation factors^19,20^ and splicing factors stimulate transcriptional elongation^21–23^. Introns can also cause a 2- to 4-fold increase in the amount of protein produced per unit of mRNA^17,24^.

Intron effects on mRNA half-life and nuclear export have been observed but may not be universal. While some introns increase mRNA stability^25^, and correlations have been observed between the number of introns in genes and the half-lives of the mRNA they produce^26–28^, others have found no effect of an intron on mRNA stability^17,24^. Similarly, some of the proteins in the exon junction complex that gets deposited on mRNA during splicing interact with the nuclear export machinery, increasing export of spliced mRNA to the cytoplasm^29,30^. However, other studies have found that the presence of an intron in a gene has at most a modest effect on the subcellular localization of the mRNA produced^17,24^.

A systematic analysis of the effect of introns on gene expression in plants was first performed by Callis et al. using the *Adh1* gene in maize cells^5^. These authors showed that an intron must be located in transcribed sequences to boost expression, that the intron has the greatest effect when near the 5’ end of the transcript, and that the increase in expression is present at the level of mRNA accumulation. Subsequent research confirmed these findings in Arabidopsis^31,32^. In addition, efficiently spliced introns vary widely in their effect on expression, with many having no effect^33,34^, and the sequences that stimulate mRNA accumulation are distributed throughout a stimulatory intron^34^. These observations are not consistent with the idea that the increase in expression caused by plant introns could be due to the act of splicing or involves the conserved sequences at each end of most introns.

The need for the intron to be near the promoter to affect expression led to the discovery that promoter-proximal introns in plants are compositionally distinct from other introns^34^. The location-dependent difference between introns was detected using the frequency distribution of all possible k-mers (usually pentamers or hexamers) in the genomic population of introns separated into two groups based on whether the start of the intron is less or greater than a threshold distance from the start of transcription of that gene. The IMEter algorithm generates a numerical score that describes the degree to which an individual intron is more similar to the k-mer profile of promoter-proximal introns than that of distal introns. The strong correlation between the IMEter score of an intron and its ability to increase mRNA accumulation allows the stimulating ability of an intron to be predicted from its sequence^34^. Roughly 5–10% of introns in a variety of plants have IMEter scores high enough to indicate that they are likely to have a significant effect on expression^35^. A motif that is over-represented in Arabidopsis introns with high IMEter scores can convert, in a dose-dependent manner, a non-stimulatory intron into one that strongly boosts expression with the same positional requirements as natural stimulating introns^36,37^. The same motif can increase mRNA accumulation when inserted into exonic sequences of an intronless construct^37^, demonstrating that splicing is not necessary for this motif to boost expression. Remarkably, stimulatory introns in Arabidopsis can boost the expression of a *TRP1* gene fusion containing a 303 nt promoter deletion that removes most of the 5’-UTR and all known transcription start sites^32^. Transcription in those constructs initiates in normally untranscribed intergenic sequences the same distance upstream of the intron as when the promoter is intact.

The surprising observation that introns can appear more important than the promoter in controlling the expression of a gene had previously been made in mammals and nematodes^38,39^. In *C. elegans*, an analysis of the non-coding parts of the *unc-54* gene showed that deletion of the introns but leaving the promoter and coding sequences intact nearly eliminates the ability of this gene to rescue an *unc-54* mutation in transgenic worms^38^. In contrast, a construct with the promoter deleted but introns still present is surprisingly functional, with transcription starting in the plasmid sequences that the deletion of the promoter brought next to the *unc-54* coding sequences. The discovery that bacterial sequences can serve as transcription initiation sites in worms is consistent with the finding that each of the three 200 bp random sequences tested can function as a promoter in *C. elegans*^40^.

A practical response to the finding that introns affect expression was the development of intron-containing derivatives of the *lacZ* and *GFP* reporter genes that are still widely used in *C. elegans*. One version of *lacZ* contained 12 small (51 nt) synthetic introns, as this was reported to yield higher expression than versions with 1, 2, or 4 introns (https://www.addgene.org/kits/firelab/#protocols-and-resources). More recently, introns have been inserted into many reporter genes for use in worms^41^. Introns can also help prevent gene silencing in germline cells of *C. elegans*, where obtaining consistent transgene expression is challenging^42^. A single intron is insufficient to support germline expression from extrachromosomal arrays. However, constructs containing two introns are expressed in the germline if the first intron is 274 bp or less from the start of transcription^42^.

In contrast, Crane et al.^43^ found that in *C. elegans* somatic cells a single intron, either synthetic or natural, increases expression as much as does two or three introns if the lone intron is located near the 5’ end of the gene. The same intron near the 3’ end has no effect. The two synthetic and one natural intron they tested at the same 5’ location cause equivalent increases in expression. The effect of introns varies slightly with different coding sequences and promoters of different strength. The number of introns tested in that study was too small to determine if there are significant differences between natural introns in their ability to boost expression in nematodes as there are in plants. Also, because the effect of introns was measured only as the level of fluorescence in whole transgenic worms containing *GFP* reporter constructs, it remains unclear which of the many steps that constitute gene expression is most affected by introns in nematodes.

Here we report a systematic analysis of the effect of introns on gene expression in transgenic nematodes by inserting the same introns into different locations in a reporter gene, by testing ten different introns at the same position, and by measuring steady state mRNA levels. Introns had the largest effect on expression when placed near the 5’ end of the gene. Introns near the 3’ end had much smaller effects, as did additional introns added to a construct already containing an intron. All ten of the introns tested at the same location boosted mRNA accumulation to a very similar and high degree. This suggests that despite some similarities with plants, key aspects of the underlying mechanism are different in worms and may be related to the mechanism of splicing rather than to specific sequences within introns.

## Results

### Testing intron location and number

**Fig. 1:**
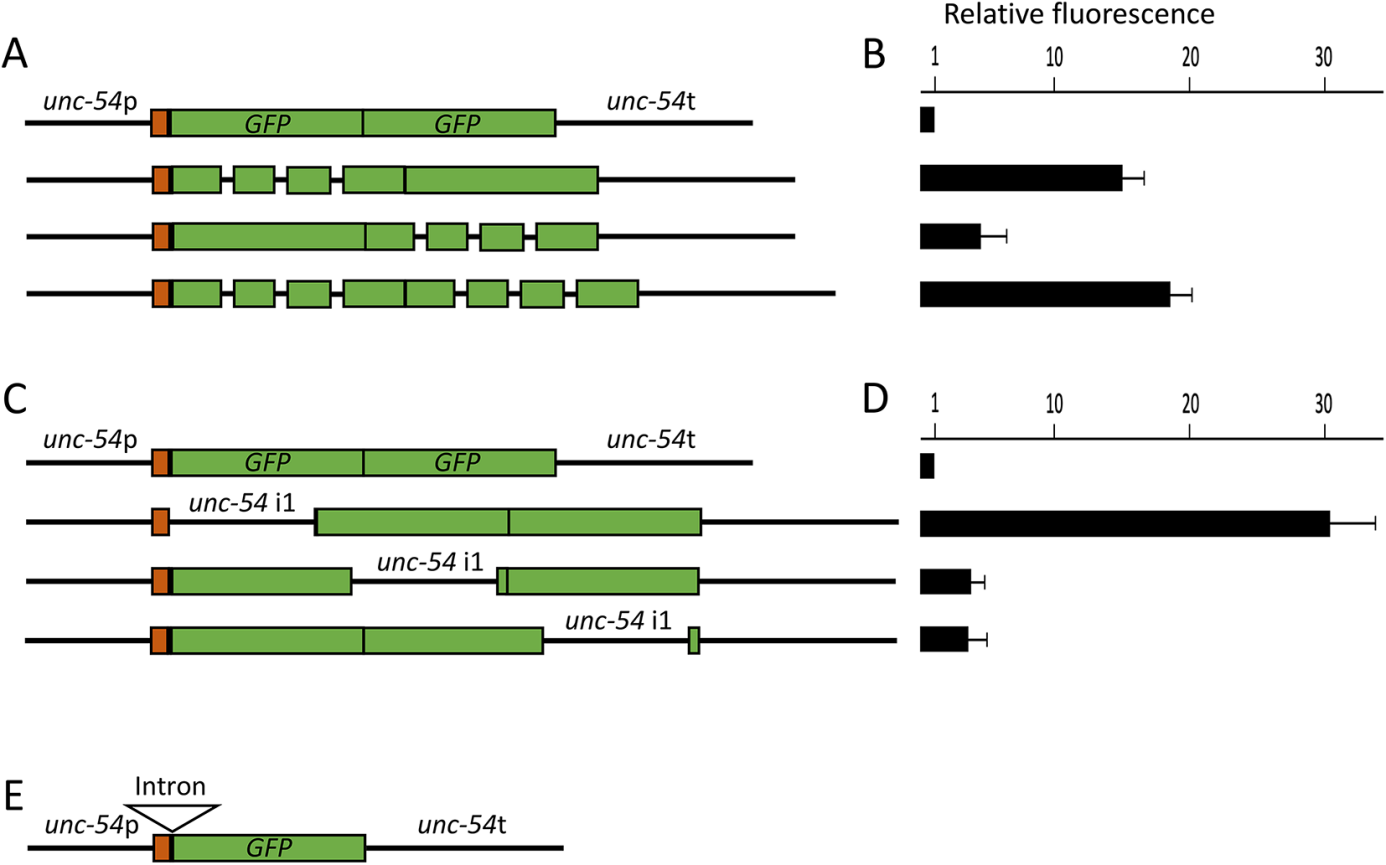
The effect on expression of varying intron location. **(a)** Diagram of constructs containing small synthetic introns. The rectangles indicate protein coding sequences, with those derived from *unc-54* in red and *GFP* in green. The lines represent non-coding sequences (*unc-54* promoter, introns, and *unc-54* terminator). **(b)** Microtiter plate readings of mean fluorescence in single-copy transgenic lines containing the constructs shown in **a**. **(c)** Diagrams of constructs containing *unc-54* intron 1 in different locations. **(d)** Microtiter plate readings of mean fluorescence in single-copy transgenic lines containing the constructs shown in **c**. In **b** and **d**, the error bars indicate standard deviations (n.s, not significant, *p* > 0.05; ** *p* < 0.01; *** *p* < 0.0001; see Supplementary Table S1 for specific P values). **(e)** Diagram of construct for comparing the effect of different introns at the same location.

The version of *GFP* most widely used in the *C. elegans* community contains three 51 nt synthetic introns in *GFP* coding sequences. To determine the relative effects on expression of different numbers of introns and intron location, an intronless but otherwise identical derivative of this *GFP* was synthesized. Different combinations of the intron-containing and intronless *GFP* genes were used to create a series of *unc-54:GFP: GFP* fusions in which the first, the second, neither or both copies of *GFP* contained three synthetic introns (Fig. 1a). The *unc-54* gene encodes a muscle myosin heavy chain protein that is expressed in body wall muscle cells and other minor muscle groups^38^. Transgenic worms containing these constructs were created by Mos1-mediated single copy insertion (MosSCI) and single-copy integration was verified by genomic DNA gel blot analysis. The level of expression varied between constructs (see below), but in all cases the green fluorescence was observed only in the body wall in a pattern that strongly resembled the histochemical staining of worms containing an *unc-54:lacZ* fusion^38^. Transgene expression was measured as GFP signal above the endogenous fluorescence of bulk worms using a fluorescent plate reader (Fig. 1b). L1 stage worms were chosen for these measurements because it was possible to generate reasonably synchronized populations of worms at that stage by collecting worms from recently starved plates.

Both constructs in which the first *GFP* contained introns were expressed at a significantly higher level than the constructs lacking introns in the first *GFP* (Fig. 1b, Supplementary Fig. S1 and Supplementary Table S1, P = 1.92 × 10^–10^). The presence of introns in the second *GFP* caused a slight increase in expression whether or not the first *GFP* contained introns. This suggests that introns near the 5’ end of a gene have a greater effect on expression than the same introns near the 3’ end of the gene, and indicates that intron position is more important than the number of introns in determining expression level.

To verify that the same single intron can have different effects on expression depending on its location, an additional set of *unc-54:GFP: GFP* fusions was created in which the *unc-54* intron was tested at its natural location between *unc-54* exons 1 and 2, or near the 3’ end of either the first or the second *GFP* (Fig. 1c). As was observed with the small synthetic introns, the *unc-54* intron had a much greater effect on expression when near the 5’ end than in the middle or near the 3’ end of the fusion construct (Fig. 1d and Supplementary Fig. S1). The *unc-54* intron alone had a larger effect on expression than the three synthetic introns but it was also located closer to the 5’ end of the gene than the first of the synthetic introns, with first exon lengths in those constructs of 142 nt and 337 nt respectively.

### Selecting different introns to test

**Fig. 2:**
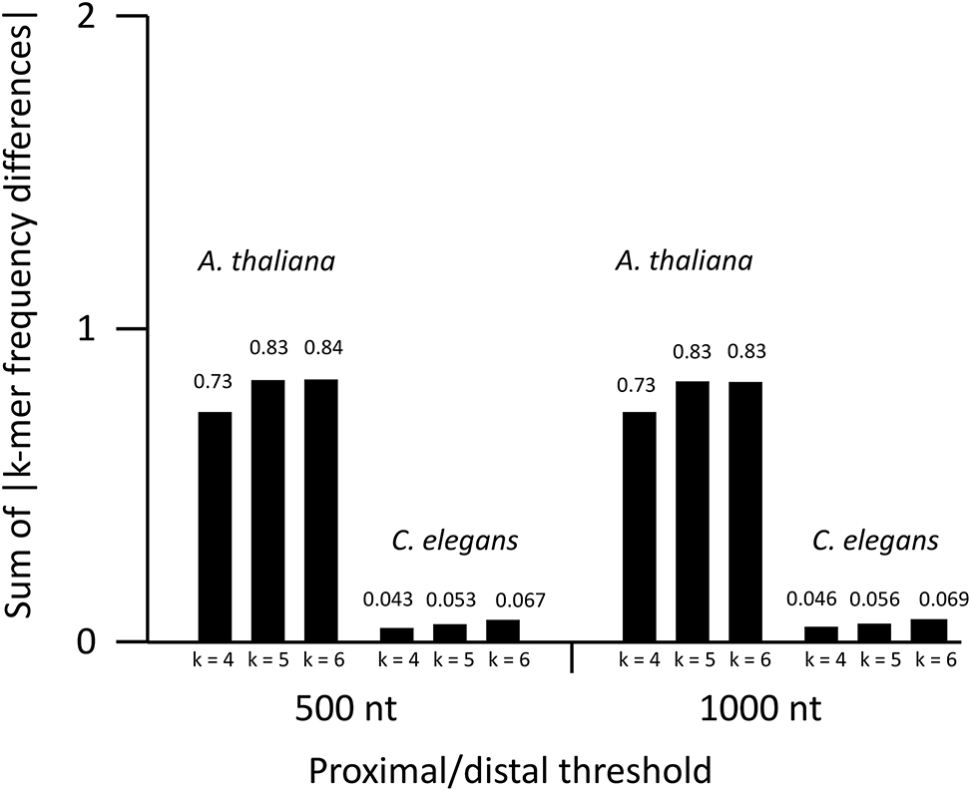
Comparing the difference in k-mer composition between promoter-proximal and distal introns in *A. thaliana* and *C. elegans*. Each bar shows the sum of the absolute values of the difference in frequency of all k-mers (size 4, 5, or 6 nt) present in the populations of introns designated promoter-proximal or distal based on a threshold distance (500 or 1000 nt) from the start of transcription to the start of the intron. Higher values indicate greater dissimilarity between promoter-proximal and distal introns.

To determine if worm introns vary widely in their ability to boost expression, as they do in plants, we attempted to create a worm IMEter for use in selecting introns with different predicted effects on expression. However, we found that the difference in k-mer composition between promoter-proximal and distal introns on which the IMEter is based is too small in *C. elegans* for the IMEter to function. One way to measure the degree to which the k-mer profiles of proximal and distal introns vary is to sum the absolute value of the differences in frequency of all k-mers in both populations of introns (see “Methods”). The exact value obtained varies depending on the choice of k-mer size and threshold distance used for separating promoter-proximal and distal introns, but the value must be between 0 (if all k-mers have identical frequencies in both populations) and 2 (if all individual k-mers are found exclusively in one population or the other). Using a k-mer size of 5 and a threshold defining promoter-proximal introns as those less than 1000 nt from the start of transcription, the sum of differences of k-mer frequencies in Arabidopsis introns is 0.83, while that of *C. elegans* introns is only 0.056 (Fig. 2), indicating that the pentamer compositions of proximal and distal introns are highly similar in worms. Therefore, we used an alternative approach to select worm introns that may have different effects on expression.

Many of the introns that are known to boost expression in plants and other organisms are found in abundantly expressed housekeeping genes, such as those encoding ribosomal proteins, actin, tubulin, histones, and translational elongation factors^44^. This led to the hypothesis that stimulatory introns typically drive the expression of that minority of genes whose products are constitutively required in large amounts in all cells, while conventional transcription factors control the expression of most genes whose products are needed in only certain tissue types, at specific times, or in response to a particular stimulus^44^. If introns have variable effects on the expression of the gene in which they are naturally found in worms, the introns from highly expressed should be more likely to stimulate expression than introns from genes expressed at a very low level. Therefore, seven introns were chosen from a variety of genes based on the following criteria. First, *C. elegans* genes were ranked based on their expression level. The most highly expressed genes had approximately 10^6^ RNAseq reads as reported on WormBase (https://wormbase.org). Genes were also selected that had approximately 10^5^, 10^4^, or 10^3^ RNAseq reads, forming four expression categories referred to as high, medium, low, and very low. Second, within those categories, genes were selected that have been sufficiently studied to be named, are not in an operon, and do not have multiple alternative splice isoforms. Finally, the introns chosen to test from these genes are between 300 and 600 nt in length and are located less than 500 nt from the start of transcription. An additional intron tested was *smu-1* intron 3, which contains poly A/T-rich clusters (PATCs) and was previously shown to prevent the silencing of genes in the germline^45^. The exact mechanism through which PATCs prevent silencing is unclear, and this intron was chosen to explore the possibility PATC-containing introns avoid silencing at least in part by causing an unusually large increase in the expression of the gene in which they are located. The eight new introns were compared to two of the introns tested in the *unc-54:GFP: GF*P fusions described above, namely *unc-54* intron 1 and the 5’-most of the 51 nt synthetic introns.

### Comparing the stimulating effect of different introns

**Fig. 3:**
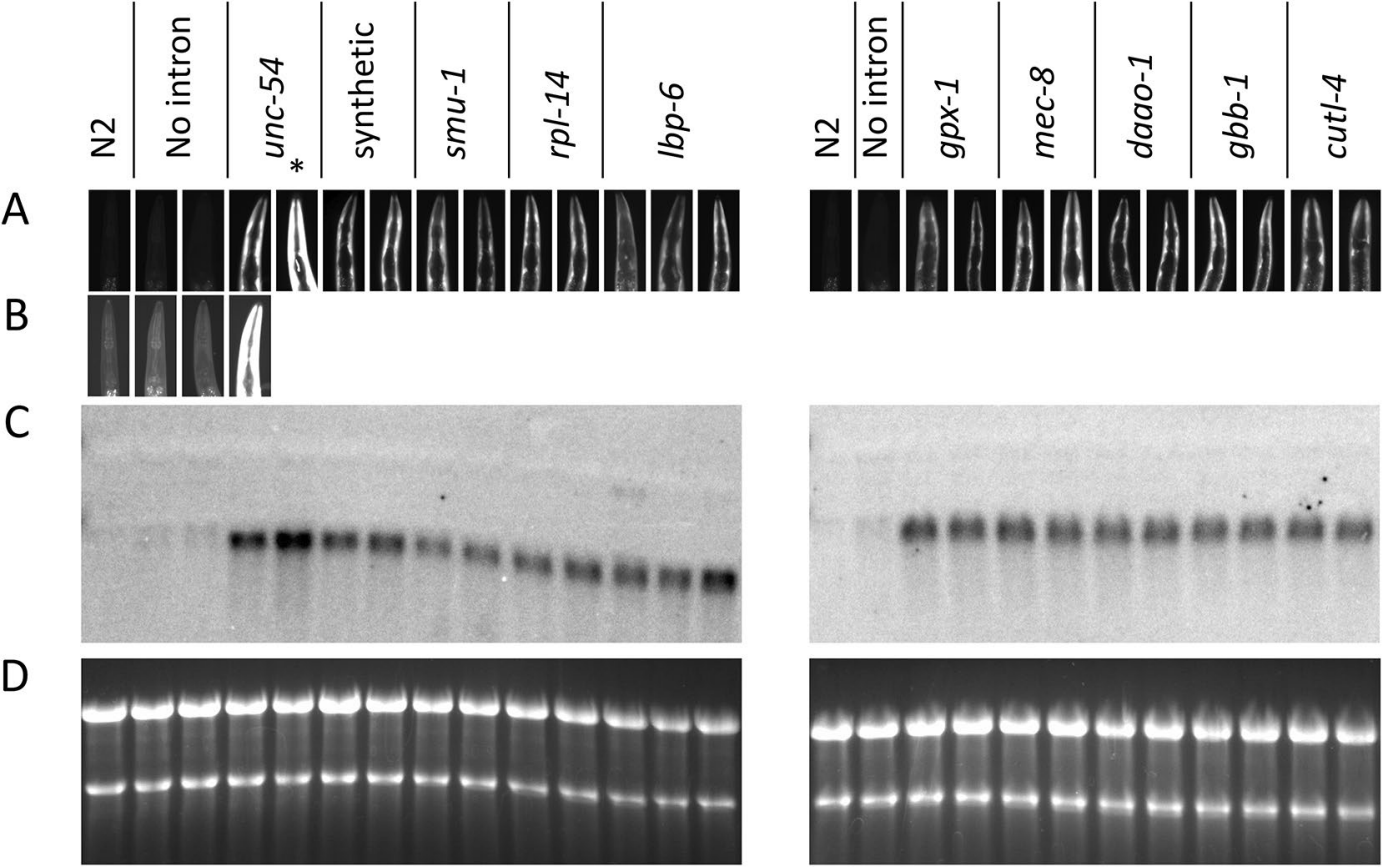
The effect of different introns on GFP fluorescence and mRNA accumulation. The lanes in all four panels are vertically aligned and show fluorescence micrographs (**a** and **b**) or RNA gel blots (**c** and **d**) using RNA extracted from the same line of untransformed worms or transgenic worms containing *unc-54::GFP* fusions with no intron or an intron from the indicated gene. Each column under the same heading represents an independent line containing the indicated construct. All are single copy except the line marked with an asterisk, which contains two copies of the transgene. **(a)** Pharynx of worms. **(b)** As in **a** but at a brighter exposure to reveal the faint fluorescence in the untransformed and intronless lines. **(c)** RNA gel blot probed with *GFP*. **(d)** The ethidium bromide-stained gels used in **c** to show that each lane contains a similar amount of undegraded RNA. The images in **c** and **d** have been cropped. Original blots and gels are presented in Supplementary Fig. S5.

**Fig. 4:**
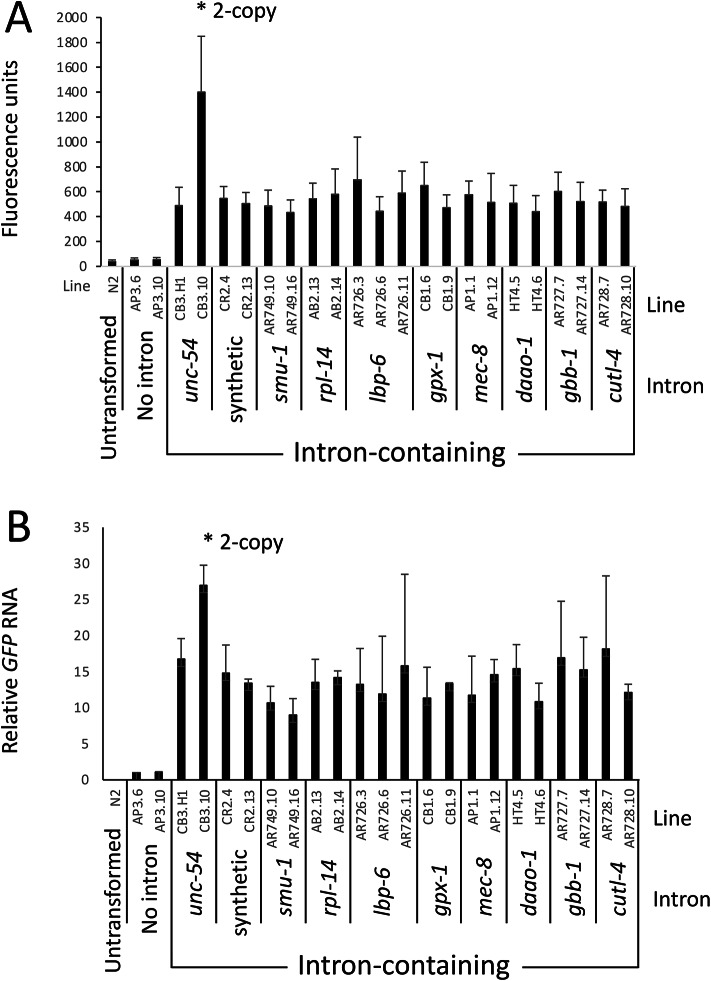
Quantification of GFP fluorescence and mRNA accumulation in lines containing different introns. **(a)** Mean fluorescence readings are shown to illustrate the signal relative the endogenous autofluorescence of untransformed worms as well as the intronless control. The fold increase in fluorescence caused by each intron is presented in Supplementary Table S2 and Supplementary Fig. S1. **(b)** RNA gel blot quantification showing the mean fold increase in GFP mRNA relative to the intronless control. Line CB3.10 (marked with an asterisk) contains two copies of the transgene: all other transgenic lines are single-copy. Error bars indicate standard deviation.

The ten introns were individually inserted into an *unc-54:GFP* reporter gene at the same location as the natural *unc-54* intron 1 (Fig. 1e). Fluorescence was measured in single-copy transgenic worms by photographing individual worms on a fluorescence microscope and measuring fluorescence in the body wall muscles where *unc-54* is expressed and endogenous autofluorescence is low, specifically in the head of the worm adjacent to the pharynx (Fig. 3a). The L3 stage was chosen for these experiments because the GFP signal is higher than in L1 worms. The intronless control was expressed at a very low level, generating fluorescence that was slightly but significantly (*p* < 1 × 10^-4^) above the endogenous fluorescence in untransformed worms (Figs. 3a and b and 4a, and Supplementary Fig. S1). The effect of each intron on expression is reported as the fold increase in pharynx fluorescence relative to that in the intronless control, although the accuracy of this measure is limited by the very low expression of the intronless control.

All ten introns boosted fluorescence 30- to 40-fold relative to the intronless control (*p* < 1 × 10^–10^), and all independent single-copy lines containing the same construct gave very similar results (Figs. 3 and 4a; Table 1 and Supplementary Table S2). Only one single-copy line containing the construct with the *unc-54* intron was obtained but the values obtained from this line are supported by a line containing a two-copy insert, which gave roughly twice the fluorescence as the single-copy line. There was considerable variation in fluorescence measurements between individual worms of the same genotype measured at the same time. This variation appeared to be mostly due to slight differences in the ages of the worms examined. Pharynx length, which increases at a steady rate as worms grow^46^, correlated with fluorescence (Supplementary Fig. S2), indicating that GFP accumulates as the worms age. Despite the similar mean expression levels and large overlapping standard deviations for the data sets obtained from worms containing the same construct, the combined data for some of the introns were statistically different (*p* > 0.05). However, an analysis of the source of the variation indicated that differences between lines containing the same construct, observations of the same line taken on different days, and the individual taking the measurements all contributed more to the variation than did the identity of the intron (Supplementary Fig. S1). Indeed, we found no introns for which all independent lines containing that construct were significantly different than the individual lines containing another intron. In contrast, the GFP fluorescence of all intron-containing lines were significantly higher than the intronless control (*p* < 2 × 10^–10^). Thus, we conclude that all ten introns cause a similarly large increase in GFP fluorescence, strongly suggesting that all introns in *C. elegans* boost expression equally well.

**Table 1:** The introns tested and their effect on expression.

Gene source of intron			Intron in native gene			Effect on expression	
Name	Encoded protein	Expression level	Intron size (nt)	Intron #	Distance from ATG (nt)	Fluorescence	mRNA
*unc-54*	Myosin heavy chain	High	562	1	63	**33.6 +/- 11.2**	**16.7 +/- 2.9**
synthetic			51			**36.5 +/- 7.1**	**14.2 +/- 2.9**
*smu-1*	Spliceosomal subunit	Medium	305	3	380	**31.1 +/- 8.7**	**9.8 +/- 2.1**
*rpl-14*	Ribosomal protein	High	346	1	105	**39.1 +/- 12.7**	**13.8 +/- 2.3**
*lbp-6*	Lipid binding protein	High	554	1	207	**38.9 +/- 18.1**	**13.8 +/- 9.0**
*gpx-1*	Glutathione peroxidase	Medium	444	2	211	**38.5 +/- 12.8**	**12.4 +/- 2.7**
*mec-8*	mRNA binding protein	Medium	528	1	283	**38.0 +/- 13.5**	**13.2 +/- 3.7**
*daao-1*	D-amino acid oxidase	Low	406	2	274	**32.8 +/- 10.6**	**13.1 +/- 3.6**
*gbb-1*	GABA B receptor	Low	524	1	228	**39.6 +/- 12.0**	**16.1+/- 5.3**
*cutl-4*	Cuticulin-like	Very low	354	1	88	**34.6 +/- 8.9**	**15.1 +/- 6.9**

Significant values are in [bold].

The effect on expression is the fold increase in *GFP* fluorescence or mRNA in lines containing a transgene with that intron relative to the intronless control, mean +/- standard deviation.

The synthetic intron and *unc-54* intron 1 both increased fluorescence to a very similar degree (36.5 +/- 7.1-fold and 33.6 +/- 11.2-fold respectively, Table 1). This indicates that the higher expression of the *unc-54:GFP: GFP* construct containing a single *unc-54* intron at its normal position compared to that of the three synthetic introns in the first GFP (Fig. 1) was due entirely to the location of the introns, rather than any difference in their stimulating ability.

### Introns stimulate mRNA accumulation

To determine the level at which expression is primarily affected, total RNA was isolated from partially synchronized populations of worms that were predominantly at the L3 stage. RNA gel blots probed with *GFP* revealed that all ten of the tested introns caused an increase in *GFP* mRNA of between 10- and 17-fold relative to the intronless control (Figs. 3c and 4b; Table 1). As with the fluorescence measurements, accurate estimations of the intron-caused fold increase in the level of *GFP* mRNA are hindered by the very low levels of signal in the intronless control lines. In addition, there is a faint cross-hybridizing band visible even in RNA from untransformed worms that migrates just above the *GFP* band that also interferes with quantification of *GFP* mRNA levels in the intronless control. Nevertheless, it is clear that a small amount of GFP mRNA accumulates in lines containing the intronless construct, and that all ten introns cause a similarly large increase in GFP mRNA. The mRNA from all intron-containing constructs comigrates with that seen in the intronless control, with no larger bands visible, indicating that all the introns tested are spliced with similar high efficiency. Thus, the difference in GFP mRNA accumulation accounts for most or possibly all the large difference in fluorescence seen between the intron-containing and intronless lines.

## Discussion

Here we show that introns boost expression in *C. elegans* most when the intron is closest to the 5’ end of a gene, and that the first intron inserted has the greatest influence on expression, while additional introns have effects that are much smaller and less than additive. These findings confirm and extend observations previously made in worms^43^ and other organisms. In addition, we showed that all ten introns tested at the same location caused a similar strong increase in expression, and that the overall effect of an intron on gene expression was due mostly or entirely to differences in mRNA accumulation.

The magnitude of the difference in expression of intron-containing and intronless constructs we observed was substantially larger than that reported by Crane et al.^43^ (more than 30-fold versus 1.5 to 1.8-fold at the level of fluorescence). The main cause of this difference is the very low expression of our intronless *unc-54:GFP* fusions, while the intronless *hsp-90:mCherry*, *hsp-90:GFP*, and *vit-2:mCherry* fusions used by Crane et al. were expressed at relatively high levels. One potential explanation is that one of the two *hsp-90* transcript isoforms (C47E8.5.2) reveals the presence in the *hsp-90* 5’-UTR of a 1,116 nt intron whose 3’ end is 4 nt upstream of the ATG (https://wormbase.org/*).* Because this entire intron is present in the 2 kb promoter fragment used for all *hsp-90* constructs, even those described as intronless, this 5’-UTR intron might be responsible for the relatively high expression of constructs lacking introns in the reporter genes. The introns subsequently added to the reporter gene would have a minor effect since they are not the first intron in the gene. However, there is no evidence for an intron in the 5’-UTR of *vit-2* that could explain the high expression of the intronless *vit-2:mCherry* fusion.

Alternatively, it is possible that *C. elegans* is similar to mammals where the expression of some genes such as β-globin absolutely requires the presence of an intron, while other genes such as dihydrofolate reductase are expressed at detectable levels without an intron even if their expression can be boosted by an intron^4^. Perhaps *unc-54* is an example of an intron-dependent gene while *hsp-90* and *vit-2* are intron-independent. Crane et al.^43^ also examined expression in adult worms that have had longer to accumulate GFP from a poorly expressed construct than the L3 worms used here. Other potential sources of variability can be ruled out. The synthetic intron we used was identical to their intron “ia”, the introns were similar distances from the start of transcription, the *unc-54* terminator was used in both studies, and the constructs were integrated using the same technique at the same chromosomal location.

Several observations suggest that unlike in plants where specific stimulating sequences present only within a minority of introns are responsible for increasing mRNA accumulation, in nematodes it may be a conserved intron structure (such as a splice site) or the act of splicing that boosts expression. First, all ten of the introns tested increased expression to a very similar degree and none failed to have an effect. The recent observation that virtually all of the 19,346 short random introns tested increase mRNA accumulation roughly 8.5-fold in mammalian cells^47^ suggests that introns increase expression via splicing or conserved splice site sequences in mammals as well. Second, *C. elegans* introns lack the significant k-mer compositional differences between promoter-proximal and distal introns seen in plants, even though introns boost expression most when located nearest to the 5’ end of a gene in both plants and nematodes. Third, there is very little room in the 51 nt synthetic intron for sequences that could boost expression to the same degree as introns that are more than ten times its length, such as the 562 nt *unc-54* intron 1. In Arabidopsis, the stimulating sequences within introns have additive effects^37^. This may be one reason that first introns, which are the most likely to boost expression, also tend to be longer than other introns^48^.

One possible explanation for a splicing related increase in GFP fluorescence is that intron-containing and intronless constructs might yield the same amount of primary transcript but that only spliced mRNAs are efficiently exported to the cytoplasm and translated. The exon junction complex (EJC) proteins that are deposited on the mRNA during splicing are known to promote export^49,50^. However, the RNA used in Fig. 3 was isolated from whole worms and thus would include both nuclear and cytoplasmic RNA. The observation that intron-containing constructs produced 10 to 15 times more GFP mRNA than did the intronless control rules out the possibility that the primary cause of the different expression levels was the subcellular localization of the mRNA. The EJC could have a roughly 2-fold effect on expression because the observed 30- to 40-fold increase in GFP fluorescence caused by introns was roughly twice that seen at the level of mRNA, suggesting that more protein was produced per unit of mRNA when the gene contained an intron. Similar observations have been made in plants where the effect of an intron is consistently twice as large measured at the level of enzyme activity than as mRNA accumulation, which has been attributed to increases in export and translation^31,36,37^. Still, in both worms and plants, the intron-mediated increase in expression can be largely or entirely attributed to increases in mRNA accumulation.

The effect of the introns on mRNA accumulation could either be a positive action that increases mRNA production, or the removal of a repression that inhibits expression of intronless constructs. In one example of introns actively stimulating expression, some human genes with weak promoters upstream of alternatively spliced exons have the occupancy of RNA polymerase II boosted by specific splicing factors that recruit the core transcription machinery^51^. In an example of intronless repression in the *C. elegans* germline, the removal of introns from a gene can cause it to be silenced by two distinct pathways, one of which uses argonaute proteins and small RNAs^52^. In the intron-containing versions of the same genes, intron splicing somehow marks transcripts and prevents their recognition as templates for argonaute-mediated silencing. However, the same intronless genes are not silenced in the somatic cells^52^ where the *unc-54:GFP* fusions studied here are predominantly expressed.

In summary, there are some similarities and some differences in the effect of introns in worms and plants. In both groups, the effect of the intron on expression was primarily at the level of mRNA accumulation and was larger if the intron was near the 5’ end of the gene than if the same intron was located further away from the start. In addition, the remarkable ability of introns to affect expression in the absence of a conventional promoter^32,38^ suggests a mechanism that is similar in both groups and distinct from that of enhancer elements, which cannot activate expression in the absence of a promoter and can have effects over much greater distances than introns can. However, a significant difference is that in plants, only an estimated 5–10% of introns boost mRNA accumulation tenfold or more while others have a much smaller effect or none at all^35^. In contrast, all ten of the worm introns tested here had the same strong effect on mRNA accumulation. In plants and nematodes, introns could increase steady state mRNA levels by stimulating transcription, decreasing mRNA turnover, or both.

Perhaps the underlying mechanism in both plants and worms involves introns directing transcript initiation upstream of themselves through interactions between introns and the transcription machinery, explaining the need for introns to be near the 5’ end of the gene and the surprising dispensability of promoter sequences. In plants these interactions could be mediated by proteins that bind to sequences present in variable numbers within the body of a minority of introns, while in worms they could involve the spliceosome or other proteins that recognize conserved sequences around the splice sites of all introns. The results from worms, plants, and mammals illustrate that sequences downstream of the transcription start site, especially those near the 5’ end of a gene, can have effects on expression that outweigh those of the more familiar regulatory sequences in the promoter around and upstream of the transcription start site^53^.

## Methods

### Worms

*C. elegans* strains were maintained at room temperature on MYOB plates with *E. coli* OP50 as a food source^54,55^. The wild-type strain is N2.

### Constructs

All constructs contain the same 453 nt *unc-54* (F11C3.3) promoter fragment, the entire 95 nt *unc-54* exon 1 (32 nt of 5’-UTR and 63 nt coding sequence), the first 9 nt of *unc-54* exon 2, some version of *GFP*, and the same 713 nt *unc-54* terminator fragment. The sequences of the fusions can be found in Supplementary Fig. S3. They vary in the identity of the intron present at the normal location of *unc-54* intron 1, the number of *GFP* genes, and the identity and location of the introns in *GFP*. The *unc-54* promoter fragment was amplified from genomic DNA using primers OAR130 and OAR131, the *unc-54* intron was removed by amplification with OAR130 and OAR132, and the *unc-54* terminator was amplified with primers OAR135 and OAR136 (primer sequences are found in Supplementary Table S3). A *GFP* gene containing three 51 nt synthetic introns was amplified from plasmid pPD95.75 (Addgene), and an otherwise identical intronless version was synthesized by Biomatik (Kitchener, Ontario). Derivatives of the various intron-containing and intronless *GFP*s in which the stop codon was replaced by a 12 nt region encoding a 4 amino acid linker and containing various flanking restriction sites were created by PCR to facilitate the construction of the tandem *GFP* fusions. The *unc-54* intron was inserted by Gibson assembly using the NEBuilder kit (New England Biolabs, Ipswich, MA) near the 3’ end of *GFP* using primers OAR210 and OAR211.

In most cases, introns were amplified using high fidelity polymerase directly from wild-type N2 genomic DNA using primers whose 3’ half matches the sequence at the end of the intron and whose 5’ ends match *unc-54* sequences flanking the site of intron insertion. For the *daao-1* and *rpl-14* introns, the template was an intermediate PCR product made using primers OAR237-240 that match exon sequences near the target intron. The 51 nt synthetic intron was made as two complementary primers OAR258 and OAR259 that were annealed to each other. The introns were inserted between *unc-54* exons 1 and 2 by Gibson assembly into an *unc-54p: unc-54::GFP* fusion. The resulting plasmids were sequenced to verify accurate insertion and the absence of mutations before the intron-containing *unc-54p: unc-54::GFP* fusion was inserted as a *Bgl*II-*Spe*I fragment into the vector pCFJ151^56^, which contains the *unc-119* marker gene.

### Mos1-mediated single copy insertion (MosSCI)

The intron-containing *unc-54:GFP* fusions were integrated into the ttTi5605 site on chromosome 2 in strain EG6699 by MosSCI^56,57^. The injections were performed by InVivo Biosystems (Eugene, OR, formerly known as Knudra Transgenics or NemaMetrix). The resulting worms were screened for progeny that had integrated the *unc-119* gene and lost the *mCherry* markers in the injection mix, and strains homozygous for the transgene insertion were obtained. To verify single-copy integration, DNA from each line was isolated using the DNeasy blood and tissue kit (Qiagen), digested with *Spe*I and *Hin*dIII, and subjected to gel blot analysis with a ^32^P labeled GFP probe and detected using a Storm phosphoimager, as shown in Supplementary Fig. S4. Transgene copy number was estimated by comparing band intensity using ImageQuant software (Molecular Dynamics). All single-copy lines obtained were used and given equal weight in calculations.

### Quantification of GFP in populations of worms

L1 stage worms were collected off starved plates using water, concentrated by brief centrifugation, and 125 µL of resuspended worms were placed in each well of a 96-well plate. Fluorescence was measured on a Perkin Elmer Model 2030-0050 Multilabel Plate Reader at an emission wavelength of 535 nm with an excitation wavelength of 485 nm (to measure GFP) or 355 nm (to correct for variation in the number of worms in each well using autofluorescence). For every line, at least six technical replicates were performed on each of three or more separate days. The ratio of the signals obtained with excitation at 485 nm to that at 355 nm was normalized by setting the level for untransformed worms equal to zero and the ratio in transgenic worms containing the intronless control to one. The values for intron-containing constructs represent the average fold increase in above-background fluorescence caused by the intron.

### Quantification of GFP in individual worms

Worms from a starved plate were grown on a fresh plate at room temperature for 17–18 h so that the population was predominantly at the L3 stage. L3 worms were picked and anaesthetized in a 0.25% solution of tetramisole on a 2% agar pad and examined using a UPlan FL 40x, 0.75 numerical aperture objective on an Olympus BX60 compound microscope, with a Hamamatsu Orca 12-bit digital camera and Micromanager software^58^. Single plane images were acquired in brightfield and fluorescent image at 150 ms exposure. The resulting images were then quantified using ImageJ software version 1.53^59^ using the segmented line tool with a width of 10 pixels to trace the muscle of each worm starting from the base of the grinder to the tip of the nose and back to the grinder on the opposite side of the worm. The mean reading per pixel for the entire line was corrected for background by subtracting the mean of a line drawn outside the worm parallel to and roughly the same length as the pharynx. Measurements were obtained from at least three biological replicates totaling more than 40 individual worms for each line. To calculate the fold increase in fluorescence caused by each intron, the average reading from untransformed worms was subtracted from the average readings for all transgenic lines containing the same construct, and the individual readings of lines with an intron-containing construct were divided by the average reading of the intronless control lines.

### Statistics

For all tests of significant differences between groups (i.e. lines, introns) we used a linear model in R, *lm(value ~ group)*. Model assumptions (e.g. normality of residuals) were validated by visualizing model diagnostic plots. Significant differences (two-sided test) among groups were determined using Tukey Honest Significant Difference (*TukeyHSD* function in R) at *alpha* = 0.05, after Bonferroni correction for multiple testing.

### RNA isolation and RNA gel blots

Worms from a starved plate were grown on a fresh plate at room temperature for 17 h so that the population was predominantly at the L3 stage. One mL of water was used to rinse the worms off 2 plates and pooled in a microfuge tube with the worms from another 2 plates. The tubes were centrifuged at 8,000 rpm (5,000 g) for 5 s and the supernatant was discarded. The worms were rinsed in 1 ml 0.1% SDS by vortexing and centrifuging as above and the supernatant was removed leaving the worms in a volume of 50 µL or less. The worms were resuspended in 100 µL worm lysis buffer (50 mM KCl, 10 mM TRIS pH 8.3, 2.5 mM MgCl_2_, 0.45% Tween 20) containing proteinase K at a final concentration of 1 mg/mL (30–40 units/mL) and incubated at 55 °C for ten minutes with mixing every 2–3 min. RNA was isolated using RNeasy Plus kits (Qiagen) following the manufacturers protocol. In short, this involved adding 350 µL buffer RLT Plus containing DTT to the lysed worms, mixing, and pipetting onto a gDNA removal column, which was spun for 30 s at 10,000 rpm (8,000 g). The flow-through was mixed with 350 µL 70% ethanol and transferred to an RNeasy column, which was spun 15 s at 10,000 rpm (8,000 g). The column was rinsed once with 700 µL buffer RW1 and twice with 500 µL buffer RPE, then dried by spinning at top speed for 1 min. RNA was eluted from the column with two washes with 50 µL RNAse free water, which were combined. The yield of RNA (typically 5–15 µg) was determined by spectrophotometer, the RNA was concentrated by ethanol precipitation, and the dried RNA was resuspended in RNAse free water.

2 µg of RNA from each line was used in RNA gel blots performed using reagents from the Northern Max kit (Ambion), hybridized with a ^32^P labeled GFP probe, and detected using a Storm phosphoimager. Band intensity was measured using ImageQuant software and corrected for small differences in loading using ethidium bromide stained rRNA bands quantified using BioRad software.

### Computational

Master files containing the sequences of all the introns in *C. elegans* or Arabidopsis, as well as the distance between the transcription start site and the start of the intron, were extracted from the annotated genomes of those organisms. As in the IMEter algorithm^34^, the frequency of occurrence of all possible k-mers was determined separately in the two populations of introns less than or greater than a threshold distance from the transcription start site of the gene in which the intron is located. The frequency of each k-mer is calculated as the count for that k-mer divided by the counts for all k-mers found in that population of sequences. Therefore, the sum of the frequency of all k-mers in each population is 1. The difference between the two frequency distributions was determined as the sum of the absolute values of the difference in frequency of each individual k-mer in the promoter-proximal and distal intron populations.

## Electronic supplementary material

Below is the link to the electronic supplementary material.

Supplementary Material 1

## Data Availability

The software for comparing kmer frequency distributions is available from GitHub. The plate reader, pharynx fluorescence, and RNA gel blot raw data on which the gene expression calculations are based can be found in Supplementary Tables S4, S5, and S6. The images used to measure pharynx fluorescence or any other original data, and all strains used in this study, are available upon request from ABR (abrose@ucdavis.edu) or LSR (lsrose@ucdavis.edu).
