# Supplementary material for: Introns increase gene expression in Caenorhabditis elegans by a mechanism that must be at least partly different than in plants
Source: Sci Rep. 2025 May 7;15:15862. doi: 10.1038/s41598-025-99739-6 (PMC12055998; doi:10.1038/s41598-025-99739-6)
Supplement: Supplementary file 1 — Supplementary Material 1 [file 41598_2025_99739_MOESM1_ESM.pdf]

Fig. S1

*fold change = (observation - mean(Un-Trans))/(mean(No Intron)-mean(Un-Trans))

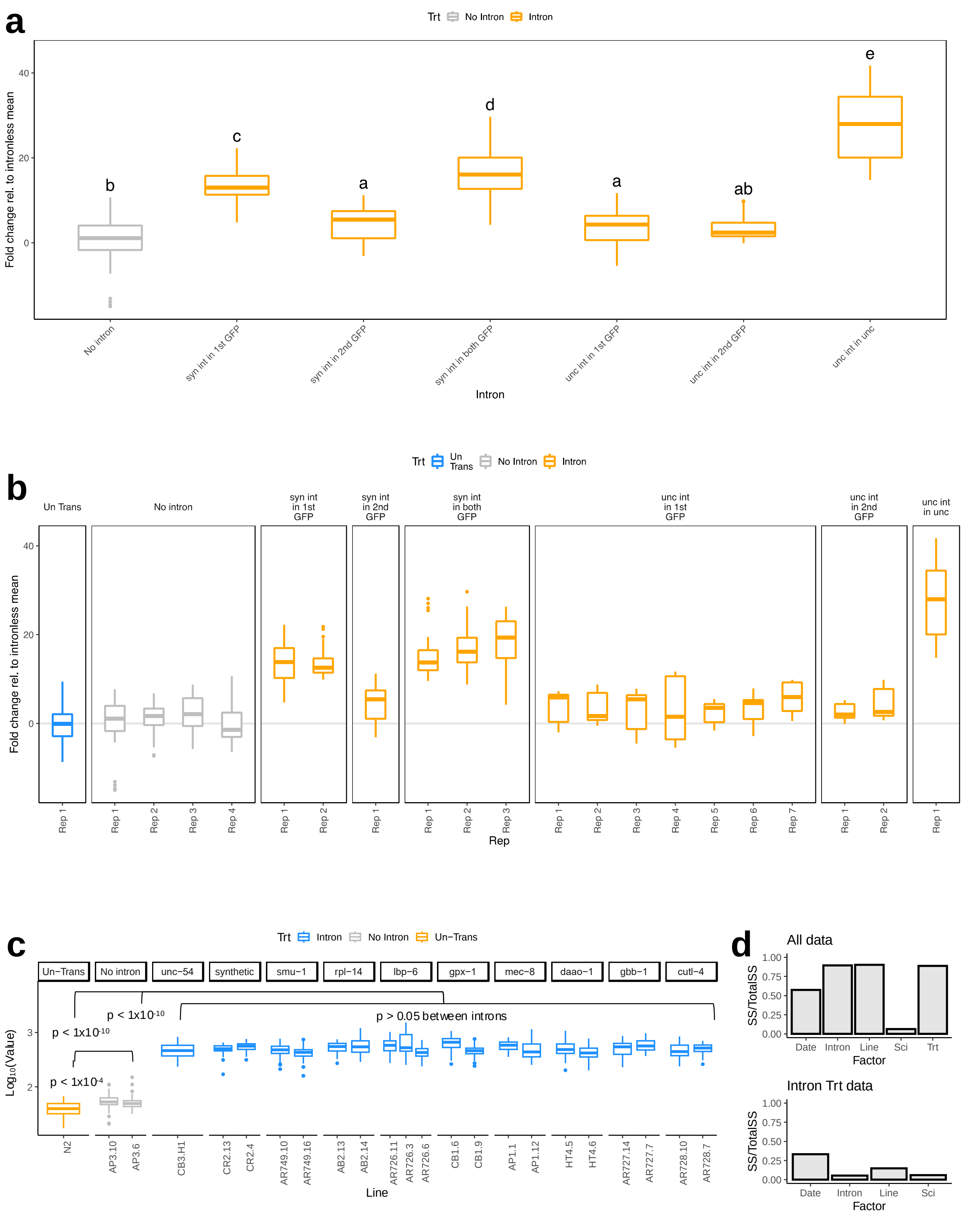

Fig. S1. Statistical analysis of the constructs shown in Fig. 1. a. Combined results for all lines containing the constructs shown in Figs. 1a and 1c. The p-values for all pairwise comparisons between constructs are in Supplementary Table S1. b. Results shown separately for each independent single-copy transgenic line (Rep) containing the constructs shown in Figs. 1a and 1c. c. Summary of fluorescence readings for all single-copy transgenic lines containing the constructs shown in Fig. 1e. Note that the scale is logarithmic to more clearly display the difference between untransformed worms and those containing the intronless control. The source of the intron in each construct is shown along the top of the figure and the name of each line is at the bottom. d. The variability in the data shown in c contributed by the date of the experiment, the intron present in the construct, the variation between lines containing the same construct, the scientist performing the experiment, or the treatment (Trt, meaning untransformed, intronless, or intron-containing). The upper graph compares all lines, while the lower graph shows only lines with an intron-containing transgene. SS is sum of squares.

Fig. S2

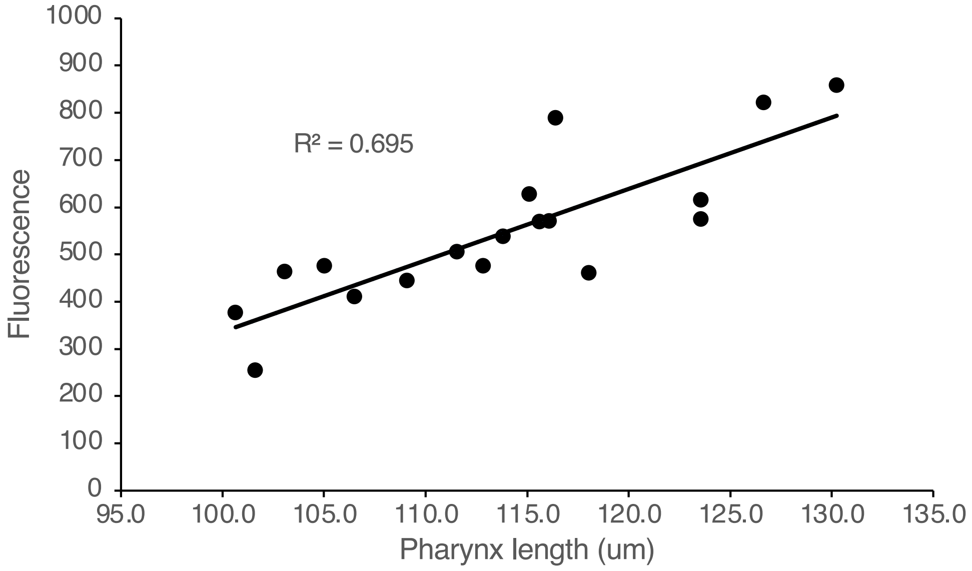

Fig. S2. GFP fluorescence correlates with pharynx length. Fluorescence measurements of 18 worms of line AR726.3 taken from the same plate on April 27, 2023 are shown relative to the length of the pharynx of each worm measured along the esophagus from the tip of the nose to the base of the grinder.

Fig. S3.

a

agatctgtttaagaaccattaatcaaactggtattctattttcactggttgtatgtaaacattctatctt
attccttttatcactgttctgcactttcctataaaaaaagttgaccgaccgtactctctgaattcatttt
tcccgatcttaccaactcccgatctatctctatccctggtttttcttcgtgctccaatggaattcttga
gacttccactatcttctctggcaccctccactacgcgtaggcgtctctcgcttcgtgtattcccgggaag
ccgggttcccgtctctcccgccgctgccgctgccgcacacagctttacacctcgtagaatccccaaagag
gggcgtggcttgcgggtgccaacatcctcctgccgaggaagaagcaggcactcatcactcgcatcatcaa
cctcgactaagggagcaccacattagttttgaggtagagaaaccatttgaaagaagcgagaaatcATGGA
GCACGAGAAGGACCCAGGATGGCAATATCTCCGCCGTACCAGAGAGCAGGTTTTGGAGGATCAATCTGGA
TCTGTAGCAACAATGAGTAAAGGAGAAGAACTTTTCACTGGAGTTGTCCCAATTCTTGTTGAATTAGATG
GTGATGTTAATGGGCACAAATTTTCTGTCAGTGGAGAGGGTGAAGGTGATGCAACATACGGAAAACTTAC
CCTTAAATTTATTTGCACTACTGGAAAACTACCTGTTCCATGGgtaagtttaaacatatatatactaact
aaccctgattatttaaattttcagCCAACACTTGTCACTACTTTCTGTTATGGTGTTCAATGCTTCTCGA
GATACCCAGATCATATGAAACGGCATGACTTTTTCAAGAGTGCCATGCCCGAAGGTTATGTACAGGAAAG
AACTATATTTTTCAAAGATGACGGGAACTACAAGACACgtaagtttaaacagttcggtactaactaacca
tacatatttaaattttcagGTGCTGAAGTCAAGTTTGAAGGTGATACCCTTGTTAATAGAATCGAGTTAA
AAGGTATTGATTTTAAAGAAGATGGAAACATTCTTGGACACAAATTGGAATACAACTATAACTCACACAA
TGTATACATCATGGCAGACAAACAAAAGAATGGAATCAAAGTTgtaagtttaaacatgattttactaact
aactaatctgatttaaattttcagAACTTCAAAATTAGACACAACATTGAAGATGGAAGCGTTCAACTAG
CAGACCATTATCAACAAAATACTCCAATTGGCGATGGCCCTGTCCTTTTACCAGACAACCATTACCTGTC
CACACAATCTGCCCTTTCGAAAGATCCCAACGAAAAGAGAGACCACATGGTCCTTCTTGAGTTTGTAACA
GCTGCTGGGATTACACATGGCATGGATGAACTATACAAAGGATCTGTAGCAACAATGAGTAAAGGAGAAG
AACTTTTCACTGGAGTTGTCCCAATTCTTGTTGAATTAGATGGTGATGTTAATGGGCACAAATTTTCTGT
CAGTGGAGAGGGTGAAGGTGATGCAACATACGGAAAACTTACCCTTAAATTTATTTGCACTACTGGAAAA
CTACCTGTTCCATGGgtaagtttaaacatatatatactaactaaccctgattatttaaattttcagCCAA
CACTTGTCACTACTTTCTGTTATGGTGTTCAATGCTTCTCGAGATACCCAGATCATATGAAACGGCATGA
CTTTTTCAAGAGTGCCATGCCCGAAGGTTATGTACAGGAAAGAACTATATTTTTCAAAGATGACGGGAAC
TACAAGACACgtaagtttaaacagttcggtactaactaaccatacatatttaaattttcagGTGCTGAAG
TCAAGTTTGAAGGTGATACCCTTGTTAATAGAATCGAGTTAAAAGGTATTGATTTTAAAGAAGATGGAAA
CATTCTTGGACACAAATTGGAATACAACTATAACTCACACAATGTATACATCATGGCAGACAAACAAAAG
AATGGAATCAAAGTTgtaagtttaaacatgattttactaactaactaatctgatttaaattttcagAACT
TCAAAATTAGACACAACATTGAAGATGGAAGCGTTCAACTAGCAGACCATTATCAACAAAATACTCCAAT

TGGCGATGGCCCTGTCCTTTTACCAGACAACCATTACCTGTCCACACAATCTGCCCTTTCGAAAGATCCC
AACGAAAAGAGAGACCACATGGTCCTTCTTGAGTTTGTAACAGCTGCTGGGATTACACATGGCATGGATG
AACTATACAAAtagggatccccgggtaccgagctccatctcgcgcccgtgcctctgacttctaagtccaa
ttactcttcaacatccctacatgctctttctccctgtgctcccacccccctatttttgttattatcaaaaa
acttctcttaatttctttgtttttttagcttcttttaagtcacctctaacaatgaaattgtgtagattcaa
aaatagaattaattcgtaataaaaagtcgaaaaaaattgtgctccctccccccattaataataattctat
cccaaaatctacacaatgttctgtgtacacttcttatgtttttttacttctgataaatttttttgaaacat
catagaaaaaaccgcacacaaaataccttatcatatgttacgtttcagtttatgaccgcaatttttattt
cttcgcacgtctgggcctctcatgacgtcaaatcatgctcatcgtgaaaaagttttggagtatttttgga
atttttcaatcaagtgaaagtttatgaaattaattttcctgcttttgctttttggggtttcccctattgt
ttgtcaagatttcgaggacggcgtttttcttgctaaaatcacaagtattgatgagcacgatgcaagaaag
atcggaagaaggtttgggtttgaggctcagtggaaggtgagtagaagttgataatttgaaagtggagtag
tgtctatggggtttttgccttaaatgacagaatacattcccaatataccaaacataactgtttcctacta
gt

b

agatctgtttaagaaccattaatcaaactggtattctattttcactggttgtatgtaaacattctatctt
attccttttatcactgttctgcactttcctataaaaaaagttgaccgaccgtactctctgaattcatttt
tcccgatcttaccaactcccgatctatctctatccctggtttttcttcgtgctccaatggaattcttga
gacttccactatcttctctggcaccctccactacgcgtaggcgtctctcgcttcgtgtattcccgggaag
ccgggttcccgtctctcccgccgctgccgctgccgcacacagctttacacctcgtagaatccccaaagag
gggcgtggcttgcgggtgccaacatcctcctgccgaggaagaagcaggcactcatcactcgcatcatcaa
cctcgactaagggagcaccacattagttttgaggtagagaaaccatttgaaagaagcgagaaatcATGGA
GCACGAGAAGGACCCAGGATGGCAATATCTCCGCCGTACCAGAGAGCAGGTTTTGGAGGATCAATCTGGA
TCCGTAGCAACAATGAGTAAAGGAGAAGAACTTTTCACTGGAGGGATTTGTCCCAATTCTTGTTGAATTA
GATGGTGATGTTAATGGGCACAAATTTTCTGTCAGTGGAGAGGGTGAAGGTGATGCAACATACGGAAAAC
TTACCCTTAAATTTATTTGCACTACTGGAAAACTACCTGTTCCATGGCCAACACTTGTCACTACTTTCTG
TTATGGTGTTCAATGCTTCTCGAGATACCCAGATCATATGAAACGGCATGACTTTTTCAAGAGTGCCATG
CCCGAAGGTTATGTACAGGAAAGAACTATATTTTTCAAAGATGACGGGAACTACAAGACACGTGCTGAAG
TCAAGTTTGAAGGTGATACCCTTGTTAATAGAATCGAGTTAAAAGGTATTGATTTTAAAGAAGATGGAAA
CATTCTTGGACACAAATTGGAATACAACTATAACTCACACAATGTATACATCATGGCAGACAAACAAAAG
AATGGAATCAAAGTTAACTTCAAAATTAGACACAACATTGAAGATGGAAGCGTTCAACTAGCAGACCATT
ATCAACAAAATACTCCAATTGGCGATGGCCCTGTCCTTTTACCAGACAACCATTACCTGTCCACACAATC
TGCCCTTTCGAAAGATCCCAACGAAAAGAGAGACCACATGGTCCTTCTTGAGTTTGTAACAGCTGCTGGG
ATTACACATGGCATGGATGAACTATACAAAtagagatccccgggtaccgagctccatctcgcgcccgtgc
ctctgacttctaagtccaattactcttcaacatccctacatgctctttctccctgtgctcccacccccta
tttttgttattatcaaaaaacttctcttaatttctttgtttttagcttcttttaagtcacctctaacaa
tgaaattgtgtagattcaaaaatagaattaattcgtaataaaaagtcgaaaaaaattgtgctccctcccc
ccattaataataattctatcccaaaatctacacaatgttctgtgtacacttcttatgtttttttacttctg
ataaattttttgaaacatcatagaaaaaaccgcacacaaaataccttatcatatgttacgtttcagttt
atgaccgcaatttttatttcttcgcacgtctgggcctctcatgacgtcaaatcatgctcatcgtgaaaaa
gttttggagtatttttggaatttttcaatcaagtgaaagtttatgaaattaattttcctgcttttgcttt
ttggggtttcccctattgtttgtcaagatttcgaggacggcgtttttcttgctaaaatcacaagtattga
tgagcacgatgcaagaaagatcggaagaaggtttgggtttgaggctcagtggaaggtgagtagaagttga
taatttgaaagtggagtagtgtctatggggtttttgccttaaatgacagaatacattcccaatataccaa
acataactgtttcctactagt

Fig. S3. Sequence of *unc-54:GFP* constructs.

a. Sequence of the constructs shown in Fig. 1a and 1c. The sequence extends from the *Bgl*II site in the *unc-54* promoter to the *Spe*I site downstream of the *unc-54* terminator. Promoter, terminator, and intron sequences are in lower case, while coding sequences are in upper case, with *unc-54* sequences in red and *GFP* in green text. The sequence shown is that of the bottom construct in Fig. 1a. The small arrows show the transcription start and stop sites, and the triangles indicate the insertion sites of *unc-54* intron 1 in the constructs shown in Fig 1c. **b**. Sequence of the constructs shown in Fig. 1e. The site of intron insertion is indicated by the triangle and all other explanations are as in **a.**

Fig. S4.

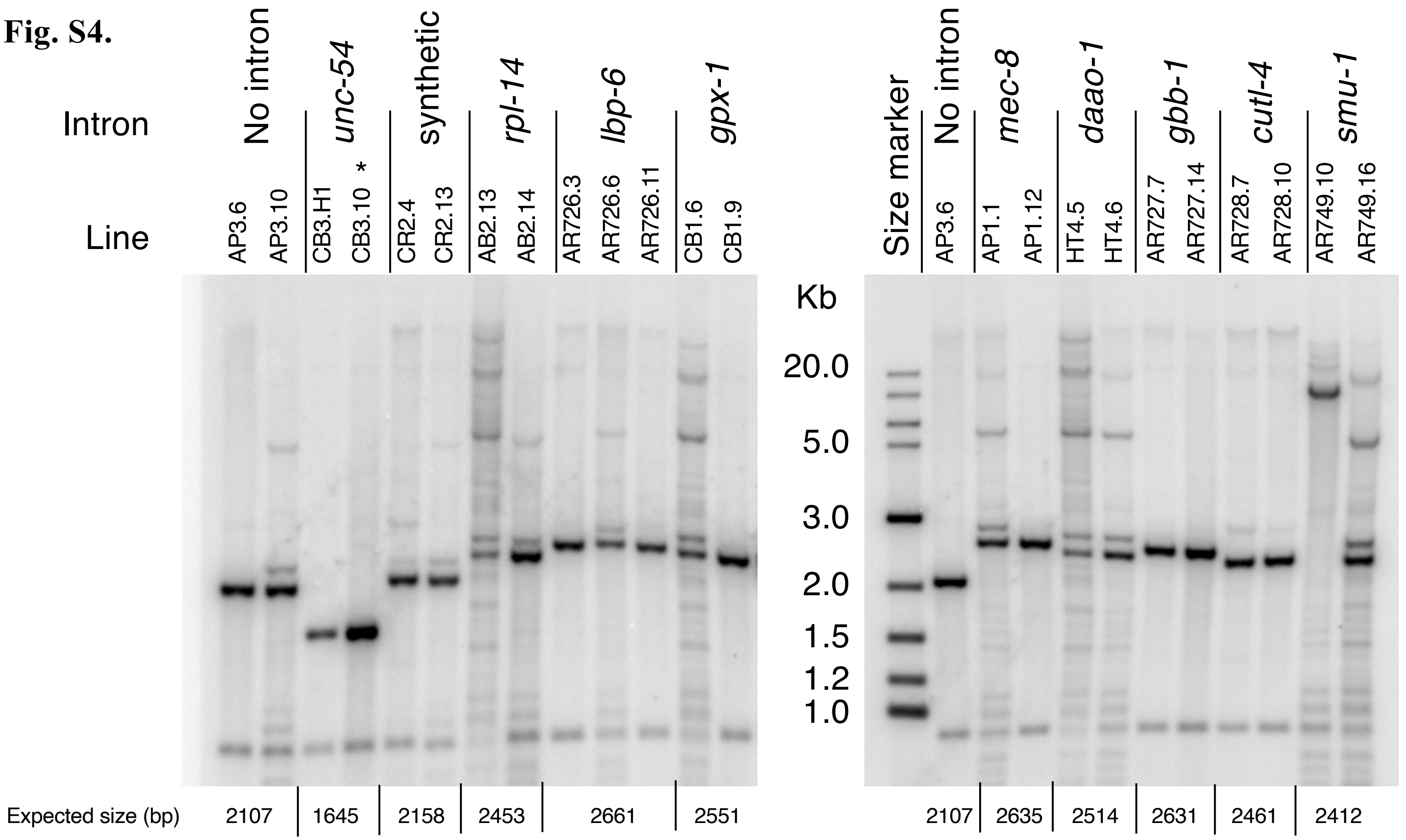

Fig. S4. Genomic DNA gel blots of transgenic worms. Genomic DNA was digested with *Spe*I and *Hin*dIII and probed with *GFP*. The expected size of the *GFP*-hybridizing band varies with intron size and is indicated along the bottom. The intron from *unc-54* is the only one that contains a *Hin*dIII site, creating a band smaller than in the intronless control. The band intensity in line CB3.10 (marked with an asterisk) is roughly twice that in the other lines, indicating a 2-copy insertion.

Fig. S5

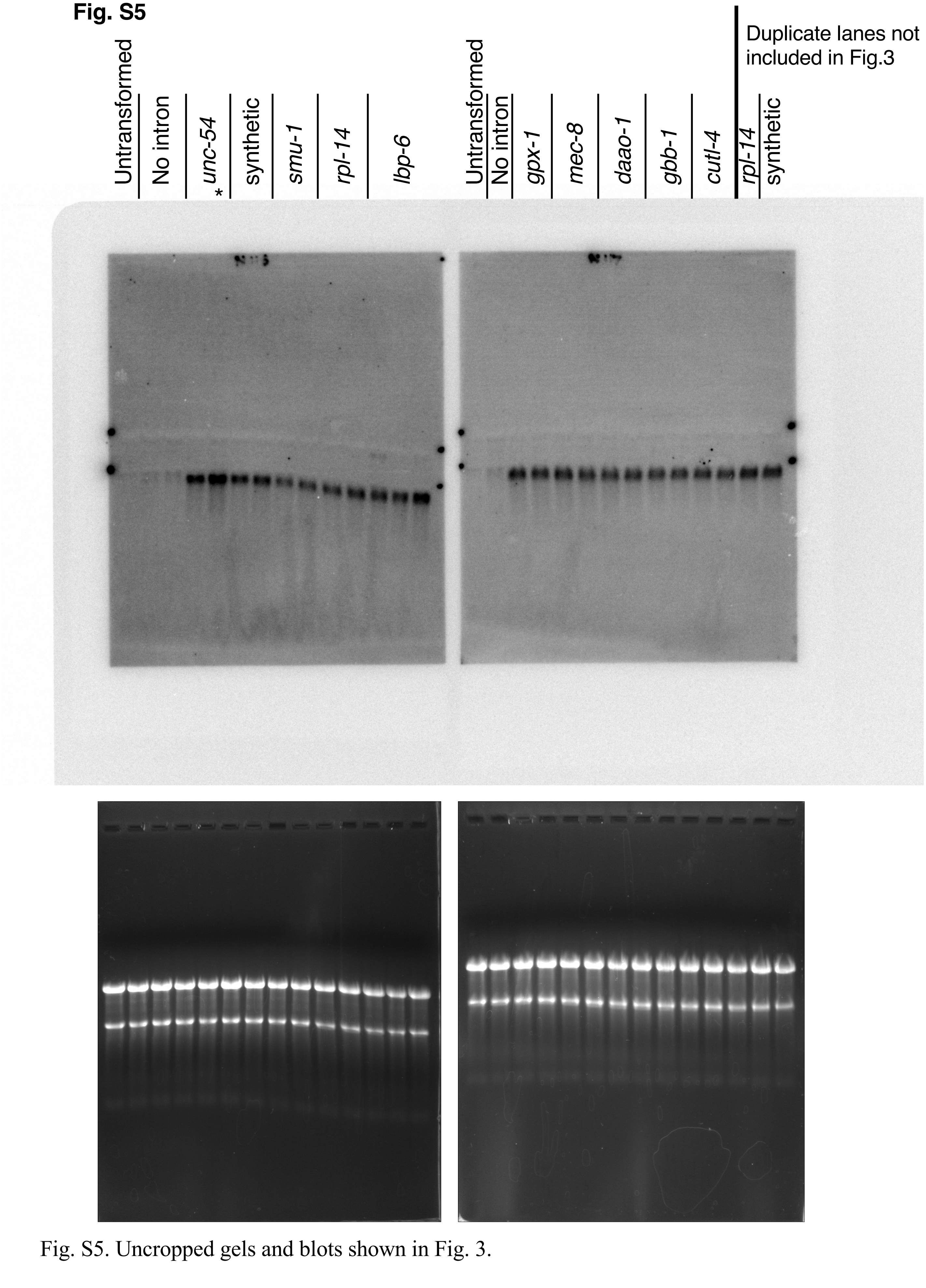

Fig. S5. Uncropped gels and blots shown in Fig. 3.

Table S1. Statistical comparison of fluorescence in worms containing the constructs shown in Figures 1a and 1c.

Pairwise comparison	Difference	Lower	Upper	Adj. p value
No intron vs synthetic introns in 1st GFP	12.57	10.57	14.57	1.92E-10
No intron vs synthetic introns in 2nd GFP	3.25	0.56	5.94	0.007
No intron vs synthetic introns in both GFPs	15.76	14.05	17.47	1.92E-10
No intron vs unc intron in unc sequences	26.54	24.21	28.86	1.92E-10
No intron vs unc intron in 1st GFP	2.59	1.07	4.12	1.33E-05
No intron vs unc intron in 2nd GFP	2.44	-0.04	4.92	0.058
Synthetic introns in 1st GFP vs synthetic introns in 2nd GFP	9.32	6.21	12.43	1.92E-10
Synthetic introns in 1st GFP vs synthetic introns in both GFPs	3.19	0.88	5.51	0.001
Synthetic introns in 1st GFP vs unc intron in unc sequences	13.97	11.17	16.76	1.92E-10
Synthetic introns in 1st GFP vs unc intron in 1st GFP	9.98	7.80	12.16	1.92E-10
Synthetic introns in 1st GFP vs unc intron in 2nd GFP	10.13	7.20	13.06	1.92E-10
Synthetic introns in 2nd GFP vs synthetic introns in both GFPs	12.51	9.58	15.45	1.92E-10
Synthetic introns in 2nd GFP vs unc intron in unc sequences	23.29	19.96	26.62	1.92E-10
Synthetic introns in 2nd GFP vs unc intron in 1st GFP	0.66	-2.17	3.48	0.99
Synthetic introns in 2nd GFP vs unc intron in 2nd GFP	0.81	-2.63	4.25	0.99
Synthetic introns in both GFPs vs unc intron in unc sequences	10.77	8.17	13.37	1.92E-10
Synthetic introns in both GFPs vs unc intron in 1st GFP	13.17	11.25	15.09	1.92E-10
Synthetic introns in both GFPs vs unc intron in 2nd GFP	13.32	10.58	16.07	1.92E-10
Unc intron in unc sequences vs unc intron in 1st GFP	23.94	21.46	26.42	1.92E-10
Unc intron in unc sequences vs unc intron in 2nd GFP	24.10	20.93	27.26	1.92E-10
Unc intron in 1st GFP vs unc intron in 2nd GFP	0.15	-2.48	2.78	0.999998

Table S2. Data for each individual line tested.

Intron	Description	Construct	Line	Fluorescence units Ave.	St. Dev.	n	Relative fluorescence	Relative RNA
	Wild-type untransformed worms		N2	**40**	**12**	**47**	**0**	
none	Intronless control	AP3		**53**	**16**	**149**	**1 +/- 1.2**	**1.03 +/- 0.07**
			RL381 AP3.6	52	16	76	0.9 +/- 1.2	1
			RL382 AP3.10	55	16	73	1.1 +/- 1.2	1.05 +/- 0.09
unc-54	In natural location	CB3		**487**	**149**	**59**	**33.6 +/- 11.2**	**16.7 +/- 2.9**
			RL377 CB3.H1	487	149	59	33.6 +/- 11.2	16.7 +/- 2.9
			RL421 CB3.10 *2-copy	1402	447	41	102.2 +/- 33.5	27.0 +/- 2.8
Synthetic	Small synthetic intron	CR2		**526**	**95**	**112**	**36.5 +/- 7.1**	**14.2 +/- 2.9**
			RL401 CR2.4	547	95	58	38.0 +/- 7.1	14.8 +/- 3.9
			RL386 CR2.13	504	90	54	34.8 +/- 6.8	13.4 +/- 0.6
From smu-1	PATC intron	AR749		**455**	**116**	**111**	**31.1 +/- 8.7**	**9.8 +/- 2.1**
			RL394 AR749.10	484	129	47	33.3 +/- 9.7	10.7 +/- 2.3
			RL395 AR749.16	433	101	64	29.5 +/- 7.6	9.0 +/- 2.3
From rpl-14	From gene expressed at high level	AB2		**561**	**170**	**97**	**39.1 +/- 12.7**	**13.8 +/- 2.3**
			RL390 AB2.13	544	125	48	37.8 +/- 9.4	13.5 +/- 3.2
			RL392 AB2.14	578	205	49	40.4 +/- 15.4	14.2 +/- 0.9
From lbp-6	From gene expressed at high level	AR726		**559**	**243**	**146**	**38.9 +/- 18.1**	**13.8 +/- 9.0**
			RL425 AR726.3	695	344	41	49.1 +/- 25.8	13.3 +/- 5.0
			RL379 AR726.6	442	118	60	30.2 +/- 8.9	11.9 +/- 8.1
			RL423 AR726.11	590	176	45	41.3 +/- 13.2	15.8 +/- 12.7
From gpx-1	From gene expressed at medium level	CB1		**553**	**170**	**105**	**38.5 +/- 12.8**	**12.4 +/- 2.7**
			RL420 CB1.6	651	186	47	45.8 +/- 14.0	11.4 +/- 4.3
			RL385 CB1.9	473	102	58	32.5 +/- 7.6	13.4 +/- 0.1
From mec-8	From gene expressed at medium level	AP1		**546**	**180**	**92**	**38.0 +/- 13.5**	**13.2 +/- 3.7**
			RL399 AP1.1	575	111	49	40.1 +/- 8.3	11.8 +/- 5.4
			RL422 AP1.12	514	233	43	35.5 +/- 17.5	14.6 +/- 2.1
From daao-1	From gene expressed at low level	HT4		**477**	**141**	**129**	**32.8 +/- 10.6**	**13.1 +/- 3.6**
			RL387 HT4.5	508	143	71	35.1 +/- 10.7	15.4 +/- 3.3
			RL388 HT4.6	439	130	58	29.9 +/- 9.8	10.8 +/- 2.6
From gbb-1	From gene expressed at low level	AR727		**567**	**160**	**104**	**39.6 +/- 12.0**	**16.1+/- 5.3**
			RL378 AR727.7	600	157	60	42.0 +/- 11.8	16.9 +/- 7.9
			RL383 AR727.14	522	154	44	36.2 +/- 11.6	15.3 +/- 4.5
From cutl-4	From gene expressed at very low level	AR728		**502**	**119**	**101**	**34.6 +/- 8.9**	**15.1 +/- 6.9**
			RL400 AR728.7	518	95	55	35.9 +/- 7.2	18.1 +/- 10.2
			RL449 AR728.10	482	142	46	33.2 +/- 10.6	12.1 +/- 1.2

The relative fluorescence and relative RNA are given as the mean +/- standard deviation.

The numbers in bold include all the data from single-copy worms containing the same construct.

Table S3. Oligonucleotides used in this study

Primer name	Primer sequence	Purpose
rpl14F	CCAGAGAGCAGGTTTTGGAGGTAAGTCATTTATTAATTAAG	For using Gibson assembly to insert *rpl-14* intron 1 into *unc-54:GFP*
rpl14R	CATTGTTGCTACGGATCCAGATTGATCCTGAAAATAACAATTAGAAGCC	For using Gibson assembly to insert *rpl-14* intron 1 into *unc-54:GFP*
lbp6F	CCAGAGAGCAGGTTTTGGAGGTAAGAAAGTGCAAAG	For using Gibson assembly to insert *lbp-6* intron 1 into *unc-54:GFP*
lbp6R	CATTGTTGCTACGGATCCAGATTGATCCTGGGATAAAAAC	For using Gibson assembly to insert *lbp-6* intron 1 into *unc-54:GFP*
gpx1F	CCAGAGAGCAGGTTTTGGAGGTGAGTTTGAAATG	For using Gibson assembly to insert *gpx-1* intron 2 into *unc-54:GFP*
gpx1R	CATTGTTGCTACGGATCCAGATTGATCCTGGAATATTTTGTCG	For using Gibson assembly to insert *gpx-1* intron 2 into *unc-54:GFP*
mec8F	CCAGAGAGCAGGTTTTGGAGGTAAATTTGTTTTCTTTACG	For using Gibson assembly to insert *mec-8* intron 1 into *unc-54:GFP*
mec8R	CATTGTTGCTACGGATCCAGATTGATCCTGAAAATTTGAAG	For using Gibson assembly to insert *mec-8* intron 1 into *unc-54:GFP*
daao1F	CCAGAGAGCAGGTTTTGGAGGTAGGTTTACCTTAACTC	For using Gibson assembly to insert *daao-1* intron 2 into *unc-54:GFP*
daao1R	CATTGTTGCTACGGATCCAGATTGATCCTGGAAAAAAGTG	For using Gibson assembly to insert *daao-1* intron 2 into *unc-54:GFP*
gbb1F	CCAGAGAGCAGGTTTTGGAGGTAATTGTGATTTTTG	For using Gibson assembly to insert *gbb-1* intron 1 into *unc-54:GFP*
gbb1R	CATTGTTGCTACGGATCCAGATTGATCCTGAAATTTAGAACGG	For using Gibson assembly to insert *gbb-1* intron 1 into *unc-54:GFP*
cutl4F	CCAGAGAGCAGGTTTTGGAGGTGAGATTTTGTAGACG	For using Gibson assembly to insert *cutl-4* intron 1 into *unc-54:GFP*
cutl4R	CATTGTTGCTACGGATCCAGATTGATCCTGGAGAAATTACC	For using Gibson assembly to insert *cutl-4* intron 1 into *unc-54:GFP*
OAR258	CCAGAGAGCAGGTTTTGGAGGTAAGTTTAAACATATATATACTAACTAACCCTGA-<br>TTATTTAAATTTTCAGGATCAATCTGGATCCGTAGCAACAATG	For using Gibson assembly to insert a synthetic intron into *unc-54:GFP*
OAR259	CATTGTTGCTACGGATCCAGATTGATCCTGAAAATTTAAATAATCAGGGTTAGTT-<br>AGTATATATATGTTTAAACTTACCTCCAAAACCTGCTCTCTGG	For using Gibson assembly to insert a synthetic intron into *unc-54:GFP*
OAR262	CCAGAGAGCAGGTTTTGGAGGTAAGTTTTCTTATGGGAAAGAAGG	For using Gibson assembly to insert *smu-1* intron 3 into *unc-54:GFP*
OAR263	CATTGTTGCTACGGATCCAGATTGATCCTGAAAATCTATTTGAAAAATTTACGAGG	For using Gibson assembly to insert *smu-1* intron 3 into *unc-54:GFP*
OAR237	GGCAAGCTTGCTGCCATCGTCAACG	To amplify the region that includes *rpl-14* intron 1
OAR238	TGAGGATCCTTCAAGTTTCTGACGGTGC	To amplify the region that includes *rpl-14* intron 1
OAR239	GCCATTTCTTTGTGACGATGATGTGG	To amplify the region that includes *daao-1* intron 2
OAR240	GATTTCCGTCAGCTTGGTACTCATGG	To amplify the region that includes *daao-1* intron 2
OAR130	GGAAAGCTTAGATCTGTTTAAGAACCATTAATC	To put a *Hind* III site just upstream of the *Bgl* II site in the *unc-54* promoter
OAR131	ATAGGATCCAGATTGATCCTGAAATTACAATAATGG	To put a *Bam* HI site in *unc-54* exon 2
OAR132	ATAGGATCCAGATTGATCCTCCAAAACCTGCTCTCTGG	To put a *Bam* HI site in *unc-54* exon 2 and delete intron 1
OAR135	ATTGGTACCGAGCTCCATCTCGCGCCCGTGCC	To put *Kpn* I and *Sac* I sites at the 5' end of the *unc-54* terminator
OAR136	TCGGAATTCCGAATCATACATACATAACATAGAATTTCTGCA	To put an *Eco* RI site at the 3' end of the *unc-54* terminator
OAR210	ACACTCGAGCCATGGTCCTTCTTGAGTTTGTAACAGGTGAGTATTTAT	For using Gibson assembly to insert *unc-54* intron 1 near the 3' end of GFP
OAR211	CATGCATGCCATGTGTAATCCCAGCAGCTGAAATTACAATAATGG	For using Gibson assembly to insert *unc-54* intron 1 near the 3' end of GFP

Table S4. Plate reader raw data

Plate read 7/11/16

Water		N2 untransformed		pIS19 COP968		pIS19 COP969		pIS19 COP983		pIS19 COP984	
485 nm (GFP	355 nm (bkg)	485 nm (GFP	355 nm (bkg)	485 nm (GFP	355 nm (bkg)	485 nm (GFP	355 nm (bkg)	485 nm (GFP	355 nm (bkg)	485 nm (GFP	355 nm (bkg)
1686	1886	27331	123954	20308	111325	21894	81104	25173	87530	28758	119855
1422	1778	30412	135575	20496	112325	25099	84069	26429	93103	26937	110246
1396	1768	30470	133767	20340	111355	23208	84039	25771	88545	24801	105127
1510	1662	29752	129065	20848	110855	22370	79729	25281	90174	26413	106993
1494	1820	29604	129003	20170	113844	22598	76339	24320	81971	25631	106620
1304	1612	27857	121908	16857	95597	24511	84577	23266	80492	24857	105409

pIS15 COP964		pAR707-3		pAR707-4		pAR707-5		pAR707-11		pAR707-12	
485 nm (GFP	355 nm (bkg)	485 nm (GFP	355 nm (bkg)	485 nm (GFP	355 nm (bkg)	485 nm (GFP	355 nm (bkg)	485 nm (GFP	355 nm (bkg)	485 nm (GFP	355 nm (bkg)
43295	80036	20192	63908	21574	66672	22962	69816	21186	63075	22082	78286
41486	75278	17475	63332	22544	67452	18789	57588	24929	75270	24394	85004
38795	67296	17265	56505	23898	75262	21304	64206	23028	70476	24096	86193
38263	71117	18867	60148	22272	67448	20784	61775	23586	72850	23220	80364
41000	78140	17221	56147	24402	71699	20186	61703	22562	67036	25829	87922
35478	63378	19651	60260	22922	69464	19097	59293	22030	65551	21002	73216

pAR707-13		pAR707-14		pAR708-11		pAR708-12	
485 nm (GFP	355 nm (bkg)	485 nm (GFP	355 nm (bkg)	485 nm (GFP	355 nm (bkg)	485 nm (GFP	355 nm (bkg)
23620	80024	25259	87356	16897	58757	30414	87458
22980	70316	26709	90727	17733	59071	29980	87406
22494	72113	26211	90907	18497	62017	31829	90629
22934	69073	25001	85456	19329	61785	29746	88082
23150	70338	25853	87232	18171	60462	30710	87650
24310	76193	24256	81178	19147	62143	25809	76699

Plate read 8/15/16

Water		N2 untransformed		pIS19 COP968		pIS19 COP969		pIS19 COP983		pIS19 COP984	
485 nm (GFP	355 nm (bkg)	485 nm (GFP	355 nm (bkg)	485 nm (GFP	355 nm (bkg)	485 nm (GFP	355 nm (bkg)	485 nm (GFP	355 nm (bkg)	485 nm (GFP	355 nm (bkg)
1332	1802	38527	111087	21406	68545	25941	82702	26955	79897	31226	87282
1454	1622	36926	108989	22960	72950	25457	82990	29306	87226	29836	85348
1592	1764	32665	95119	22524	71113	29060	90761	27339	82666	29780	88180
1508	1616	32653	96416	19825	65469	27609	88062	28812	85478	30888	87097
1388	1542	33541	97171	21786	68691	24837	81022	27565	82648	29112	85268
1534	1650	28237	83699	19781	64068	24258	79267	26615	80820	28998	80874
pIS15 COP964		pAR707-3		pAR707-4		pAR707-5		pAR707-11		pAR707-12	
485 nm (GFP	355 nm (bkg)	485 nm (GFP	355 nm (bkg)	485 nm (GFP	355 nm (bkg)	485 nm (GFP	355 nm (bkg)	485 nm (GFP	355 nm (bkg)	485 nm (GFP	355 nm (bkg)
57304	108594	35002	112689	42280	134582	36032	103607	39303	121129	39441	115366
58513	112028	36254	116245	43279	138994	36060	103567	38585	120502	38841	117472
54923	105958	36400	116543	42262	137156	36636	104820	40354	124375	37064	114974
50342	94830	37202	119325	42451	136394	35602	101591	40464	125344	39449	118337
50044	96566	37647	121139	41378	133827	35658	102854	38781	121287	39811	116936
47125	92755	37072	119435	43127	138405	35464	102798	40390	124110	38415	116009
pAR707-13		pAR707-14		pAR708-11		pAR708-12					
485 nm (GFP	355 nm (bkg)	485 nm (GFP	355 nm (bkg)	485 nm (GFP	355 nm (bkg)	485 nm (GFP	355 nm (bkg)				
30832	94342	40586	114377	42875	135722	35912	122424				
30340	92779	39907	109974	44691	142527	38133	127917				
30946	95531	40464	113736	40946	132095	37112	125698				
31240	95097	40450	111658	40432	130402	35318	122310				
31827	94430	40548	113222	41356	132704	34545	120464				
30732	92795	36822	103375	41790	132555	34345	117691				

Plate read 8/24/16

Water		N2 untransformed		pIS19 COP968		pIS19 COP969		pIS19 COP983		pIS19 COP984	
485 nm (GFP	355 nm (bkg)	485 nm (GFP	355 nm (bkg)	485 nm (GFP	355 nm (bkg)	485 nm (GFP	355 nm (bkg)	485 nm (GFP	355 nm (bkg)	485 nm (GFP	355 nm (bkg)
1426	968	15301	66510	17743	66564	12974	53498	17513	58571	13528	49536
1422	994	15459	68247	18363	69708	13628	56886	17957	58989	13906	51189
1416	952	14995	59449	18483	70012	14074	53572	18285	68569	18471	66035
1344	996	15147	61340	16965	65739	14030	53232	18683	70474	17953	63578
1238	1076	13790	55623	17235	63133	12520	48327	16475	60604	16179	58591
1408	952	13076	54060	18285	66251	12242	47665	17267	64244	15577	57368

pIS15 COP964		pAR707-3		pAR707-4		pAR707-5		pAR707-11		pAR707-12	
485 nm (GFP	355 nm (bkg)	485 nm (GFP	355 nm (bkg)	485 nm (GFP	355 nm (bkg)	485 nm (GFP	355 nm (bkg)	485 nm (GFP	355 nm (bkg)	485 nm (GFP	355 nm (bkg)
33653	84551	22156	80782	25787	92250	26231	86125	28692	101369	35206	120502
30022	68887	23482	85630	24791	94232	26271	87714	28858	99483	33633	114861
31278	76126	29314	95701	29018	99779	26949	88769	31651	118732	37643	122012
30822	77820	29930	98208	29492	102066	28422	94372	33069	121776	38083	125354
36312	78628	22108	78262	26495	95675	28490	99447	28376	100832	21620	73879
34722	75590	22742	82193	27337	96884	30140	105489	27417	97525	21706	73543

pAR707-13		pAR707-14		pAR708-11		pAR708-12	
485 nm (GFP	355 nm (bkg)	485 nm (GFP	355 nm (bkg)	485 nm (GFP	355 nm (bkg)	485 nm (GFP	355 nm (bkg)
25121	100288	24511	96954	33723	114481	35542	124569
24100	91225	24336	93711	32033	113129	30354	102328
19949	72730	23078	94390	25651	88166	26155	85046
20244	70188	23922	96786	27791	93185	25581	86935
23928	88811	30028	119389	28634	98340	30738	102390
23520	88397	28840	112987	28838	100594	30330	100680

Plate read 9/15/16

Water		N2 untransformed		pIS19 COP968		pIS19 COP969		pIS19 COP983		pIS19 COP984	
485 nm (GFP	355 nm (bkg)	485 nm (GFP	355 nm (bkg)	485 nm (GFP	355 nm (bkg)	485 nm (GFP	355 nm (bkg)	485 nm (GFP	355 nm (bkg)	485 nm (GFP	355 nm (bkg)
938	588	17829	64891	21562	73643	20490	72320	20362	75084	20622	72384
1530	878	18349	67997	21616	72860	19923	71525	20290	74703	21316	72704
1318	750	18391	64406	18879	65769	21014	71251	19781	68955	22618	77724
1150	728	19491	67256	18963	64090	20988	70080	22074	75296	22536	79379
1318	734	20732	78108	23392	77175	23548	80858	19365	69225	19879	67965
1090	686	21414	76565	20254	66502	24677	82427	17957	62437	19619	66772
pIS18 COP985		pIS18 COP986		pIS21 3C		pIS20 31C		pIS20 32C		pIS20 34A	
485 nm (GFP	355 nm (bkg)	485 nm (GFP	355 nm (bkg)	485 nm (GFP	355 nm (bkg)	485 nm (GFP	355 nm (bkg)	485 nm (GFP	355 nm (bkg)	485 nm (GFP	355 nm (bkg)
27973	77283	24374	66408	26833	84381	22102	59153	30730	73164	28245	71523
27795	75762	25113	68561	26269	79111	23724	61757	28590	71831	27995	73050
32981	90490	25105	69584	15651	54861	31843	82045	26649	64857	25079	62203
31094	83705	26923	75914	16019	54935	31689	85452	26139	67596	25321	64548
34095	89207	30862	78350	17119	59507	21798	59687	27663	68657	26313	67396
35762	94546	29766	75772	16797	56594	21362	59991	26807	68221	26839	68585

Plate read 9/22/16

Water		N2 untransformed		pIS19 COP968		pIS19 COP969		pIS19 COP983		pIS19 COP984	
485 nm (GFP	355 nm (bkg)	485 nm (GFP	355 nm (bkg)	485 nm (GFP	355 nm (bkg)	485 nm (GFP	355 nm (bkg)	485 nm (GFP	355 nm (bkg)	485 nm (GFP	355 nm (bkg)
864	614	15723	54891	24539	75168	19429	64178	22106	70598	22890	70328
1488	842	16679	58659	23894	74451	19167	63860	21252	69442	22360	70148
1304	812	20610	73280	18319	56251	18121	62643	18119	55767	19303	63203
1396	824	20224	75828	18639	58559	18943	64662	18097	55157	18403	61634
1264	756	14995	54823	20072	62945	21378	68691	20176	63157	21772	66942
1180	740	15003	52401	20908	65845	21340	70066	20814	65159	21240	65439
pIS18 COP985		pIS18 COP986		pIS21 3C		pIS20 31C		pIS20 32C		pIS20 34A	
485 nm (GFP	355 nm (bkg)	485 nm (GFP	355 nm (bkg)	485 nm (GFP	355 nm (bkg)	485 nm (GFP	355 nm (bkg)	485 nm (GFP	355 nm (bkg)	485 nm (GFP	355 nm (bkg)
25299	64274	22020	58411	22120	67174	23188	68163	25419	69884	25599	62101
24879	65367	22836	61118	22972	69990	24521	68541	24857	70642	25345	62195
26467	69738	25707	70426	20786	63714	18857	52807	22792	60092	29934	72504
28059	72564	26313	73326	21806	67548	19579	54837	23432	62391	30638	74111
20172	55055	20252	56043	18171	58128	18575	52655	24124	57708	27219	64855
20880	56569	19483	52875	18877	61218	19197	54801	23626	57610	28646	67412

Plate read 10/6/16

Water		N2 untransformed		pIS19 COP968		pIS19 COP969		pIS19 COP983		pIS19 COP984	
485 nm (GFP	355 nm (bkg)	485 nm (GFP	355 nm (bkg)	485 nm (GFP	355 nm (bkg)	485 nm (GFP	355 nm (bkg)	485 nm (GFP	355 nm (bkg)	485 nm (GFP	355 nm (bkg)
1100	732	20718	68761	22164	68427	21926	64991	23370	72017	21548	62975
1508	908	21964	77109	20138	63189	21500	66031	24895	74900	22340	64967
1450	842	16495	62217	22362	72025	21100	66151	19903	60556	15267	48651
1488	908	17435	61478	21866	68503	20376	63141	21124	62831	15307	50192
1468	868	13810	47535	17983	55073	16293	51493	19851	62173	11966	38689
1540	890	13870	48975	17041	51659	16383	51351	20812	64424	12492	38843
pIS18 COP985		pIS18 COP986		pIS21 3C		pIS20 31C		pIS20 32C		pIS20 34A	
485 nm (GFP	355 nm (bkg)	485 nm (GFP	355 nm (bkg)	485 nm (GFP	355 nm (bkg)	485 nm (GFP	355 nm (bkg)	485 nm (GFP	355 nm (bkg)	485 nm (GFP	355 nm (bkg)
21800	65955	19845	52667	15649	52051	23426	61875	22544	62121	24244	64120
21362	64817	19563	51981	15095	52553	23754	63540	21712	61610	24855	66820
19807	60844	23492	63824	18401	61661	23450	60728	21928	57494	19465	54827
19639	60522	23264	64546	19229	62601	24705	64981	22318	58873	19293	54016
21566	64068	20320	53384	23746	72478	19159	48345	21028	54957	20802	65105
22642	66752	20580	54470	23298	70965	19645	50122	20424	52363	20800	61386

Plate #1 read 2/2/17

Water		N2 untransformed		pIS19 COP969		pIS19 COP983		pIS15 COP964		pIS18 COP985	
485 nm (GFP	355 nm (bkg)	485 nm (GFP	355 nm (bkg)	485 nm (GFP	355 nm (bkg)	485 nm (GFP	355 nm (bkg)	485 nm (GFP	355 nm (bkg)	485 nm (GFP	355 nm (bkg)
720	564	22918	75738	13790	47283	22712	71635	26757	55893	33159	78881
760	614	24815	82097	13790	44097	24679	76513	25609	56580	33809	81263
796	590	25597	76461	18113	56718	19245	63892	24038	50090	31532	72182
770	1426	25465	75658	17643	55655	18051	62403	23916	51335	28544	66768
1004	792	19459	66151	18281	66478	14599	51951	35590	78432	25555	64222
732	638	20258	66694	19067	66768	14038	51395	34445	69584	27135	67166
pIS18 COP986		pIS21 3C		pIS20 31C		pIS20 32C		pIS20 34A			
485 nm (GFP	355 nm (bkg)	485 nm (GFP	355 nm (bkg)	485 nm (GFP	355 nm (bkg)	485 nm (GFP	355 nm (bkg)	485 nm (GFP	355 nm (bkg)		
32529	77942	27021	84523	34836	88150	30796	75284	34864	77770		
29844	72003	28704	86313	34425	87596	31981	77349	33273	77706		
31090	80962	31452	87998	29562	74878	31316	78528	35040	77259		
28382	74545	28690	85070	29320	73697	32737	79395	35714	80616		
33457	77223	23424	70356	25367	65741	29252	69039	31104	68113		
30516	70969	22896	68547	27953	70843	31438	74579	28950	63972		

Plate #2 read 2/2/17

Water		N2 untransformed		pIS19 COP984	
485 nm (GFP	355 nm (bkg)	485 nm (GFP	355 nm (bkg)	485 nm (GFP	355 nm (bkg)
966	756	22862	76385	24010	80430
856	734	24549	81751	22000	74459
788	626	28784	99663	15207	55849
870	754	25871	90168	15061	52081
808	720	23536	80960	21078	76002
794	692	17803	64234	21062	73963

Plate read 2/28/17

Water		N2 untransformed		pIS19 COP983		pIS19 COP984	
485 nm (GFP	355 nm (bkg)	485 nm (GFP	355 nm (bkg)	485 nm (GFP	355 nm (bkg)	485 nm (GFP	355 nm (bkg)
666	522	22036	84779	27015	96318	16853	62013
1032	784	22058	84805	27191	99037	16051	58072
1166	824	15407	59825	17489	70955	26853	98132
958	750	17079	64642	14809	60612	22482	82982
1274	898	13554	53936	24336	92620	18091	72466
1110	764	14403	56311	23684	89249	18293	72708

Plate read 3/7/17

Water		N2 untransformed		pIS19 COP968		pIS19 COP969	
485 nm (GFP	355 nm (bkg)	485 nm (GFP	355 nm (bkg)	485 nm (GFP	355 nm (bkg)	485 nm (GFP	355 nm (bkg)
692	572	26753	91373	29786	103964	32319	113932
1172	766	31130	109283	25675	91195	33455	116593
784	662	20372	77792	24933	90418	25199	84605
1014	736	21416	74267	23448	87568	28454	97277
966	750	21492	76287	24787	91996	24589	81271
888	734	21602	79663	23956	88925	19823	67750
pIS19 COP983		pIS19 COP984		pIS15 COP964			
485 nm (GFP	355 nm (bkg)	485 nm (GFP	355 nm (bkg)	485 nm (GFP	355 nm (bkg)		
22826	74261	22712	85846	41064	80278		
22270	72392	22186	85354	36000	68285		
23486	76279	24647	96894	39487	77109		
23184	75538	22358	87456	39917	79729		
31256	105171	17555	72672	36524	65031		
32583	111015	18453	76729	37677	70993		

Plate read 3/21/17

Water		N2 untransformed		pIS19 COP968		pIS19 COP969	
485 nm (GFP	355 nm (bkg)	485 nm (GFP	355 nm (bkg)	485 nm (GFP	355 nm (bkg)	485 nm (GFP	355 nm (bkg)
806	820	22222	85708	23142	86453	24474	89658
1118	864	22576	85468	21922	80108	23446	85096
902	736	20440	74155	18971	72784	24867	90366
892	776	20446	75808	17945	69536	22536	83194
816	708	25453	102358	21254	73695	25417	89704
870	796	25289	99353	19139	65263	24617	86343
750	1226	30604	105537				
940	834	28049	94270				
938	1348	26953	98118				
920	766	27403	98286				
1094	882	32213	125805				
980	780	33229	125490				

pIS19 COP983		pIS19 COP984		pIS15 COP964	
485 nm (GFP	355 nm (bkg)	485 nm (GFP	355 nm (bkg)	485 nm (GFP	355 nm (bkg)
20392	73959	18405	69105	31733	81337
19487	71109	16583	65605	30896	80264
15877	60368	18045	67867	34738	83668
14975	56666	18397	68055	33865	81236
21188	74571	14963	58052	35132	83312
19843	67408	13742	53378	34009	79899

Table S5. Pharynx fluorescence raw data

Value	Line	Intron	Scientist	Date	Pharynx Mean	Background Mean
24.764	N2	Untransforme	AB	2/17/19	165.392	140.628
49.939	N2	Untransforme	AB	2/17/19	196.505	146.566
51.66	N2	Untransforme	AB	2/17/19	201.965	150.305
49.262	N2	Untransforme	AB	2/17/19	197.983	148.721
49.551	N2	Untransforme	AB	2/17/19	195.719	146.168
47.64	N2	Untransforme	AB	2/17/19	193.767	146.127
48.967	N2	Untransforme	AB	2/17/19	196.72	147.753
46.346	N2	Untransforme	AB	2/17/19	190.124	143.778
51.527	N2	Untransforme	AB	2/17/19	199.594	148.067
57.097	N2	Untransforme	AB	2/17/19	204.039	146.942
32.012	N2	Untransforme	AB	2/17/19	178.696	146.684
42.686	N2	Untransforme	AB	2/17/19	185.034	142.348
31.518	N2	Untransforme	AB	2/17/19	176.265	144.747
43.883	N2	Untransforme	AB	2/17/19	189.227	145.344
32.15	N2	Untransforme	AB	2/17/19	175.873	143.723
31.948	N2	Untransforme	AB	2/17/19	176.03	144.082
33.869	N2	Untransforme	AB	2/17/19	179.614	145.745
31.986	N2	Untransforme	AB	2/17/19	173.613	141.627
33.779	N2	Untransforme	AB	2/17/19	179.328	145.549
29.579	N2	Untransforme	AB	2/17/19	172.979	143.4
56.15	N2	Untransforme	LR	6/6/22	219.872	163.722
42.772	N2	Untransforme	LR	6/6/22	202.012	159.24
35.552	N2	Untransforme	LR	6/6/22	197.9	162.348
39.583	N2	Untransforme	LR	6/6/22	198.557	158.974
22.582	N2	Untransforme	LR	6/6/22	183.46	160.878
26.543	N2	Untransforme	LR	6/6/22	187.314	160.771
23.75	N2	Untransforme	LR	6/6/22	186.929	163.179
34.611	N2	Untransforme	LR	6/6/22	201.081	166.47
37.227	N2	Untransforme	LR	6/6/22	204.911	167.684
31.26	N2	Untransforme	LR	6/6/22	197.677	166.417
26.906	N2	Untransforme	LR	6/6/22	192.878	165.972
18.076	N2	Untransforme	LR	6/6/22	179.136	161.06
17.22	N2	Untransforme	LR	6/6/22	180.732	163.512
28.891	N2	Untransforme	AR	4/14/23	179.855	150.964
34.340	N2	Untransforme	AR	4/14/23	186.255	151.915
47.474	N2	Untransforme	AR	4/14/23	199.007	151.534
33.528	N2	Untransforme	AR	4/14/23	184.670	151.142
36.916	N2	Untransforme	AR	4/14/23	188.852	151.936
47.507	N2	Untransforme	AR	4/14/23	199.189	151.683
67.166	N2	Untransforme	AR	5/11/23	217.788	150.622
61.094	N2	Untransforme	AR	5/11/23	211.593	150.499
53.267	N2	Untransforme	AR	5/11/23	203.629	150.362
58.405	N2	Untransforme	AR	5/11/23	209.496	151.091
46.35	N2	Untransforme	AR	5/11/23	196.703	150.353
40.537	N2	Untransforme	AR	5/11/23	191.698	151.161
52.569	N2	Untransforme	AR	5/11/23	203.264	150.695
47.376	N2	Untransforme	AR	5/11/23	198.137	150.761

Value	Line	Intron	Scientist	Date	Pharynx Mean	Background Mean
31.674	AP3.6	No intron	AB	2/5/19	240.044	208.37
54.796	AP3.6	No intron	AB	2/5/19	195.151	140.355
37.996	AP3.6	No intron	AB	2/5/19	178.196	140.2
44.886	AP3.6	No intron	AB	2/5/19	184.381	139.495
41.935	AP3.6	No intron	AB	2/5/19	183.227	141.292
57.43	AP3.6	No intron	AB	2/5/19	198.878	141.448
38.037	AP3.6	No intron	AB	2/5/19	182.851	144.814
40.987	AP3.6	No intron	AB	2/5/19	182.944	141.957
53.146	AP3.6	No intron	AB	2/5/19	196.021	142.875
41.933	AP3.6	No intron	AB	2/5/19	183.002	141.069
39.797	AP3.6	No intron	AB	2/5/19	185.34	145.543
52.335	AP3.6	No intron	AB	2/5/19	197.017	144.682
60.253	AP3.6	No intron	AB	2/5/19	204.595	144.342
64.926	AP3.6	No intron	AB	2/5/19	212.187	147.261
45.037	AP3.6	No intron	AB	2/5/19	190.821	145.784
71.297	AP3.6	No intron	AB	2/5/19	216.797	145.5
46.577	AP3.6	No intron	AB	2/5/19	194.253	147.676
39.288	AP3.6	No intron	AB	2/5/19	184.699	145.411
55.84	AP3.6	No intron	AB	2/5/19	199.902	144.062
40.638	AP3.6	No intron	AB	2/5/19	187.113	146.475
56.916	AP3.6	No intron	AB	5/14/19	199.355	142.439
60.224	AP3.6	No intron	AB	5/14/19	209.904	149.68
47.455	AP3.6	No intron	AB	5/14/19	191.556	144.101
49.628	AP3.6	No intron	AB	5/14/19	199.515	149.887
52.333	AP3.6	No intron	AB	5/14/19	200.412	148.079
82.323	AP3.6	No intron	AB	5/14/19	232.201	149.878
53.622	AP3.6	No intron	AB	5/14/19	201.095	147.473
55.067	AP3.6	No intron	AB	5/14/19	201.723	146.656
52.739	AP3.6	No intron	AB	5/14/19	209.29	156.551
60.405	AP3.6	No intron	AB	5/14/19	207.194	146.789
48.787	AP3.6	No intron	AB	5/14/19	190.544	141.757
43.6	AP3.6	No intron	AB	5/14/19	191.324	147.724
41.987	AP3.6	No intron	AB	5/14/19	186.76	144.773
38.668	AP3.6	No intron	AB	5/14/19	185.397	146.729
39.338	AP3.6	No intron	AB	5/14/19	187.174	147.836
52.115	AP3.6	No intron	AB	5/14/19	200.07	147.955

Value	Line	Intron	Scientist	Date	Pharynx Mean	Background Mean
49.061	AP3.6	No intron	AB	6/25/19	194.734	145.673
55.241	AP3.6	No intron	AB	6/25/19	192.426	137.185
46.948	AP3.6	No intron	AB	6/25/19	186.554	139.606
47.335	AP3.6	No intron	AB	6/25/19	186.575	139.24
63.786	AP3.6	No intron	AB	6/25/19	200.372	136.586
37.443	AP3.6	No intron	AB	6/25/19	171.549	134.106
37.843	AP3.6	No intron	AB	6/25/19	176.56	138.717
43.792	AP3.6	No intron	AB	6/25/19	192.069	148.277
48.421	AP3.6	No intron	AB	6/25/19	191.301	142.88
44.067	AP3.6	No intron	AB	6/25/19	178.454	134.387
52.959	AP3.6	No intron	AB	6/25/19	201.035	148.076
54.086	AP3.6	No intron	AB	6/25/19	196.687	142.601
60.347	AP3.6	No intron	AB	6/25/19	203.964	143.617
49.72	AP3.6	No intron	AB	6/25/19	186.601	136.881
47.993	AP3.6	No intron	AB	6/25/19	180.665	132.672
55.313	AP3.6	No intron	AB	6/25/19	197.328	142.015
34.872	AP3.6	No intron	AB	6/25/19	179.742	144.87
36.708	AP3.6	No intron	LR	6/6/22	198.047	161.339
42.199	AP3.6	No intron	LR	6/6/22	199.248	157.049
48.106	AP3.6	No intron	LR	6/6/22	206.993	158.887
48.396	AP3.6	No intron	LR	6/6/22	208.939	160.543
45.656	AP3.6	No intron	LR	6/6/22	205.201	159.545
58.035	AP3.6	No intron	AR	4/14/23	205.211	147.176
50.472	AP3.6	No intron	AR	4/14/23	195.909	145.437
52.338	AP3.6	No intron	AR	4/14/23	200.655	148.317
47.475	AP3.6	No intron	AR	4/14/23	195.213	147.738
53.897	AP3.6	No intron	AR	4/14/23	201.118	147.222
62.264	AP3.6	No intron	AR	4/14/23	209.995	147.730
41.715	AP3.6	No intron	AR	4/14/23	189.116	147.401
39.315	AP3.6	No intron	AR	4/14/23	183.180	143.865
49.205	AP3.6	No intron	AR	4/14/23	197.057	147.852
47.851	AP3.6	No intron	AR	4/14/23	194.887	147.036
74.533	AP3.6	No intron	AR	5/11/23	226.733	152.2
149.766	AP3.6	No intron	AR	5/11/23	302.077	152.311
110.195	AP3.6	No intron	AR	5/11/23	262.614	152.419
61.234	AP3.6	No intron	AR	5/11/23	213.299	152.065
63.262	AP3.6	No intron	AR	5/11/23	214.014	150.752
61.315	AP3.6	No intron	AR	5/11/23	213.652	152.337
48.953	AP3.6	No intron	AR	5/11/23	198.93	149.977
49.941	AP3.6	No intron	AR	5/11/23	201.292	151.351

Value	Line	Intron	Scientist	Date	Pharynx Mean	Background Mean
63.723	AP3.10	No intron	AB	2/5/19	206.605	142.882
43.584	AP3.10	No intron	AB	2/5/19	187.925	144.341
32.392	AP3.10	No intron	AB	2/5/19	179.887	147.495
44.886	AP3.10	No intron	AB	2/5/19	187.416	142.53
48.823	AP3.10	No intron	AB	2/5/19	193.956	145.133
43.7	AP3.10	No intron	AB	2/5/19	187.749	144.049
36.141	AP3.10	No intron	AB	2/5/19	177.626	141.485
81.536	AP3.10	No intron	AB	2/5/19	228.085	146.549
39.171	AP3.10	No intron	AB	2/5/19	184.038	144.867
69.291	AP3.10	No intron	AB	2/5/19	212.181	142.89
49.953	AP3.10	No intron	AB	2/5/19	192.365	142.412
51.207	AP3.10	No intron	AB	2/5/19	192.68	141.473
47.248	AP3.10	No intron	AB	2/5/19	188.065	140.817
37.787	AP3.10	No intron	AB	2/5/19	179.515	141.728
43.106	AP3.10	No intron	AB	2/5/19	186.939	143.833
43.053	AP3.10	No intron	AB	2/5/19	183.319	140.266
92.552	AP3.10	No intron	AB	2/5/19	232.048	139.496
28.51	AP3.10	No intron	AB	2/5/19	170.03	141.52
37.792	AP3.10	No intron	AB	2/5/19	176.135	138.343
48.44	AP3.10	No intron	AB	2/5/19	189.503	141.063
62.778	AP3.10	No intron	AB	5/14/19	201.583	138.805
52.438	AP3.10	No intron	AB	5/14/19	193.728	141.29
63.195	AP3.10	No intron	AB	5/14/19	204.464	141.269
62.739	AP3.10	No intron	AB	5/14/19	209.037	146.298
72.235	AP3.10	No intron	AB	5/14/19	214.117	141.882
58.054	AP3.10	No intron	AB	5/14/19	198.962	140.908
75.813	AP3.10	No intron	AB	5/14/19	217.638	141.825
82.958	AP3.10	No intron	AB	5/14/19	224.947	141.989
49.29	AP3.10	No intron	AB	5/14/19	190.903	141.613
47.081	AP3.10	No intron	AB	5/14/19	187.65	140.569
54.264	AP3.10	No intron	AB	5/14/19	194.479	140.215
74.193	AP3.10	No intron	AB	5/14/19	215.874	141.681
71.669	AP3.10	No intron	AB	5/14/19	214.083	142.414
61.831	AP3.10	No intron	AB	5/14/19	202.115	140.284
55.138	AP3.10	No intron	AB	5/14/19	197.479	142.341
65.409	AP3.10	No intron	AB	5/14/19	207.885	142.476
52.739	AP3.10	No intron	AB	5/14/19	195.205	142.466

Value	Line	Intron	Scientist	Date	Pharynx Mean	Background Mean
50.807	AP3.10	No intron	AB	6/25/19	182.538	131.731
52.188	AP3.10	No intron	AB	6/25/19	188.871	136.683
52.216	AP3.10	No intron	AB	6/25/19	191.125	138.909
52.538	AP3.10	No intron	AB	6/25/19	188.903	136.365
51.224	AP3.10	No intron	AB	6/25/19	187.159	135.935
36.842	AP3.10	No intron	AB	6/25/19	168.243	131.401
48.528	AP3.10	No intron	AB	6/25/19	182.476	133.948
54.359	AP3.10	No intron	AB	6/25/19	191.309	136.95
47.019	AP3.10	No intron	AB	6/25/19	181.004	133.985
56.462	AP3.10	No intron	AB	6/25/19	192.175	135.713
53.941	AP3.10	No intron	AB	6/25/19	188.638	134.697
56.81	AP3.10	No intron	AB	6/25/19	192.3	135.49
38.305	AP3.10	No intron	AB	6/25/19	172.202	133.897
64.284	AP3.10	No intron	AB	6/25/19	199.941	135.657
58.717	AP3.10	No intron	AB	6/25/19	192.89	134.173
50.633	AP3.10	No intron	AB	6/25/19	186.099	135.466
51.11	AP3.10	No intron	AB	6/25/19	190.927	139.817
55.323	AP3.10	No intron	AB	6/25/19	195.839	140.516
53.6	AP3.10	No intron	AB	6/25/19	191.043	137.443
38.097	AP3.10	No intron	LR	6/6/22	193.819	155.722
21.317	AP3.10	No intron	LR	6/6/22	189.534	168.217
32.482	AP3.10	No intron	LR	6/6/22	192.878	160.396
20.757	AP3.10	No intron	LR	6/6/22	182.295	161.538
68.84	AP3.10	No intron	LR	6/6/22	228.702	159.862
58.409	AP3.10	No intron	AR	5/11/23	209.01	150.601
56.368	AP3.10	No intron	AR	5/11/23	206.119	149.751
55.001	AP3.10	No intron	AR	5/11/23	204.813	149.812
58.008	AP3.10	No intron	AR	5/11/23	208.827	150.819
53.508	AP3.10	No intron	AR	5/11/23	203.064	149.556
108.95	AP3.10	No intron	AR	5/11/23	259.396	150.446
62.767	AP3.10	No intron	AR	5/11/23	212.468	149.701
44.534	AP3.10	No intron	AR	5/11/23	195.506	150.972
110.294	AP3.10	No intron	AR	5/11/23	262.597	152.303
77.533	AP3.10	No intron	AR	5/11/23	226.522	148.989
47.235	AP3.10	No intron	AR	5/11/23	196.913	149.678
60.272	AP3.10	No intron	AR	5/11/23	211.104	150.832

Value	Line	Intron	Scientist	Date	Pharynx Mean	Background Mean
763.297	CB3.H1	unc-54	AB	2/5/19	911.652	148.355
438.065	CB3.H1	unc-54	AB	2/5/19	580.199	142.134
703.985	CB3.H1	unc-54	AB	2/5/19	845.346	141.361
589.132	CB3.H1	unc-54	AB	2/5/19	733.911	144.779
643.268	CB3.H1	unc-54	AB	2/5/19	786.087	142.819
707.372	CB3.H1	unc-54	AB	2/5/19	853.427	146.055
551.706	CB3.H1	unc-54	AB	2/5/19	700.854	149.148
436.218	CB3.H1	unc-54	AB	2/5/19	587.156	150.938
531.428	CB3.H1	unc-54	AB	2/5/19	675.847	144.419
505.414	CB3.H1	unc-54	AB	2/5/19	643.791	138.377
262.755	CB3.H1	unc-54	AB	2/5/19	409.479	146.724
573.278	CB3.H1	unc-54	AB	2/5/19	719.012	145.734
441.858	CB3.H1	unc-54	AB	2/5/19	585.132	143.274
374.529	CB3.H1	unc-54	AB	2/5/19	516.466	141.937
537.417	CB3.H1	unc-54	AB	2/5/19	690.046	152.629
442.614	CB3.H1	unc-54	AB	2/5/19	593.325	150.711
523.071	CB3.H1	unc-54	AB	2/5/19	670.578	147.507
743.626	CB3.H1	unc-54	AB	2/5/19	889.599	145.973
285.811	CB3.H1	unc-54	AB	6/4/19	438.565	152.754
257.543	CB3.H1	unc-54	AB	6/4/19	403.983	146.44
438.013	CB3.H1	unc-54	AB	6/4/19	583.316	145.303
240.992	CB3.H1	unc-54	AB	6/4/19	389.722	148.73
463.2	CB3.H1	unc-54	AB	6/4/19	607.014	143.814
506.969	CB3.H1	unc-54	AB	6/4/19	653.274	146.305
434.98	CB3.H1	unc-54	AB	6/4/19	585.99	151.01
347.588	CB3.H1	unc-54	AB	6/4/19	490.698	143.11
698.564	CB3.H1	unc-54	AB	6/4/19	847.123	148.559
550.738	CB3.H1	unc-54	AB	6/4/19	702.119	151.381
472.93	CB3.H1	unc-54	AB	6/4/19	620.796	147.866
341.934	CB3.H1	unc-54	AB	6/4/19	497.35	155.416
549.939	CB3.H1	unc-54	AB	6/4/19	700.878	150.939
251.902	CB3.H1	unc-54	AB	6/4/19	409.62	157.718
234.668	CB3.H1	unc-54	AB	6/4/19	381.148	146.48

Value	Line	Intron	Scientist	Date	Pharynx Mean	Background Mean
365.662	CB3.H1	unc-54	AB	6/25/19	503.969	138.307
729.964	CB3.H1	unc-54	AB	6/25/19	874.057	144.093
373.907	CB3.H1	unc-54	AB	6/25/19	516.828	142.921
348.082	CB3.H1	unc-54	AB	6/25/19	489.654	141.572
779.615	CB3.H1	unc-54	AB	6/25/19	918.493	138.878
447.415	CB3.H1	unc-54	AB	6/25/19	590.764	143.349
591.453	CB3.H1	unc-54	AB	6/25/19	730.694	139.241
485.902	CB3.H1	unc-54	AB	6/25/19	628.543	142.641
633.861	CB3.H1	unc-54	AB	6/25/19	773.419	139.558
433.916	CB3.H1	unc-54	AB	6/25/19	574.337	140.421
438.308	CB3.H1	unc-54	AB	6/25/19	652.537	214.229
345.157	CB3.H1	unc-54	AB	6/25/19	488.735	143.578
309.38	CB3.H1	unc-54	AB	6/25/19	446.987	137.607
346.282	CB3.H1	unc-54	AB	6/25/19	481.242	134.96
317.284	CB3.H1	unc-54	AB	6/25/19	452.986	135.702
418.873	CB3.H1	unc-54	AR	4/12/23	572.799	153.926
409.781	CB3.H1	unc-54	AR	4/12/23	565.729	155.948
592.805	CB3.H1	unc-54	AR	4/12/23	749.209	156.404
456.082	CB3.H1	unc-54	AR	4/12/23	611.417	155.335
518.191	CB3.H1	unc-54	AR	4/12/23	673.457	155.266
563.299	CB3.H1	unc-54	AR	4/12/23	719.776	156.477
344.745	CB3.H1	unc-54	AR	4/12/23	499.378	154.633
545.883	CB3.H1	unc-54	AR	4/12/23	700.824	154.941
834.508	CB3.H1	unc-54	AR	4/14/23	985.559	151.051
680.683	CB3.H1	unc-54	AR	4/14/23	832.607	151.925
588.180	CB3.H1	unc-54	AR	4/14/23	740.228	152.048

Value	Line	Intron	Scientist	Date	Pharynx Mean	Background Mean
500.94	CR2.13	synthetic	AB	4/10/19	659.601	158.661
530.768	CR2.13	synthetic	AB	4/10/19	685.404	154.636
557.922	CR2.13	synthetic	AB	4/10/19	710.36	152.438
571.628	CR2.13	synthetic	AB	4/10/19	733.754	162.126
455.319	CR2.13	synthetic	AB	4/10/19	604.342	149.023
616.007	CR2.13	synthetic	AB	4/10/19	772.453	156.446
487.211	CR2.13	synthetic	AB	4/10/19	646.867	159.656
527.594	CR2.13	synthetic	AB	4/10/19	677.494	149.9
508.698	CR2.13	synthetic	AB	4/10/19	665.842	157.144
484.468	CR2.13	synthetic	AB	4/10/19	642.444	157.976
499.869	CR2.13	synthetic	AB	4/10/19	653.257	153.388
583.945	CR2.13	synthetic	AB	4/10/19	742.35	158.405
514.297	CR2.13	synthetic	AB	4/10/19	663.33	149.033
423.44	CR2.13	synthetic	AB	4/10/19	575.003	151.563
566.417	CR2.13	synthetic	AB	4/10/19	720.175	153.758
525.887	CR2.13	synthetic	AB	4/10/19	678.861	152.974
511.261	CR2.13	synthetic	AB	4/10/19	666.301	155.04
383.756	CR2.13	synthetic	AB	4/10/19	535.162	151.406
455.19	CR2.13	synthetic	AB	4/10/19	606.228	151.038
396.195	CR2.13	synthetic	AB	4/10/19	544.375	148.18
611.469	CR2.13	synthetic	AB	4/10/19	776.705	165.236
580.881	CR2.13	synthetic	AB	4/23/19	735.533	154.652
614.007	CR2.13	synthetic	AB	4/23/19	776.699	162.692
447.674	CR2.13	synthetic	AB	4/23/19	604.819	157.145
621.368	CR2.13	synthetic	AB	4/23/19	775.68	154.312
535.532	CR2.13	synthetic	AB	4/23/19	701.061	165.529
460.046	CR2.13	synthetic	AB	4/23/19	618.575	158.529
526.836	CR2.13	synthetic	AB	4/23/19	685.403	158.567
611.404	CR2.13	synthetic	AB	4/23/19	768.638	157.234
492.408	CR2.13	synthetic	AB	4/23/19	659.486	167.078
607.736	CR2.13	synthetic	AB	4/23/19	765.456	157.72
351.785	CR2.13	synthetic	AB	4/23/19	517.743	165.958
642.58	CR2.13	synthetic	AB	4/23/19	802.274	159.694
450.944	CR2.13	synthetic	AB	4/23/19	610.411	159.467
458.633	CR2.13	synthetic	AB	4/23/19	623.71	165.077
585.481	CR2.13	synthetic	AB	4/23/19	765.951	180.47
458.962	CR2.13	synthetic	AB	4/23/19	619.368	160.406
312.224	CR2.13	synthetic	AB	4/23/19	555.397	243.173

Value	Line	Intron	Scientist	Date	Pharynx Mean	Background Mean
437.249	CR2.13	synthetic	AB	7/16/19	587.697	150.448
614.713	CR2.13	synthetic	AB	7/16/19	768.172	153.459
500.669	CR2.13	synthetic	AB	7/16/19	658.077	157.408
503.033	CR2.13	synthetic	AB	7/16/19	657.999	154.966
170.02	CR2.13	synthetic	AB	7/16/19	314.057	144.037
459.506	CR2.13	synthetic	AB	7/16/19	612.153	152.647
560.486	CR2.13	synthetic	AB	7/16/19	708.676	148.19
514.812	CR2.13	synthetic	AB	7/16/19	662.931	148.119
653.774	CR2.13	synthetic	AB	7/16/19	807.886	154.112
459.9	CR2.13	synthetic	AB	7/16/19	609.362	149.462
400.79	CR2.13	synthetic	AB	7/16/19	549.345	148.555
473.538	CR2.13	synthetic	AB	7/16/19	622.059	148.521
495.229	CR2.13	synthetic	AB	7/16/19	644.571	149.342
398.423	CR2.13	synthetic	AB	7/16/19	546.646	148.223
632.173	CR2.13	synthetic	AB	7/16/19	782.12	149.947
473.24	CR2.13	synthetic	AB	7/16/19	627.354	154.114

Value	Line	Intron	Scientist	Date	Pharynx Mean	Background Mean
431.634	CR2.4	synthetic	AB	7/30/19	755.396	323.762
486.858	CR2.4	synthetic	AB	7/30/19	640.962	154.104
485.251	CR2.4	synthetic	AB	7/30/19	633.639	148.388
441.893	CR2.4	synthetic	AB	7/30/19	590.769	148.876
313.913	CR2.4	synthetic	AB	7/30/19	465.746	151.833
494.042	CR2.4	synthetic	AB	7/30/19	641.596	147.554
446.758	CR2.4	synthetic	AB	7/30/19	595.676	148.918
636.755	CR2.4	synthetic	AB	7/30/19	785.189	148.434
370.551	CR2.4	synthetic	AB	7/30/19	607.507	236.956
511.981	CR2.4	synthetic	AB	7/30/19	663.34	151.359
475	CR2.4	synthetic	AB	7/30/19	622.144	147.144
573.587	CR2.4	synthetic	AB	7/30/19	724.673	151.086
591.18	CR2.4	synthetic	AB	7/30/19	741.736	150.556
500.176	CR2.4	synthetic	AB	7/30/19	646.152	145.976
438.581	CR2.4	synthetic	AB	7/30/19	583.081	144.5
604.659	CR2.4	synthetic	AB	7/30/19	823.452	218.793
442.04	CR2.4	synthetic	AB	7/30/19	589.262	147.222
526.831	CR2.4	synthetic	AB	7/30/19	674.545	147.714
556.857	CR2.4	synthetic	AB	8/20/19	700.757	143.9
576.685	CR2.4	synthetic	AB	8/20/19	720.472	143.787
355.682	CR2.4	synthetic	AB	8/20/19	497.709	142.027
378.157	CR2.4	synthetic	AB	8/20/19	518.461	140.304
534.32	CR2.4	synthetic	AB	8/20/19	676.936	142.616
699.396	CR2.4	synthetic	AB	8/20/19	848.366	148.97
514.122	CR2.4	synthetic	AB	8/20/19	657.875	143.753
620.016	CR2.4	synthetic	AB	8/20/19	759.63	139.614
590.581	CR2.4	synthetic	AB	8/20/19	733.738	143.157
580.066	CR2.4	synthetic	AB	8/20/19	722.79	142.724
521.259	CR2.4	synthetic	AB	8/20/19	663.576	142.317
459.729	CR2.4	synthetic	AB	8/20/19	605.88	146.151
572.161	CR2.4	synthetic	AB	8/20/19	711.779	139.618
578.254	CR2.4	synthetic	AB	8/20/19	720.54	142.286

Value	Line	Intron	Scientist	Date	Pharynx Mean	Background Mean
630.471	CR2.4	synthetic	AB	8/22/19	778.815	148.344
710.478	CR2.4	synthetic	AB	8/22/19	856.49	146.012
612.393	CR2.4	synthetic	AB	8/22/19	760.012	147.619
490.553	CR2.4	synthetic	AB	8/22/19	637.067	146.514
577.129	CR2.4	synthetic	AB	8/22/19	726.264	149.135
645.844	CR2.4	synthetic	AB	8/22/19	794.107	148.263
620.761	CR2.4	synthetic	AB	8/22/19	769.404	148.643
470.445	CR2.4	synthetic	AB	8/22/19	617.38	146.935
636.946	CR2.4	synthetic	AB	8/22/19	780.766	143.82
605.271	CR2.4	synthetic	AB	8/22/19	753.583	148.312
528.44	CR2.4	synthetic	AB	8/22/19	677.235	148.795
394.03	CR2.4	synthetic	AB	8/22/19	540.448	146.418
535.525	CR2.4	synthetic	AB	8/22/19	680.86	145.335
643.649	CR2.4	synthetic	AB	8/22/19	792.168	148.519
494.977	CR2.4	synthetic	AB	8/22/19	641.649	146.672
618.638	CR2.4	synthetic	AR	4/15/23	770.475	153.54
659.837	CR2.4	synthetic	AR	4/15/23	151.837	715.503
655.956	CR2.4	synthetic	AR	4/15/23	812.821	151.315
518.666	CR2.4	synthetic	AR	4/15/23	152.984	658.892
616.518	CR2.4	synthetic	AR	4/15/23	809.313	151.953
622.71	CR2.4	synthetic	AR	4/15/23	153.357	918.177
564.188	CR2.4	synthetic	AR	4/15/23	670.511	154.792
506.939	CR2.4	synthetic	AR	4/15/23	151.845	860.235
763.385	CR2.4	synthetic	AR	4/15/23	769.199	155.025
705.21	CR2.4	synthetic	AR	4/15/23	152.681	748.27
595.252	CR2.4	synthetic	AR	4/15/23	776.25	153.018

Value	Line	Intron	Scientist	Date	Pharynx Mean	Background Mean
772.34	AR749.10	smu-1	AB	7/16/19	915.878	143.538
566.962	AR749.10	smu-1	AB	7/16/19	716.144	149.182
527.701	AR749.10	smu-1	AB	7/16/19	685.15	157.449
771.929	AR749.10	smu-1	AB	7/16/19	917.256	145.327
473.61	AR749.10	smu-1	AB	7/16/19	622.888	149.278
556.238	AR749.10	smu-1	AB	7/16/19	702.277	146.039
679.194	AR749.10	smu-1	AB	7/16/19	828.786	149.592
729.402	AR749.10	smu-1	AB	7/16/19	885.428	156.026
397.82	AR749.10	smu-1	AB	7/16/19	543.324	145.504
443.95	AR749.10	smu-1	AB	7/16/19	588.905	144.955
750.611	AR749.10	smu-1	AB	7/16/19	898.065	147.454
645.71	AR749.10	smu-1	AB	7/16/19	794.383	148.673
583.537	AR749.10	smu-1	AB	7/16/19	733.087	149.55
474.492	AR749.10	smu-1	AB	7/16/19	624.817	150.325
539.544	AR749.10	smu-1	AB	7/16/19	687.716	148.172
479.77	AR749.10	smu-1	AB	7/16/19	624.677	144.907
486.349	AR749.10	smu-1	AB	7/16/19	629.753	143.404
422.127	AR749.10	smu-1	AB	7/18/19	569.255	147.128
399.012	AR749.10	smu-1	AB	7/18/19	555.567	156.555
265.833	AR749.10	smu-1	AB	7/18/19	414.378	148.545
317.728	AR749.10	smu-1	AB	7/18/19	468.987	151.259
495.628	AR749.10	smu-1	AB	7/18/19	642.371	146.743
260.878	AR749.10	smu-1	AB	7/18/19	407.991	147.113
479.761	AR749.10	smu-1	AB	7/18/19	624.755	144.994
531.515	AR749.10	smu-1	AB	7/18/19	682.507	150.992
557.235	AR749.10	smu-1	AB	7/18/19	709.65	152.415
368.441	AR749.10	smu-1	AB	7/18/19	518.114	149.673
463.623	AR749.10	smu-1	AB	7/18/19	613.003	149.38
403.933	AR749.10	smu-1	AB	7/18/19	555.745	151.812
499.62	AR749.10	smu-1	AB	7/18/19	649.274	149.654
350.54	AR749.10	smu-1	AB	7/18/19	500.273	149.733
428.998	AR749.10	smu-1	AB	7/18/19	577.685	148.687

Value	Line	Intron	Scientist	Date	Pharynx Mean	Background Mean
572.476	AR749.10	smu-1	AB	7/23/19	725.382	152.906
509.912	AR749.10	smu-1	AB	7/23/19	660.283	150.371
389.87	AR749.10	smu-1	AB	7/23/19	544.52	154.65
211.821	AR749.10	smu-1	AB	7/23/19	364.113	152.292
421.993	AR749.10	smu-1	AB	7/23/19	583.267	161.274
254.102	AR749.10	smu-1	AB	7/23/19	406.001	151.899
511.984	AR749.10	smu-1	AB	7/23/19	671.795	159.811
571.438	AR749.10	smu-1	AB	7/23/19	729.433	157.995
479.193	AR749.10	smu-1	AB	7/23/19	635.554	156.361
371.634	AR749.10	smu-1	AB	7/23/19	529.208	157.574
437.031	AR749.10	smu-1	AB	7/23/19	594.043	157.012
421.874	AR749.10	smu-1	AB	7/23/19	573.387	151.513
578.483	AR749.10	smu-1	AB	7/23/19	900.125	321.642
440.506	AR749.10	smu-1	AB	7/23/19	594.885	154.379
466.026	AR749.10	smu-1	AB	7/23/19	622.274	156.248

Value	Line	Intron	Scientist	Date	Pharynx Mean	Background Mean
433.112	AR749.16	smu-1	AB	7/9/19	586.613	153.501
267.613	AR749.16	smu-1	AB	7/9/19	428.598	160.985
489.33	AR749.16	smu-1	AB	7/9/19	645.231	155.901
231.951	AR749.16	smu-1	AB	7/9/19	395.451	163.5
370.828	AR749.16	smu-1	AB	7/9/19	528.914	158.086
302.935	AR749.16	smu-1	AB	7/9/19	463.414	160.479
531.931	AR749.16	smu-1	AB	7/9/19	692.818	160.887
477.65	AR749.16	smu-1	AB	7/9/19	632.189	154.539
427.974	AR749.16	smu-1	AB	7/9/19	585.47	157.496
433.225	AR749.16	smu-1	AB	7/9/19	595.027	161.802
476.064	AR749.16	smu-1	AB	7/9/19	639.726	163.662
418.11	AR749.16	smu-1	AB	7/9/19	577.122	159.012
357.247	AR749.16	smu-1	AB	7/9/19	516.645	159.398
373.189	AR749.16	smu-1	AB	7/9/19	533.811	160.622
438.375	AR749.16	smu-1	AB	7/9/19	594.457	156.082
685.999	AR749.16	smu-1	AB	7/16/19	832.856	146.857
465.634	AR749.16	smu-1	AB	7/16/19	610.418	144.784
562.417	AR749.16	smu-1	AB	7/16/19	710.077	147.66
368.194	AR749.16	smu-1	AB	7/16/19	519.086	150.892
468.415	AR749.16	smu-1	AB	7/16/19	614.464	146.049
479.61	AR749.16	smu-1	AB	7/16/19	624.331	144.721
612.085	AR749.16	smu-1	AB	7/16/19	760.61	148.525
455.654	AR749.16	smu-1	AB	7/16/19	600.448	144.794
741.676	AR749.16	smu-1	AB	7/16/19	896.302	154.626
522.313	AR749.16	smu-1	AB	7/16/19	670.541	148.228
489.058	AR749.16	smu-1	AB	7/16/19	632.559	143.501
437.37	AR749.16	smu-1	AB	7/16/19	581.112	143.742
553.091	AR749.16	smu-1	AB	7/16/19	703.555	150.464
373.745	AR749.16	smu-1	AB	7/16/19	524.113	150.368
615.784	AR749.16	smu-1	AB	7/16/19	762.681	146.897
464.976	AR749.16	smu-1	AB	7/16/19	613.204	148.228

Value	Line	Intron	Scientist	Date	Pharynx Mean	Background Mean
524.003	AR749.16	smu-1	AB	7/18/19	671.972	147.969
353.14	AR749.16	smu-1	AB	7/18/19	506.726	153.586
357.115	AR749.16	smu-1	AB	7/18/19	502.976	145.861
362.353	AR749.16	smu-1	AB	7/18/19	512.848	150.495
281.034	AR749.16	smu-1	AB	7/18/19	427.409	146.375
339.835	AR749.16	smu-1	AB	7/18/19	488.934	149.099
466.599	AR749.16	smu-1	AB	7/18/19	612.388	145.789
420.828	AR749.16	smu-1	AB	7/18/19	568.434	147.606
548.513	AR749.16	smu-1	AB	7/18/19	695.23	146.717
458.764	AR749.16	smu-1	AB	7/18/19	611.867	153.103
522.861	AR749.16	smu-1	AB	7/18/19	676.701	153.84
373.926	AR749.16	smu-1	AB	7/18/19	524.436	150.51
514.863	AR749.16	smu-1	AB	7/18/19	668.615	153.752
390.231	AR749.16	smu-1	AB	7/18/19	538.947	148.716
440.156	AR749.16	smu-1	AB	7/18/19	590.563	150.407
372.361	AR749.16	smu-1	AB	7/18/19	522.87	150.509
488.014	AR749.16	smu-1	AB	7/18/19	638.433	150.419
366.112	AR749.16	smu-1	AB	7/23/19	520.533	154.421
366.033	AR749.16	smu-1	AB	7/23/19	520.875	154.842
159.212	AR749.16	smu-1	AB	7/23/19	312.052	152.84
461.848	AR749.16	smu-1	AB	7/23/19	618.309	156.461
392.912	AR749.16	smu-1	AB	7/23/19	549.985	157.073
424.155	AR749.16	smu-1	AB	7/23/19	579.433	155.278
563.287	AR749.16	smu-1	AB	7/23/19	884.089	320.802
469.752	AR749.16	smu-1	AB	7/23/19	627.579	157.827
359.158	AR749.16	smu-1	AB	7/23/19	512.922	153.764
379.944	AR749.16	smu-1	AB	7/23/19	536.444	156.5
320.139	AR749.16	smu-1	AB	7/23/19	471.224	151.085
367.148	AR749.16	smu-1	AB	7/23/19	520.715	153.567
429.404	AR749.16	smu-1	AB	7/23/19	581.549	152.145
362.447	AR749.16	smu-1	AB	7/23/19	515.194	152.747
424.993	AR749.16	smu-1	AB	7/23/19	576.926	151.933
336.427	AR749.16	smu-1	AB	7/23/19	487.813	151.386

Value	Line	Intron	Scientist	Date	Pharynx Mean	Background Mean
593.357	AB2.13	rpl-14	AB	7/2/19	750.077	156.72
456.164	AB2.13	rpl-14	AB	7/2/19	615.042	158.878
446.597	AB2.13	rpl-14	AB	7/2/19	601.82	155.223
616.182	AB2.13	rpl-14	AB	7/2/19	769.302	153.12
581.219	AB2.13	rpl-14	AB	7/2/19	733.564	152.345
724.479	AB2.13	rpl-14	AB	7/2/19	883.199	158.72
512.925	AB2.13	rpl-14	AB	7/2/19	665.213	152.288
323.182	AB2.13	rpl-14	AB	7/2/19	476.417	153.235
457.389	AB2.13	rpl-14	AB	7/2/19	610.079	152.69
753.783	AB2.13	rpl-14	AB	7/2/19	911.589	157.806
504.171	AB2.13	rpl-14	AB	7/2/19	662.016	157.845
461.874	AB2.13	rpl-14	AB	7/2/19	611.708	149.834
546.666	AB2.13	rpl-14	AB	7/2/19	703.564	156.898
737.555	AB2.13	rpl-14	AB	7/2/19	893.611	156.056
725.335	AB2.13	rpl-14	AB	7/16/19	874.149	148.814
684.514	AB2.13	rpl-14	AB	7/16/19	832.526	148.012
557.029	AB2.13	rpl-14	AB	7/16/19	707.225	150.196
489.117	AB2.13	rpl-14	AB	7/16/19	637.746	148.629
617.37	AB2.13	rpl-14	AB	7/16/19	774.696	157.326
651.016	AB2.13	rpl-14	AB	7/16/19	793.636	142.62
470.653	AB2.13	rpl-14	AB	7/16/19	616.904	146.251
568.492	AB2.13	rpl-14	AB	7/16/19	715.582	147.09
630.517	AB2.13	rpl-14	AB	7/16/19	780.264	149.747
391.313	AB2.13	rpl-14	AB	7/16/19	540.411	149.098
528.594	AB2.13	rpl-14	AB	7/16/19	691.477	162.883
655.076	AB2.13	rpl-14	AB	7/16/19	805.475	150.399
394.496	AB2.13	rpl-14	AB	7/16/19	542.911	148.415
605.21	AB2.13	rpl-14	AB	7/16/19	750.377	145.167
396.89	AB2.13	rpl-14	AB	7/16/19	544.594	147.704
741.411	AB2.13	rpl-14	AB	7/16/19	886.421	145.01
678.77	AB2.13	rpl-14	AB	7/16/19	824.596	145.826
712.07	AB2.13	rpl-14	AB	7/16/19	862.319	150.249

Value	Line	Intron	Scientist	Date	Pharynx Mean	Background Mean
502.408	AB2.13	rpl-14	AB	7/18/19	654.439	152.031
700.715	AB2.13	rpl-14	AB	7/18/19	850.099	149.384
449.648	AB2.13	rpl-14	AB	7/18/19	605.305	155.657
607.603	AB2.13	rpl-14	AB	7/18/19	834.821	227.218
329.554	AB2.13	rpl-14	AB	7/18/19	486.36	156.806
504.852	AB2.13	rpl-14	AB	7/18/19	659.517	154.665
324.738	AB2.13	rpl-14	AB	7/18/19	475.819	151.081
516.682	AB2.13	rpl-14	AB	7/18/19	669.686	153.004
635.393	AB2.13	rpl-14	AB	7/18/19	789.777	154.384
620.757	AB2.13	rpl-14	AB	7/18/19	773.796	153.039
593.739	AB2.13	rpl-14	AB	7/18/19	743.486	149.747
554.014	AB2.13	rpl-14	AB	7/18/19	703.417	149.403
425.78	AB2.13	rpl-14	AB	7/18/19	575.801	150.021
271.619	AB2.13	rpl-14	AB	7/18/19	421.196	149.577
410.776	AB2.13	rpl-14	AB	7/18/19	559.702	148.926
436.119	AB2.13	rpl-14	AB	7/18/19	586.225	150.106

Value	Line	Intron	Scientist	Date	Pharynx Mean	Background Mean
420.289	AB2.14	rpl-14	AB	7/16/19	562.145	141.856
554.172	AB2.14	rpl-14	AB	7/16/19	698.18	144.008
480.478	AB2.14	rpl-14	AB	7/16/19	629.523	149.045
427.409	AB2.14	rpl-14	AB	7/16/19	580.075	152.666
543.093	AB2.14	rpl-14	AB	7/16/19	689.102	146.009
480.469	AB2.14	rpl-14	AB	7/16/19	623.007	142.538
314.201	AB2.14	rpl-14	AB	7/16/19	458.639	144.438
310.736	AB2.14	rpl-14	AB	7/16/19	457.04	146.304
421.084	AB2.14	rpl-14	AB	7/16/19	571.947	150.863
432.69	AB2.14	rpl-14	AB	7/16/19	584.893	152.203
407.752	AB2.14	rpl-14	AB	7/16/19	552.133	144.381
578.345	AB2.14	rpl-14	AB	7/16/19	721.299	142.954
359.132	AB2.14	rpl-14	AB	7/16/19	514.913	155.781
436.452	AB2.14	rpl-14	AB	7/16/19	582.313	145.861
590.922	AB2.14	rpl-14	AB	7/16/19	736.662	145.74
350.535	AB2.14	rpl-14	AB	7/18/19	505.372	154.837
541.485	AB2.14	rpl-14	AB	7/18/19	696.958	155.473
585.778	AB2.14	rpl-14	AB	7/18/19	762.691	176.913
577.741	AB2.14	rpl-14	AB	7/18/19	728.413	150.672
572.876	AB2.14	rpl-14	AB	7/18/19	724.842	151.966
577.361	AB2.14	rpl-14	AB	7/18/19	731.338	153.977
495.181	AB2.14	rpl-14	AB	7/18/19	648.64	153.459
506.546	AB2.14	rpl-14	AB	7/18/19	666.953	160.407
500.304	AB2.14	rpl-14	AB	7/18/19	653.745	153.441
375.775	AB2.14	rpl-14	AB	7/18/19	590.733	214.958
541.155	AB2.14	rpl-14	AB	7/18/19	692.375	151.22
359.846	AB2.14	rpl-14	AB	7/18/19	513.934	154.088
561.165	AB2.14	rpl-14	AB	7/18/19	716.64	155.475
585.746	AB2.14	rpl-14	AB	7/18/19	740.414	154.668
337.453	AB2.14	rpl-14	AB	7/18/19	489.716	152.263
466.376	AB2.14	rpl-14	AB	7/18/19	615.795	149.419
286.744	AB2.14	rpl-14	AB	7/18/19	436.971	150.227
480.641	AB2.14	rpl-14	AB	7/18/19	634.086	153.445

Value	Line	Intron	Scientist	Date	Pharynx Mean	Background Mean
1199.907	AB2.14	rpl-14	AR	4/27/23	1358.859	158.952
1099.118	AB2.14	rpl-14	AR	4/27/23	1256.289	157.171
812.413	AB2.14	rpl-14	AR	4/27/23	969.937	157.524
945.336	AB2.14	rpl-14	AR	4/27/23	1103.061	157.725
964.526	AB2.14	rpl-14	AR	4/27/23	1122.073	157.547
865.611	AB2.14	rpl-14	AR	4/27/23	1022.367	156.756
627.651	AB2.14	rpl-14	AR	4/27/23	784.216	156.565
715.685	AB2.14	rpl-14	AR	4/27/23	876.576	160.891
809.204	AB2.14	rpl-14	AR	5/11/23	983.48	174.276
495.808	AB2.14	rpl-14	AR	5/11/23	656.253	160.445
768.133	AB2.14	rpl-14	AR	5/11/23	922.185	154.052
842.5	AB2.14	rpl-14	AR	5/11/23	996.344	153.844
699.727	AB2.14	rpl-14	AR	5/11/23	854.67	154.943
775.381	AB2.14	rpl-14	AR	5/11/23	930.146	154.765
715.075	AB2.14	rpl-14	AR	5/11/23	870.091	155.016
549.042	AB2.14	rpl-14	AR	5/11/23	701.996	152.954

Value	Line	Intron	Scientist	Date	Pharynx Mean	Background Mean
917.895	AR726.3	lbp-6	AR	4/20/23	1072.187	154.292
1532.426	AR726.3	lbp-6	AR	4/20/23	1690.761	158.335
1302.516	AR726.3	lbp-6	AR	4/20/23	1458.616	156.1
940.42	AR726.3	lbp-6	AR	4/20/23	1096.462	156.042
1309.687	AR726.3	lbp-6	AR	4/20/23	1467.136	157.449
1347.763	AR726.3	lbp-6	AR	4/20/23	1505.976	158.213
1369.648	AR726.3	lbp-6	AR	4/20/23	1526.867	157.219
1112.094	AR726.3	lbp-6	AR	4/20/23	1268.741	156.647
1312.035	AR726.3	lbp-6	AR	4/20/23	1468.021	155.986
575.051	AR726.3	lbp-6	AR	4/27/23	732.778	157.727
662.885	AR726.3	lbp-6	AR	4/27/23	818.055	155.17
1036.367	AR726.3	lbp-6	AR	4/27/23	1195.547	159.18
652.03	AR726.3	lbp-6	AR	4/27/23	808.369	156.339
394.649	AR726.3	lbp-6	AR	4/27/23	551.715	157.066
496.147	AR726.3	lbp-6	AR	4/27/23	650.824	154.677
936.343	AR726.3	lbp-6	AR	4/27/23	1093.658	157.315
875.896	AR726.3	lbp-6	AR	4/27/23	1031.239	155.343
454.822	AR726.3	lbp-6	AR	4/27/23	609.173	154.351
500.818	AR726.3	lbp-6	AR	4/27/23	655.453	154.635
478.865	AR726.3	lbp-6	AR	4/27/23	633.856	154.991
719.813	AR726.3	lbp-6	AR	4/27/23	875.354	155.541
453.594	AR726.3	lbp-6	AR	4/27/23	609.077	155.483
606.592	AR726.3	lbp-6	AR	4/27/23	761.001	154.409
252.028	AR726.3	lbp-6	AR	4/27/23	406.08	154.052
445.646	AR726.3	lbp-6	AR	4/27/23	599.925	154.279
446.584	AR726.3	lbp-6	AR	4/27/23	601.641	155.057
554.772	AR726.3	lbp-6	AR	4/27/23	710.482	155.71
438.636	AR726.3	lbp-6	AR	5/4/23	591.807	153.171
460.186	AR726.3	lbp-6	AR	5/4/23	614.416	154.23
317.828	AR726.3	lbp-6	AR	5/4/23	469.109	151.281
419.487	AR726.3	lbp-6	AR	5/4/23	572.461	152.974
433.628	AR726.3	lbp-6	AR	5/4/23	586.319	152.691
893.57	AR726.3	lbp-6	AR	5/4/23	1048.237	154.667
522.742	AR726.3	lbp-6	AR	5/4/23	674.819	152.077
530.607	AR726.3	lbp-6	AR	5/4/23	685.886	155.279
396.154	AR726.3	lbp-6	AR	5/4/23	553.092	156.938
488.98	AR726.3	lbp-6	AR	5/4/23	643.776	154.796
499.267	AR726.3	lbp-6	AR	5/4/23	653.796	154.529
479.806	AR726.3	lbp-6	AR	5/4/23	634.603	154.797
432.898	AR726.3	lbp-6	AR	5/4/23	586.014	153.116
477.508	AR726.3	lbp-6	AR	5/4/23	631.543	154.035

Value	Line	Intron	Scientist	Date	Pharynx Mean	Background Mean
368.034	AR726.6	lbp-6	AB	2/5/19	568.883	200.849
519.05	AR726.6	lbp-6	AB	2/5/19	716.772	197.722
341.859	AR726.6	lbp-6	AB	2/5/19	548.344	206.485
453.291	AR726.6	lbp-6	AB	2/5/19	598.243	144.952
427.617	AR726.6	lbp-6	AB	2/5/19	572.499	144.882
255.698	AR726.6	lbp-6	AB	2/5/19	402.035	146.337
375.852	AR726.6	lbp-6	AB	2/5/19	521.018	145.166
352.701	AR726.6	lbp-6	AB	2/5/19	495.168	142.467
585.523	AR726.6	lbp-6	AB	2/5/19	739.738	154.215
561.754	AR726.6	lbp-6	AB	2/5/19	708.394	146.64
378.727	AR726.6	lbp-6	AB	2/5/19	522.877	144.15
467.473	AR726.6	lbp-6	AB	2/5/19	612.302	144.829
567.073	AR726.6	lbp-6	AB	2/5/19	718.093	151.02
455.756	AR726.6	lbp-6	AB	2/5/19	600.838	145.082
527.431	AR726.6	lbp-6	AB	2/5/19	676.553	149.122
389.91	AR726.6	lbp-6	AB	2/5/19	534.52	144.61
341.304	AR726.6	lbp-6	AB	2/5/19	487.93	146.626
555.079	AR726.6	lbp-6	AB	2/5/19	701.177	146.098
485.001	AR726.6	lbp-6	AB	2/5/19	628.94	143.939
480.855	AR726.6	lbp-6	AB	2/5/19	628.746	147.891
575.551	AR726.6	lbp-6	AB	2/5/19	724.505	148.954
337.94	AR726.6	lbp-6	AB	2/5/19	483.716	145.776
436.858	AR726.6	lbp-6	AB	5/14/19	583.216	146.358
321.687	AR726.6	lbp-6	AB	5/14/19	470.768	149.081
425.854	AR726.6	lbp-6	AB	5/14/19	572.332	146.478
654.886	AR726.6	lbp-6	AB	5/14/19	800.221	145.335
727.399	AR726.6	lbp-6	AB	5/14/19	881.397	153.998
701.259	AR726.6	lbp-6	AB	5/14/19	857.461	156.202
528.08	AR726.6	lbp-6	AB	5/14/19	685.073	156.993
468.174	AR726.6	lbp-6	AB	5/14/19	620.631	152.457
430.122	AR726.6	lbp-6	AB	5/14/19	576.288	146.166
499.218	AR726.6	lbp-6	AB	5/14/19	644.652	145.434
681.117	AR726.6	lbp-6	AB	5/14/19	825.782	144.665
680.076	AR726.6	lbp-6	AB	5/14/19	828.468	148.392
353.983	AR726.6	lbp-6	AB	5/14/19	506.671	152.688
462.917	AR726.6	lbp-6	AB	5/14/19	608.495	145.578

Value	Line	Intron	Scientist	Date	Pharynx Mean	Background Mean
244.616	AR726.6	lbp-6	AB	6/11/19	396.159	151.543
297.188	AR726.6	lbp-6	AB	6/11/19	447.077	149.889
272.037	AR726.6	lbp-6	AB	6/11/19	424.741	152.704
428.236	AR726.6	lbp-6	AB	6/11/19	584.953	156.717
276.162	AR726.6	lbp-6	AB	6/11/19	429.986	153.824
499.958	AR726.6	lbp-6	AB	6/11/19	658.581	158.623
239.852	AR726.6	lbp-6	AB	6/11/19	387.805	147.953
427.82	AR726.6	lbp-6	AB	6/11/19	581.229	153.409
593.251	AR726.6	lbp-6	AB	6/11/19	753.72	160.469
385.944	AR726.6	lbp-6	AB	6/11/19	541.355	155.411
238.376	AR726.6	lbp-6	AB	6/11/19	390.396	152.02
382.291	AR726.6	lbp-6	AB	6/11/19	541.598	159.307
580.854	AR726.6	lbp-6	AB	6/11/19	737.468	156.614
477.502	AR726.6	lbp-6	AB	6/11/19	637.889	160.387
392.046	AR726.6	lbp-6	AB	6/11/19	546.759	154.713
472.189	AR726.6	lbp-6	AB	6/11/19	625.414	153.225
307.135	AR726.6	lbp-6	LR	6/6/22	491.069	183.934
384.459	AR726.6	lbp-6	LR	6/6/22	565.128	180.669
404.951	AR726.6	lbp-6	LR	6/6/22	581.065	176.114
382.995	AR726.6	lbp-6	LR	6/6/22	550.938	167.943
365.429	AR726.6	lbp-6	LR	6/6/22	538.687	173.258
399.318	AR726.6	lbp-6	LR	6/6/22	577.98	178.662
497.778	AR726.6	lbp-6	LR	6/6/22	670.064	172.286
422.496	AR726.6	lbp-6	LR	6/6/22	592.346	169.85

Value	Line	Intron	Scientist	Date	Pharynx Mean	Background Mean
273.865	AR726.11	lbp-6	LR	6/6/22	443.349	169.484
440.712	AR726.11	lbp-6	LR	6/6/22	616.212	175.5
470.554	AR726.11	lbp-6	LR	6/6/22	642.912	172.358
375.148	AR726.11	lbp-6	LR	6/6/22	547.707	172.559
441.602	AR726.11	lbp-6	LR	6/6/22	613.877	172.275
388.738	AR726.11	lbp-6	LR	6/6/22	561.026	172.288
398.968	AR726.11	lbp-6	LR	6/6/22	571.929	172.961
333.114	AR726.11	lbp-6	LR	6/6/22	539.848	206.734
307.616	AR726.11	lbp-6	LR	6/6/22	482.242	174.626
458.822	AR726.11	lbp-6	LR	6/6/22	633.669	174.847
468.387	AR726.11	lbp-6	AR	4/12/23	623.478	155.092
445.666	AR726.11	lbp-6	AR	4/12/23	599.856	154.190
506.886	AR726.11	lbp-6	AR	4/12/23	663.370	156.484
775.780	AR726.11	lbp-6	AR	4/12/23	930.304	154.525
595.851	AR726.11	lbp-6	AR	4/12/23	752.047	156.196
522.503	AR726.11	lbp-6	AR	4/12/23	677.748	155.246
835.016	AR726.11	lbp-6	AR	4/14/23	987.264	152.248
538.996	AR726.11	lbp-6	AR	4/14/23	689.989	150.993
701.193	AR726.11	lbp-6	AR	4/14/23	851.353	150.160
758.547	AR726.11	lbp-6	AR	5/2/23	916.697	158.15
564.931	AR726.11	lbp-6	AR	5/2/23	721.386	156.455
462.198	AR726.11	lbp-6	AR	5/2/23	618.847	156.649
584.361	AR726.11	lbp-6	AR	5/2/23	741.658	157.297
539.8	AR726.11	lbp-6	AR	5/2/23	693.805	154.005
462.339	AR726.11	lbp-6	AR	5/2/23	615.876	153.537
419.663	AR726.11	lbp-6	AR	5/2/23	573.453	153.79
588.853	AR726.11	lbp-6	AR	5/2/23	748.128	159.275
635.702	AR726.11	lbp-6	AR	5/2/23	791.285	155.583
471.384	AR726.11	lbp-6	AR	5/2/23	627.184	155.8
580.135	AR726.11	lbp-6	AR	5/2/23	735.679	155.544
590.38	AR726.11	lbp-6	AR	5/2/23	747.158	156.778
522.525	AR726.11	lbp-6	AR	5/2/23	678.804	156.279
784.239	AR726.11	lbp-6	AR	5/2/23	942.672	158.433

Value	Line	Intron	Scientist	Date	Pharynx Mean	Background Mean
1026.778	AR726.11	lbp-6	AR	5/9/23	1182.795	156.017
951.795	AR726.11	lbp-6	AR	5/9/23	1106.633	154.838
835.635	AR726.11	lbp-6	AR	5/9/23	991.278	155.643
589.081	AR726.11	lbp-6	AR	5/9/23	745.33	156.249
773.454	AR726.11	lbp-6	AR	5/9/23	929.429	155.975
702.973	AR726.11	lbp-6	AR	5/9/23	861.108	158.135
683.724	AR726.11	lbp-6	AR	5/9/23	838.493	154.769
859.325	AR726.11	lbp-6	AR	5/9/23	1013.859	154.534
766.327	AR726.11	lbp-6	AR	5/9/23	921.306	154.979
640.141	AR726.11	lbp-6	AR	5/9/23	794.915	154.774
638.725	AR726.11	lbp-6	AR	5/9/23	793.674	154.949
844.419	AR726.11	lbp-6	AR	5/9/23	997.393	152.974

Value	Line	Intron	Scientist	Date	Pharynx Mean	Background Mean
802.294	CB1.6	gpx-1	AR	4/20/23	952.774	150.48
1063.731	CB1.6	gpx-1	AR	4/20/23	1218.906	155.175
661.499	CB1.6	gpx-1	AR	4/20/23	815.541	154.042
365.231	CB1.6	gpx-1	AR	4/20/23	517.226	151.995
1000.153	CB1.6	gpx-1	AR	4/20/23	1153.552	153.399
907.867	CB1.6	gpx-1	AR	4/20/23	1060.766	152.899
884.127	CB1.6	gpx-1	AR	4/20/23	1036.931	152.804
749.329	CB1.6	gpx-1	AR	4/20/23	900.27	150.941
681.905	CB1.6	gpx-1	AR	4/20/23	832.629	150.724
818.034	CB1.6	gpx-1	AR	4/20/23	971.003	152.969
840.598	CB1.6	gpx-1	AR	4/27/23	997.698	157.1
571.125	CB1.6	gpx-1	AR	4/27/23	727.479	156.354
835.903	CB1.6	gpx-1	AR	4/27/23	991.101	155.198
530.381	CB1.6	gpx-1	AR	4/27/23	684.289	153.908
706.871	CB1.6	gpx-1	AR	4/27/23	863.997	157.126
611.054	CB1.6	gpx-1	AR	4/27/23	767.275	156.221
799.036	CB1.6	gpx-1	AR	4/27/23	956.072	157.036
669.401	CB1.6	gpx-1	AR	4/27/23	825.591	156.19
894.727	CB1.6	gpx-1	AR	4/27/23	1051.314	156.587
735.149	CB1.6	gpx-1	AR	4/27/23	890.58	155.431
736.408	CB1.6	gpx-1	AR	4/27/23	892.23	155.822
719.452	CB1.6	gpx-1	AR	4/27/23	878.067	158.615
668.666	CB1.6	gpx-1	AR	4/27/23	826.54	157.874
514.819	CB1.6	gpx-1	AR	5/4/23	672.219	157.4
980.869	CB1.6	gpx-1	AR	5/4/23	1140.377	159.508
551.534	CB1.6	gpx-1	AR	5/4/23	712.467	160.933
580.269	CB1.6	gpx-1	AR	5/4/23	736.354	156.085
395.634	CB1.6	gpx-1	AR	5/4/23	551.566	155.932
464.739	CB1.6	gpx-1	AR	5/4/23	621.003	156.264
529.968	CB1.6	gpx-1	AR	5/4/23	687.438	157.47
608.572	CB1.6	gpx-1	AR	5/4/23	766.233	157.661
301.655	CB1.6	gpx-1	AR	5/4/23	456.556	154.901
543.071	CB1.6	gpx-1	AR	5/4/23	697.835	154.764
340.24	CB1.6	gpx-1	AR	5/4/23	496.813	156.573
521.385	CB1.6	gpx-1	AR	5/4/23	677.743	156.358
568.651	CB1.6	gpx-1	AR	5/4/23	723.543	154.892
427.83	CB1.6	gpx-1	AR	5/4/23	584.06	156.23
262.704	CB1.6	gpx-1	AR	5/4/23	416.985	154.281

Value	Line	Intron	Scientist	Date	Pharynx Mean	Background Mean
874.44	CB1.6	gpx-1	AR	5/9/23	1030.542	156.102
548.885	CB1.6	gpx-1	AR	5/9/23	705.149	156.264
736.917	CB1.6	gpx-1	AR	5/9/23	892.596	155.679
484.882	CB1.6	gpx-1	AR	5/9/23	640.418	155.536
502.895	CB1.6	gpx-1	AR	5/9/23	658.089	155.194
641.565	CB1.6	gpx-1	AR	5/9/23	810.381	168.816
561.143	CB1.6	gpx-1	AR	5/9/23	716.71	155.567
668.47	CB1.6	gpx-1	AR	5/9/23	825.73	157.26
732.267	CB1.6	gpx-1	AR	5/9/23	888.707	156.44

Value	Line	Intron	Scientist	Date	Pharynx Mean	Background Mean
483.545	CB1.9	gpx-1	AB	4/10/19	627.529	143.984
760.276	CB1.9	gpx-1	AB	4/10/19	911.496	151.22
553.703	CB1.9	gpx-1	AB	4/10/19	698.188	144.485
455.906	CB1.9	gpx-1	AB	4/10/19	611.767	155.861
558.689	CB1.9	gpx-1	AB	4/10/19	830.07	271.381
436.582	CB1.9	gpx-1	AB	4/10/19	626.476	189.894
436.967	CB1.9	gpx-1	AB	4/10/19	585.438	148.471
396.89	CB1.9	gpx-1	AB	4/10/19	549.798	152.908
656.47	CB1.9	gpx-1	AB	4/10/19	808.95	152.48
454.335	CB1.9	gpx-1	AB	4/10/19	607.943	153.608
508.435	CB1.9	gpx-1	AB	4/10/19	662.664	154.229
397.918	CB1.9	gpx-1	AB	4/10/19	550.919	153.001
520.212	CB1.9	gpx-1	AB	4/10/19	673.059	152.847
687.962	CB1.9	gpx-1	AB	4/10/19	844.092	156.13
625.485	CB1.9	gpx-1	AB	4/10/19	791.126	165.641
725.052	CB1.9	gpx-1	AB	4/10/19	884.054	159.002
497.726	CB1.9	gpx-1	AB	4/10/19	643.648	145.922
549.723	CB1.9	gpx-1	AB	4/10/19	705.704	155.981
406.965	CB1.9	gpx-1	AB	4/23/19	553.027	146.062
373.719	CB1.9	gpx-1	AB	4/23/19	527.783	154.064
515.685	CB1.9	gpx-1	AB	4/23/19	668.661	152.976
457.045	CB1.9	gpx-1	AB	4/23/19	609.102	152.057
455.446	CB1.9	gpx-1	AB	4/23/19	606.49	151.044
428.489	CB1.9	gpx-1	AB	4/23/19	581.811	153.322
272.829	CB1.9	gpx-1	AB	4/23/19	425.587	152.758
376.781	CB1.9	gpx-1	AB	4/23/19	526.376	149.595
465.375	CB1.9	gpx-1	AB	4/23/19	616.376	151.001
447.531	CB1.9	gpx-1	AB	4/23/19	596.41	148.879
522.728	CB1.9	gpx-1	AB	4/23/19	673.408	150.68
511.037	CB1.9	gpx-1	AB	4/23/19	664.097	153.06
515.084	CB1.9	gpx-1	AB	4/23/19	665.704	150.62
465.625	CB1.9	gpx-1	AB	4/23/19	616.388	150.763
498.431	CB1.9	gpx-1	AB	4/23/19	646.31	147.879
455.806	CB1.9	gpx-1	AB	4/23/19	606.941	151.135
499.92	CB1.9	gpx-1	AB	4/23/19	650.932	151.012
500.324	CB1.9	gpx-1	AB	4/23/19	650.872	150.548
555.315	CB1.9	gpx-1	AB	4/23/19	705.646	150.331
529.1	CB1.9	gpx-1	AB	4/23/19	683.174	154.074
507.22	CB1.9	gpx-1	AB	4/23/19	660.72	153.5
486.827	CB1.9	gpx-1	AB	4/23/19	637.071	150.244
669.746	CB1.9	gpx-1	AB	4/23/19	823.152	153.406

Value	Line	Intron	Scientist	Date	Pharynx Mean	Background Mean
439.819	CB1.9	gpx-1	AB	6/4/19	582.236	142.417
381.206	CB1.9	gpx-1	AB	6/4/19	526.311	145.105
337.046	CB1.9	gpx-1	AB	6/4/19	482.444	145.398
406.97	CB1.9	gpx-1	AB	6/4/19	551.739	144.769
478.964	CB1.9	gpx-1	AB	6/4/19	628.105	149.141
363.732	CB1.9	gpx-1	AB	6/4/19	508.456	144.724
439.602	CB1.9	gpx-1	AB	6/4/19	581.446	141.844
328.513	CB1.9	gpx-1	AB	6/4/19	469.038	140.525
297.127	CB1.9	gpx-1	AB	6/4/19	444.035	146.908
386.667	CB1.9	gpx-1	AB	6/4/19	531.861	145.194
457.841	CB1.9	gpx-1	AB	6/4/19	606.079	148.238
562.352	CB1.9	gpx-1	AB	6/4/19	705.556	143.204
405.085	CB1.9	gpx-1	AB	6/4/19	552.762	147.677
463.395	CB1.9	gpx-1	AB	6/4/19	605.975	142.58
241.509	CB1.9	gpx-1	AB	6/4/19	388.583	147.074
453.373	CB1.9	gpx-1	AB	6/4/19	601.34	147.967
403.4	CB1.9	gpx-1	AB	6/4/19	547.713	144.313

Value	Line	Intron	Scientist	Date	Pharynx Mean	Background Mean
666.16	AP1.1	mec-8	AB	7/30/19	813.444	147.284
709.651	AP1.1	mec-8	AB	7/30/19	860.742	151.091
565.914	AP1.1	mec-8	AB	7/30/19	717.475	151.561
542.062	AP1.1	mec-8	AB	7/30/19	695.226	153.164
499.704	AP1.1	mec-8	AB	7/30/19	658.026	158.322
582.162	AP1.1	mec-8	AB	7/30/19	728.911	146.749
637.231	AP1.1	mec-8	AB	7/30/19	785.025	147.794
719.278	AP1.1	mec-8	AB	7/30/19	876.574	157.296
594.624	AP1.1	mec-8	AB	7/30/19	746.704	152.08
400.462	AP1.1	mec-8	AB	7/30/19	549.225	148.763
607.186	AP1.1	mec-8	AB	7/30/19	762.943	155.757
724.346	AP1.1	mec-8	AB	7/30/19	883.659	159.313
357.378	AP1.1	mec-8	AB	7/30/19	514.863	157.485
615.389	AP1.1	mec-8	AB	7/30/19	765.165	149.776
505.18	AP1.1	mec-8	AB	7/30/19	654.827	149.647
730.474	AP1.1	mec-8	AB	7/30/19	882.065	151.591
515.955	AP1.1	mec-8	AB	7/30/19	668	152.045
469.758	AP1.1	mec-8	AB	8/20/19	615.496	145.738
356.514	AP1.1	mec-8	AB	8/20/19	509.207	152.693
454.164	AP1.1	mec-8	AB	8/20/19	600.066	145.902
472.208	AP1.1	mec-8	AB	8/20/19	619.482	147.274
504.015	AP1.1	mec-8	AB	8/20/19	648.318	144.303
549.008	AP1.1	mec-8	AB	8/20/19	702.384	153.376
471.62	AP1.1	mec-8	AB	8/20/19	619.275	147.655
431.297	AP1.1	mec-8	AB	8/20/19	575.449	144.152
624.75	AP1.1	mec-8	AB	8/20/19	771.261	146.511
505.09	AP1.1	mec-8	AB	8/20/19	651.162	146.072
454.485	AP1.1	mec-8	AB	8/20/19	600.649	146.164
413.808	AP1.1	mec-8	AB	8/20/19	557.83	144.022
676.767	AP1.1	mec-8	AB	8/20/19	825.981	149.214
677.195	AP1.1	mec-8	AB	8/20/19	825.777	148.582

Value	Line	Intron	Scientist	Date	Pharynx Mean	Background Mean
815.285	AP1.1	mec-8	AB	8/22/19	960.11	144.825
651.915	AP1.1	mec-8	AB	8/22/19	804.302	152.387
704.083	AP1.1	mec-8	AB	8/22/19	850.167	146.084
696.733	AP1.1	mec-8	AB	8/22/19	844.922	148.189
709.038	AP1.1	mec-8	AB	8/22/19	854.564	145.526
684.724	AP1.1	mec-8	AB	8/22/19	839.927	155.203
655.474	AP1.1	mec-8	AB	8/22/19	797.16	141.686
649.088	AP1.1	mec-8	AB	8/22/19	792.625	143.537
612.997	AP1.1	mec-8	AB	8/22/19	761.437	148.44
455.17	AP1.1	mec-8	AB	8/22/19	600.92	145.75
542.222	AP1.1	mec-8	AB	8/22/19	691.749	149.527
562.725	AP1.1	mec-8	AB	8/22/19	708.989	146.264
488.574	AP1.1	mec-8	AB	8/22/19	641.936	153.362
623.129	AP1.1	mec-8	AB	8/22/19	766.586	143.457
724.688	AP1.1	mec-8	AB	8/22/19	872.345	147.657
589.22	AP1.1	mec-8	AB	8/22/19	735.107	145.887
519.33	AP1.1	mec-8	AB	8/22/19	665.581	146.251
450.443	AP1.1	mec-8	AB	8/22/19	593.46	143.017

Value	Line	Intron	Scientist	Date	Pharynx Mean	Background Mean
1026.047	AP1.12	mec-8	AR	4/20/23	1184.017	157.97
879.917	AP1.12	mec-8	AR	4/20/23	1037.732	157.815
754.208	AP1.12	mec-8	AR	4/20/23	909.238	155.03
596.635	AP1.12	mec-8	AR	4/20/23	752.003	155.368
1151.326	AP1.12	mec-8	AR	4/20/23	1309.777	158.451
816.263	AP1.12	mec-8	AR	4/20/23	971.943	155.68
299.91	AP1.12	mec-8	AR	4/20/23	458.243	158.333
447.717	AP1.12	mec-8	AR	4/27/23	667.976	220.259
492.803	AP1.12	mec-8	AR	4/27/23	650.021	157.218
420.81	AP1.12	mec-8	AR	4/27/23	573.212	152.402
310.091	AP1.12	mec-8	AR	4/27/23	462.457	152.366
294.474	AP1.12	mec-8	AR	4/27/23	448.32	153.846
359.415	AP1.12	mec-8	AR	4/27/23	512.436	153.021
317.48	AP1.12	mec-8	AR	4/27/23	469.68	152.2
281.443	AP1.12	mec-8	AR	4/27/23	434.127	152.684
310.78	AP1.12	mec-8	AR	4/27/23	464.513	153.733
356.535	AP1.12	mec-8	AR	4/27/23	508.975	152.44
372.118	AP1.12	mec-8	AR	4/27/23	526.422	154.304
383.246	AP1.12	mec-8	AR	4/27/23	535.661	152.415
339.727	AP1.12	mec-8	AR	4/27/23	496.787	157.06
352.717	AP1.12	mec-8	AR	5/2/23	510.611	157.894
252.232	AP1.12	mec-8	AR	5/2/23	407.022	154.79
477.457	AP1.12	mec-8	AR	5/2/23	636.212	158.755
254.28	AP1.12	mec-8	AR	5/2/23	406.649	152.369
490.494	AP1.12	mec-8	AR	5/2/23	646.488	155.994
371.31	AP1.12	mec-8	AR	5/2/23	527.887	156.577
393.01	AP1.12	mec-8	AR	5/2/23	548.323	155.313
451.614	AP1.12	mec-8	AR	5/2/23	608.169	156.555
488.335	AP1.12	mec-8	AR	5/2/23	643.372	155.037
437.436	AP1.12	mec-8	AR	5/2/23	595.504	158.068
473.521	AP1.12	mec-8	AR	5/2/23	629.638	156.117
359.526	AP1.12	mec-8	AR	5/2/23	513.272	153.746
336.796	AP1.12	mec-8	AR	5/2/23	494.18	157.384
418.295	AP1.12	mec-8	AR	5/2/23	574.768	156.473
421.999	AP1.12	mec-8	AR	5/2/23	579.236	157.237
503.846	AP1.12	mec-8	AR	5/2/23	660.957	157.111
490.654	AP1.12	mec-8	AR	5/2/23	647.017	156.363
861.411	AP1.12	mec-8	AR	5/9/23	1019.338	157.927
888.301	AP1.12	mec-8	AR	5/9/23	1045.427	157.126
904.713	AP1.12	mec-8	AR	5/9/23	1061.053	156.34
939.083	AP1.12	mec-8	AR	5/9/23	1097.625	158.542
679.925	AP1.12	mec-8	AR	5/9/23	836.526	156.601
635.688	AP1.12	mec-8	AR	5/9/23	790.506	154.818

Value	Line	Intron	Scientist	Date	Pharynx Mean	Background Mean
341.389	HT4.5	daao-1	AB	4/16/19	482.337	140.948
776.582	HT4.5	daao-1	AB	4/16/19	929.482	152.9
691.341	HT4.5	daao-1	AB	4/16/19	852.878	161.537
726.082	HT4.5	daao-1	AB	4/16/19	889.86	163.778
554.693	HT4.5	daao-1	AB	4/16/19	709.842	155.149
456.559	HT4.5	daao-1	AB	4/16/19	606.148	149.589
543.613	HT4.5	daao-1	AB	4/16/19	697.163	153.55
434.407	HT4.5	daao-1	AB	4/16/19	582.727	148.32
456.116	HT4.5	daao-1	AB	4/16/19	613.8	157.684
540.709	HT4.5	daao-1	AB	4/16/19	697.139	156.43
482.907	HT4.5	daao-1	AB	4/16/19	639.33	156.423
476.981	HT4.5	daao-1	AB	4/16/19	632.989	156.008
628.475	HT4.5	daao-1	AB	4/16/19	785.515	157.04
490.802	HT4.5	daao-1	AB	4/16/19	643.33	152.528
671.691	HT4.5	daao-1	AB	4/16/19	827.331	155.64
617.318	HT4.5	daao-1	AB	4/16/19	770.84	153.522
548.414	HT4.5	daao-1	AB	4/16/19	703.832	155.418
413.674	HT4.5	daao-1	AB	4/16/19	570.216	156.542
359.678	HT4.5	daao-1	AB	4/16/19	517	157.322
727.842	HT4.5	daao-1	AB	4/16/19	892.018	164.176
517.075	HT4.5	daao-1	AB	4/30/19	678.616	161.541
427.017	HT4.5	daao-1	AB	4/30/19	586.959	159.942
484.324	HT4.5	daao-1	AB	4/30/19	646.343	162.019
521.927	HT4.5	daao-1	AB	4/30/19	683.639	161.712
613.675	HT4.5	daao-1	AB	4/30/19	959.556	345.881
627.834	HT4.5	daao-1	AB	4/30/19	797.726	169.892
472.287	HT4.5	daao-1	AB	4/30/19	637.879	165.592
358.04	HT4.5	daao-1	AB	4/30/19	522.435	164.395
504.845	HT4.5	daao-1	AB	4/30/19	660.424	155.579
423.02	HT4.5	daao-1	AB	4/30/19	587.104	164.084
530.15	HT4.5	daao-1	AB	4/30/19	685.74	155.59
470.086	HT4.5	daao-1	AB	4/30/19	629.901	159.815
516.164	HT4.5	daao-1	AB	4/30/19	672.076	155.912
406.69	HT4.5	daao-1	AB	4/30/19	569.994	163.304
405.588	HT4.5	daao-1	AB	4/30/19	565.62	160.032
431.371	HT4.5	daao-1	AB	4/30/19	589.343	157.972
411.815	HT4.5	daao-1	AB	4/30/19	570.281	158.466
395.392	HT4.5	daao-1	AB	4/30/19	551.213	155.821
409.449	HT4.5	daao-1	AB	4/30/19	563.014	153.565

Value	Line	Intron	Scientist	Date	Pharynx Mean	Background Mean
358.025	HT4.5	daao-1	AB	7/2/19	514.45	156.425
457.688	HT4.5	daao-1	AB	7/2/19	618.672	160.984
440.396	HT4.5	daao-1	AB	7/2/19	598.875	158.479
472.549	HT4.5	daao-1	AB	7/2/19	628.33	155.781
504.27	HT4.5	daao-1	AB	7/2/19	658.949	154.679
389.563	HT4.5	daao-1	AB	7/2/19	548.883	159.32
361.983	HT4.5	daao-1	AB	7/2/19	517.363	155.38
309.355	HT4.5	daao-1	AB	7/2/19	464.648	155.293
201.909	HT4.5	daao-1	AB	7/2/19	356.834	154.925
334.203	HT4.5	daao-1	AB	7/2/19	493.337	159.134
282.211	HT4.5	daao-1	AB	7/2/19	447.154	164.943
675.455	HT4.5	daao-1	AB	7/2/19	831.309	155.854
272.699	HT4.5	daao-1	AB	7/2/19	426.272	153.573
516.476	HT4.5	daao-1	AB	7/2/19	668.111	151.635
426.476	HT4.5	daao-1	AB	7/2/19	581.659	155.183
624.333	HT4.5	daao-1	AB	7/2/19	808.75	184.417
407.562	HT4.5	daao-1	AB	7/2/19	560.562	153
538.194	HT4.5	daao-1	AB	7/2/19	702.817	164.623
504.233	HT4.5	daao-1	AB	7/2/19	670.946	166.713
438.287	HT4.5	daao-1	AB	7/2/19	592.703	154.416
670.583	HT4.5	daao-1	AR	4/25/23	826.154	155.571
1076.976	HT4.5	daao-1	AR	4/25/23	1231.847	154.871
772.491	HT4.5	daao-1	AR	4/25/23	927.966	155.475
660.406	HT4.5	daao-1	AR	4/25/23	814.608	154.202
615.599	HT4.5	daao-1	AR	4/25/23	770.815	155.216
518.68	HT4.5	daao-1	AR	4/25/23	673.134	154.454
383.115	HT4.5	daao-1	AR	4/25/23	536.158	153.043
705.254	HT4.5	daao-1	AR	4/25/23	859.935	154.681
704.963	HT4.5	daao-1	AR	4/25/23	861.142	156.179
440.409	HT4.5	daao-1	AR	4/25/23	594.132	153.723
641.217	HT4.5	daao-1	AR	4/25/23	796.739	155.522
509.608	HT4.5	daao-1	AR	4/25/23	663.046	153.438

Value	Line	Intron	Scientist	Date	Pharynx Mean	Background Mean
752.579	HT4.6	daao-1	AB	4/16/19	905.195	152.616
409.19	HT4.6	daao-1	AB	4/16/19	558.786	149.596
515.955	HT4.6	daao-1	AB	4/16/19	666.114	150.159
614.014	HT4.6	daao-1	AB	4/16/19	767.738	153.724
395.809	HT4.6	daao-1	AB	4/16/19	554.379	158.57
550.637	HT4.6	daao-1	AB	4/16/19	698.918	148.281
457.319	HT4.6	daao-1	AB	4/16/19	605.87	148.551
579.643	HT4.6	daao-1	AB	4/16/19	733.719	154.076
485.586	HT4.6	daao-1	AB	4/16/19	629.567	143.981
453.014	HT4.6	daao-1	AB	4/16/19	596.604	143.59
513.089	HT4.6	daao-1	AB	4/16/19	663.227	150.138
340.224	HT4.6	daao-1	AB	4/16/19	486.127	145.903
466.516	HT4.6	daao-1	AB	4/16/19	617.186	150.67
503.356	HT4.6	daao-1	AB	4/16/19	660.465	157.109
554.586	HT4.6	daao-1	AB	4/16/19	706.182	151.596
494.331	HT4.6	daao-1	AB	4/16/19	648.973	154.642
494.496	HT4.6	daao-1	AB	4/16/19	647.966	153.47
491.21	HT4.6	daao-1	AB	4/16/19	647.195	155.985
394.977	HT4.6	daao-1	AB	4/30/19	557.197	162.22
242.245	HT4.6	daao-1	AB	4/30/19	400.656	158.411
345.014	HT4.6	daao-1	AB	4/30/19	509.812	164.798
476.012	HT4.6	daao-1	AB	4/30/19	640.58	164.568
479.82	HT4.6	daao-1	AB	4/30/19	642.057	162.237
348.621	HT4.6	daao-1	AB	4/30/19	507.415	158.794
236.219	HT4.6	daao-1	AB	4/30/19	386.673	150.454
393.867	HT4.6	daao-1	AB	4/30/19	549.669	155.802
232.618	HT4.6	daao-1	AB	4/30/19	383.417	150.799
371.706	HT4.6	daao-1	AB	4/30/19	528.127	156.421
386.663	HT4.6	daao-1	AB	4/30/19	546.729	160.066
526.902	HT4.6	daao-1	AB	4/30/19	682.043	155.141
317.809	HT4.6	daao-1	AB	4/30/19	468.997	151.188
243.596	HT4.6	daao-1	AB	4/30/19	394.597	151.001
371.108	HT4.6	daao-1	AB	4/30/19	526.545	155.437

Value	Line	Intron	Scientist	Date	Pharynx Mean	Background Mean
340.104	HT4.6	daao-1	AB	7/2/19	492.119	152.015
302.19	HT4.6	daao-1	AB	7/2/19	454.676	152.486
428.397	HT4.6	daao-1	AB	7/2/19	582.621	154.224
405.726	HT4.6	daao-1	AB	7/2/19	564.564	158.838
201.472	HT4.6	daao-1	AB	7/2/19	357.063	155.591
369.725	HT4.6	daao-1	AB	7/2/19	524.714	154.989
385.111	HT4.6	daao-1	AB	7/2/19	551.445	166.334
365.142	HT4.6	daao-1	AB	7/2/19	528.872	163.73
216.218	HT4.6	daao-1	AB	7/2/19	371.733	155.515
605.877	HT4.6	daao-1	AB	7/2/19	765.564	159.687
329.69	HT4.6	daao-1	AB	7/2/19	478.066	148.376
544.814	HT4.6	daao-1	AB	7/2/19	701.587	156.773
429.615	HT4.6	daao-1	AB	7/2/19	587.978	158.363
332.597	HT4.6	daao-1	AB	7/2/19	487.375	154.778
504.661	HT4.6	daao-1	AR	4/25/23	659.746	155.085
633.669	HT4.6	daao-1	AR	4/25/23	788.447	154.778
634.108	HT4.6	daao-1	AR	4/25/23	788.551	154.443
593.262	HT4.6	daao-1	AR	4/25/23	748.237	154.975
390.35	HT4.6	daao-1	AR	4/25/23	549.781	159.431
649.024	HT4.6	daao-1	AR	4/25/23	809.268	160.244
379.117	HT4.6	daao-1	AR	4/25/23	531.918	152.801
566.201	HT4.6	daao-1	AR	4/25/23	720.193	153.992
403.441	HT4.6	daao-1	AR	4/25/23	560.456	157.015
229.268	HT4.6	daao-1	AR	4/25/23	388.77	159.502
769.735	HT4.6	daao-1	AR	4/25/23	926.279	156.544

Value	Line	Intron	Scientist	Date	Pharynx Mean	Background Mean
670.06	AR727.7	gbb-1	AB	2/5/19	820.295	150.235
633.049	AR727.7	gbb-1	AB	2/5/19	784.324	151.275
614.353	AR727.7	gbb-1	AB	2/5/19	764.71	150.357
482.453	AR727.7	gbb-1	AB	2/5/19	623.437	140.984
569.097	AR727.7	gbb-1	AB	2/5/19	719.995	150.898
579.889	AR727.7	gbb-1	AB	2/5/19	732.646	152.757
674.476	AR727.7	gbb-1	AB	2/5/19	824.546	150.07
699.474	AR727.7	gbb-1	AB	2/5/19	851.077	151.603
667.544	AR727.7	gbb-1	AB	2/5/19	819.23	151.686
456.359	AR727.7	gbb-1	AB	2/5/19	606.302	149.943
441.029	AR727.7	gbb-1	AB	2/5/19	587.287	146.258
481.29	AR727.7	gbb-1	AB	2/5/19	628.22	146.93
587.065	AR727.7	gbb-1	AB	2/5/19	738.447	151.382
458.019	AR727.7	gbb-1	AB	2/5/19	608.292	150.273
658.491	AR727.7	gbb-1	AB	2/5/19	805.31	146.819
477.804	AR727.7	gbb-1	AB	2/5/19	619.859	142.055
615.944	AR727.7	gbb-1	AB	2/5/19	764.909	148.965
518.832	AR727.7	gbb-1	AB	2/5/19	665.741	146.909
762.773	AR727.7	gbb-1	AB	2/12/19	915.526	152.753
797.419	AR727.7	gbb-1	AB	2/12/19	951.962	154.543
970.679	AR727.7	gbb-1	AB	2/12/19	1122.727	152.048
772.969	AR727.7	gbb-1	AB	2/12/19	927.954	154.985
758.248	AR727.7	gbb-1	AB	2/12/19	920.915	162.667
806.967	AR727.7	gbb-1	AB	2/12/19	952.929	145.962
928.263	AR727.7	gbb-1	AB	2/12/19	1078.858	150.595
807.166	AR727.7	gbb-1	AB	2/12/19	963.787	156.621
882.899	AR727.7	gbb-1	AB	2/12/19	1033.132	150.233
824.281	AR727.7	gbb-1	AB	2/12/19	979.559	155.278
744.197	AR727.7	gbb-1	AB	2/12/19	901.924	157.727
708.74	AR727.7	gbb-1	AB	2/12/19	866.584	157.844
940.664	AR727.7	gbb-1	AB	2/12/19	1088.111	147.447
694.475	AR727.7	gbb-1	AB	2/12/19	851.015	156.54
907.096	AR727.7	gbb-1	AB	2/12/19	1063.863	156.767

Value	Line	Intron	Scientist	Date	Pharynx Mean	Background Mean
474.444	AR727.7	gbb-1	AB	6/11/19	629.283	154.839
396.801	AR727.7	gbb-1	AB	6/11/19	548.701	151.9
551.189	AR727.7	gbb-1	AB	6/11/19	706.311	155.122
601.869	AR727.7	gbb-1	AB	6/11/19	754.722	152.853
503.417	AR727.7	gbb-1	AB	6/11/19	700.197	196.78
482.655	AR727.7	gbb-1	AB	6/11/19	631.542	148.887
458.072	AR727.7	gbb-1	AB	6/11/19	607.473	149.401
489.327	AR727.7	gbb-1	AB	6/11/19	644.714	155.387
520.413	AR727.7	gbb-1	AB	6/11/19	671.805	151.392
472.142	AR727.7	gbb-1	AB	6/11/19	625.077	152.935
516.856	AR727.7	gbb-1	AB	6/11/19	667.605	150.749
462.038	AR727.7	gbb-1	AB	6/11/19	642.669	180.631
562.396	AR727.7	gbb-1	AR	4/25/23	719.321	156.925
487.601	AR727.7	gbb-1	AR	4/25/23	642.542	154.941
391.324	AR727.7	gbb-1	AR	4/25/23	551.787	160.463
515.885	AR727.7	gbb-1	AR	4/25/23	672.471	156.586
439.262	AR727.7	gbb-1	AR	4/25/23	611.482	172.22
510.183	AR727.7	gbb-1	AR	4/25/23	679.489	169.306
587.508	AR727.7	gbb-1	AR	4/25/23	742.029	154.521
448.22	AR727.7	gbb-1	AR	4/25/23	598.977	150.757
366.384	AR727.7	gbb-1	AR	4/25/23	524.238	157.854
463.348	AR727.7	gbb-1	AR	4/25/23	626.282	162.934
446.889	AR727.7	gbb-1	AR	4/25/23	599.71	152.821
600.412	AR727.7	gbb-1	AR	4/25/23	755.832	155.42
769.265	AR727.7	gbb-1	AR	4/25/23	925.208	155.943
508.988	AR727.7	gbb-1	AR	4/25/23	662.159	153.171
383.525	AR727.7	gbb-1	AR	4/25/23	542.24	158.715

Value	Line	Intron	Scientist	Date	Pharynx Mean	Background Mean
732.397	AR727.14	gbb-1	AB	2/12/19	884.6	152.203
626.264	AR727.14	gbb-1	AB	2/12/19	775.807	149.543
631.703	AR727.14	gbb-1	AB	2/12/19	778.892	147.189
862.327	AR727.14	gbb-1	AB	2/12/19	1024.346	162.019
567.592	AR727.14	gbb-1	AB	2/12/19	722.901	155.309
697.034	AR727.14	gbb-1	AB	2/12/19	848.424	151.39
611.942	AR727.14	gbb-1	AB	2/12/19	778.402	166.46
700.465	AR727.14	gbb-1	AB	2/12/19	855.941	155.476
466.795	AR727.14	gbb-1	AB	2/12/19	613.069	146.274
512.068	AR727.14	gbb-1	AB	2/12/19	658.342	146.274
439.348	AR727.14	gbb-1	AB	2/12/19	591.823	152.475
542.604	AR727.14	gbb-1	AB	2/12/19	711.182	168.578
616.622	AR727.14	gbb-1	AB	2/12/19	778.351	161.729
371.431	AR727.14	gbb-1	AB	2/12/19	531.191	159.76
558.321	AR727.14	gbb-1	AB	2/12/19	712.162	153.841
557.671	AR727.14	gbb-1	AB	2/12/19	712.621	154.95
710.733	AR727.14	gbb-1	AB	2/12/19	871.954	161.221
594.993	AR727.14	gbb-1	AB	2/12/19	747.142	152.149
677.659	AR727.14	gbb-1	AB	2/12/19	827.965	150.306
817.224	AR727.14	gbb-1	AB	2/12/19	972.893	155.669
367.431	AR727.14	gbb-1	AB	6/11/19	521.391	153.96
372.416	AR727.14	gbb-1	AB	6/11/19	529.672	157.256
498.748	AR727.14	gbb-1	AB	6/11/19	653.888	155.14
265.563	AR727.14	gbb-1	AB	6/11/19	431.359	165.796
583.835	AR727.14	gbb-1	AB	6/11/19	756.53	172.695
265.271	AR727.14	gbb-1	AB	6/11/19	427.144	161.873
547.221	AR727.14	gbb-1	AB	6/11/19	698.211	150.99
558.822	AR727.14	gbb-1	AB	6/11/19	715.567	156.745
547.747	AR727.14	gbb-1	AB	6/11/19	704.131	156.384
476.025	AR727.14	gbb-1	AB	6/11/19	636.891	160.866
327.075	AR727.14	gbb-1	AB	6/11/19	477.838	150.763
336.395	AR727.14	gbb-1	AB	6/11/19	492.525	156.13
481.325	AR727.14	gbb-1	AB	6/11/19	634.553	153.228
451.353	AR727.14	gbb-1	AR	4/25/23	605.793	154.44
229.631	AR727.14	gbb-1	AR	4/25/23	381.396	151.765
423.016	AR727.14	gbb-1	AR	4/25/23	578.528	155.512
402.286	AR727.14	gbb-1	AR	4/25/23	555.532	153.246
377.019	AR727.14	gbb-1	AR	4/25/23	532.379	155.36
447.289	AR727.14	gbb-1	AR	4/25/23	601.924	154.635
282.474	AR727.14	gbb-1	AR	4/25/23	436.37	153.896
374.261	AR727.14	gbb-1	AR	4/25/23	528.851	154.59
731.741	AR727.14	gbb-1	AR	4/25/23	888.167	156.426
652.693	AR727.14	gbb-1	AR	4/25/23	809.309	156.616
687.749	AR727.14	gbb-1	AR	4/25/23	847.059	159.31

Value	Line	Intron	Scientist	Date	Pharynx Mean	Background Mean
542.132	AR728.7	cutl-4	AB	7/30/19	683.205	141.073
505.855	AR728.7	cutl-4	AB	7/30/19	652.019	146.164
561.888	AR728.7	cutl-4	AB	7/30/19	708.121	146.233
625.581	AR728.7	cutl-4	AB	7/30/19	776.252	150.671
534.446	AR728.7	cutl-4	AB	7/30/19	680.742	146.296
611.992	AR728.7	cutl-4	AB	7/30/19	760.892	148.9
442.675	AR728.7	cutl-4	AB	7/30/19	597.115	154.44
545.29	AR728.7	cutl-4	AB	7/30/19	691.348	146.058
493.64	AR728.7	cutl-4	AB	7/30/19	645.556	151.916
519.757	AR728.7	cutl-4	AB	7/30/19	666.832	147.075
531.449	AR728.7	cutl-4	AB	7/30/19	683.642	152.193
504.86	AR728.7	cutl-4	AB	7/30/19	655.475	150.615
395.264	AR728.7	cutl-4	AB	7/30/19	540.478	145.214
501.674	AR728.7	cutl-4	AB	7/30/19	652.198	150.524
579.981	AR728.7	cutl-4	AB	7/30/19	728.159	148.178
486.112	AR728.7	cutl-4	AB	7/30/19	639.288	153.176
422.025	AR728.7	cutl-4	AB	7/30/19	567.727	145.702
495.6	AR728.7	cutl-4	AB	7/30/19	646.007	150.407
519.736	AR728.7	cutl-4	AB	8/20/19	665.657	145.921
677.493	AR728.7	cutl-4	AB	8/20/19	829.143	151.65
439.713	AR728.7	cutl-4	AB	8/20/19	582.482	142.769
657.164	AR728.7	cutl-4	AB	8/20/19	800.435	143.271
404.015	AR728.7	cutl-4	AB	8/20/19	556.073	152.058
398.753	AR728.7	cutl-4	AB	8/20/19	542.084	143.331
511.974	AR728.7	cutl-4	AB	8/20/19	655.154	143.18
437.706	AR728.7	cutl-4	AB	8/20/19	578.626	140.92
535.006	AR728.7	cutl-4	AB	8/20/19	680.302	145.296
701.373	AR728.7	cutl-4	AB	8/20/19	849.65	148.277
360.62	AR728.7	cutl-4	AB	8/20/19	502.544	141.924
431.696	AR728.7	cutl-4	AB	8/20/19	578.607	146.911
640.905	AR728.7	cutl-4	AB	8/20/19	785.459	144.554
513.366	AR728.7	cutl-4	AB	8/20/19	653.453	140.087

Value	Line	Intron	Scientist	Date	Pharynx Mean	Background Mean
516.008	AR728.7	cutl-4	AB	8/22/19	663.674	147.666
435.81	AR728.7	cutl-4	AB	8/22/19	576.237	140.427
628.657	AR728.7	cutl-4	AB	8/22/19	775.94	147.283
560.927	AR728.7	cutl-4	AB	8/22/19	703.823	142.896
457.39	AR728.7	cutl-4	AB	8/22/19	600.839	143.449
564.003	AR728.7	cutl-4	AB	8/22/19	706.618	142.615
680.72	AR728.7	cutl-4	AB	8/22/19	823.667	142.947
601.87	AR728.7	cutl-4	AB	8/22/19	745.903	144.033
472.702	AR728.7	cutl-4	AB	8/22/19	619.325	146.623
554.319	AR728.7	cutl-4	AB	8/22/19	701.023	146.704
595.02	AR728.7	cutl-4	AB	8/22/19	742.789	147.769
656.821	AR728.7	cutl-4	AB	8/22/19	805.377	148.556
534.712	AR728.7	cutl-4	AB	8/22/19	678.802	144.09
513.761	AR728.7	cutl-4	AB	8/22/19	659.83	146.069
632.696	AR728.7	cutl-4	AB	8/22/19	776.92	144.224
640.299	AR728.7	cutl-4	AB	8/22/19	786.001	145.702
466.217	AR728.7	cutl-4	LR	6/10/22	639.378	173.161
419.891	AR728.7	cutl-4	LR	6/10/22	578.536	158.645
454.161	AR728.7	cutl-4	LR	6/10/22	617.438	163.277
260.902	AR728.7	cutl-4	LR	6/10/22	421.537	160.635
307.559	AR728.7	cutl-4	LR	6/10/22	471.248	163.689
605.367	AR728.7	cutl-4	LR	6/10/22	779.158	173.791
400.056	AR728.7	cutl-4	LR	6/10/22	564.749	164.693

Value	Line	Intron	Scientist	Date	Pharynx Mean	Background Mean
400.831	AR728.10	cutl-4	AR	4/19/23	554.697	153.866
471.434	AR728.10	cutl-4	AR	4/19/23	625.471	154.037
393.982	AR728.10	cutl-4	AR	4/19/23	558.674	164.692
578.798	AR728.10	cutl-4	AR	4/19/23	733.412	154.614
515.58	AR728.10	cutl-4	AR	4/19/23	669.802	154.222
587.776	AR728.10	cutl-4	AR	4/19/23	742.994	155.218
529.929	AR728.10	cutl-4	AR	4/19/23	685.69	155.761
632.575	AR728.10	cutl-4	AR	4/19/23	788.507	155.932
545.808	AR728.10	cutl-4	AR	4/19/23	700.554	154.746
369.62	AR728.10	cutl-4	AR	4/19/23	521.441	151.821
371.493	AR728.10	cutl-4	AR	5/2/23	524.484	152.991
492.959	AR728.10	cutl-4	AR	5/2/23	644.316	151.357
603.471	AR728.10	cutl-4	AR	5/2/23	756.73	153.259
417.429	AR728.10	cutl-4	AR	5/2/23	568.24	150.811
253.022	AR728.10	cutl-4	AR	5/2/23	404.546	151.524
415.74	AR728.10	cutl-4	AR	5/2/23	567.672	151.932
238.17	AR728.10	cutl-4	AR	5/2/23	389.673	151.503
352.514	AR728.10	cutl-4	AR	5/2/23	504.507	151.993
397.657	AR728.10	cutl-4	AR	5/2/23	550.896	153.239
513.128	AR728.10	cutl-4	AR	5/2/23	665.457	152.329
441.59	AR728.10	cutl-4	AR	5/2/23	592.183	150.593
327.239	AR728.10	cutl-4	AR	5/2/23	479.013	151.774
355.814	AR728.10	cutl-4	AR	5/2/23	508.129	152.315
438.757	AR728.10	cutl-4	AR	5/2/23	590.896	152.139
445.106	AR728.10	cutl-4	AR	5/2/23	596.397	151.291
742.391	AR728.10	cutl-4	AR	5/4/23	897.584	155.193
529.226	AR728.10	cutl-4	AR	5/4/23	684.195	154.969
627.318	AR728.10	cutl-4	AR	5/4/23	783.1	155.782
346.665	AR728.10	cutl-4	AR	5/4/23	498.515	151.85
405.11	AR728.10	cutl-4	AR	5/4/23	554.025	148.915
407.566	AR728.10	cutl-4	AR	5/4/23	558.133	150.567
373.795	AR728.10	cutl-4	AR	5/4/23	525.341	151.546
513.486	AR728.10	cutl-4	AR	5/4/23	666.223	152.737
250.984	AR728.10	cutl-4	AR	5/4/23	402.596	151.612
375.579	AR728.10	cutl-4	AR	5/4/23	527.773	152.194
437.637	AR728.10	cutl-4	AR	5/4/23	586.831	149.194
293.353	AR728.10	cutl-4	AR	5/4/23	440.88	147.527
364.675	AR728.10	cutl-4	AR	5/4/23	513.517	148.842

Value	Line	Intron	Scientist	Date	Pharynx Mean	Background Mean
695.169	AR728.10	cutl-4	AR	5/9/23	848.02	152.851
734.711	AR728.10	cutl-4	AR	5/9/23	888.949	154.238
654.639	AR728.10	cutl-4	AR	5/9/23	808.754	154.115
658.122	AR728.10	cutl-4	AR	5/9/23	811.84	153.718
841.883	AR728.10	cutl-4	AR	5/9/23	995.893	154.01
573.887	AR728.10	cutl-4	AR	5/9/23	725.377	151.49
637.772	AR728.10	cutl-4	AR	5/9/23	790.609	152.837
638.836	AR728.10	cutl-4	AR	5/9/23	790.417	151.581

Table S6. RNA gel blot raw data

Gel N114

	Quantfification method										
	Rectangle				Grid				Peak		Loading control
Line	Band count	Band area	Bkg count	Bkg area	Band count	Band area	Bkg count	Bkg area	Peak area	Peak height	EtBr ATBV
N2	660,624	2,040	562,059	1,725	707,996	2,170	717,066	2,232	-	-	1,155,168
AP3.6	819,493	2,496	472,117	1,525	716,176	2,170	683,947	2,232	1,415	62	3,716,352
CB3.H1	1,168,596	2,460	517,144	1,769	1,040,694	2,124	629,957	2,124	10,369	501	1,727,088
CR2.4	1,431,104	2,436	356,671	1,311	1,410,764	2,408	677,605	2,464	17,544	824	3,561,504
AR749.10	996,208	2,400	339,291	1,298	999,581	2,419	644,905	2,478	9,150	413	2,285,760
AB2.14	785,910	2,331	424,881	1,620	830,746	2,501	677,752	2,562	3,768	197	924,048
AR726.3	1,434,475	2,700	466,876	1,770	1,321,559	2,352	620,857	2,352	17,769	764	3,525,888
CB1.9	906,316	2,666	446,698	1,708	911,669	2,684	707,865	2,745	4,892	200	1,197,024
AP1.12	1,080,547	3,520	434,547	1,800	914,056	2,904	714,248	2,970	4,836	175	1,010,496
HT4.5	1,231,842	3,477	360,407	1,464	952,958	2,464	614,558	2,520	8,801	313	1,542,144
AR727.7	862,353	2,576	469,541	1,870	768,845	2,240	565,500	2,240	5,258	215	705,888
AR728.7	1,407,157	2,392	422,054	1,632	1,389,200	2,397	632,555	2,448	18,650	763	2,328,144

Gel N115

	Quantfification method										
	Rectangle				Grid				Peak		Loading control
Line	Band count	Band area	Bkg count	Bkg area	Band count	Band area	Bkg count	Bkg area	Peak area	Peak height	EtBr ATBV
AP3.10	769,109	2,772	531,871	2,025	578,107	2,070	547,620	2,070	646	50	990,624
CB3.10	1,249,820	2,419	320,462	1,239	1,152,320	2,160	582,512	2,220	13,217	681	626,304
CR2.13	1,162,694	2,700	363,551	1,425	1,008,391	2,220	575,535	2,220	10,583	476	1,069,488
AR749.16	1,610,998	2,784	359,731	1,368	1,478,084	2,394	648,305	2,451	19,421	917	3,551,184
AB2.13	1,752,578	2,950	417,322	1,593	1,636,731	2,610	692,101	2,610	20,755	882	2,575,824
AR726.3	1,755,048	2,891	374,156	1,475	1,618,987	2,508	657,312	2,565	20,838	961	3,890,880
AR726.6	920,263	2,666	414,402	1,624	807,054	2,280	586,016	2,280	4,894	231	1,104,144
AR726.11	960,200	2,508	475,618	1,829	895,755	2,280	587,915	2,280	7,031	342	1,371,648
CB1.6	1,583,924	2,760	380,653	1,482	1,634,644	2,891	745,052	2,891	20,246	879	3,234,672
AP1.1	1,128,213	2,655	357,330	1,400	1,070,930	2,436	637,933	2,494	10,346	469	1,736,400
HT4.6	961,578	2,726	375,438	1,540	827,593	2,240	551,132	2,240	7,098	314	998,496
AR727.14	921,372	2,806	322,084	1,392	714,703	2,040	487,856	2,040	5,797	260	631,152
AR728.10	1,300,212	2,940	384,147	1,595	1,151,102	2,432	637,114	2,496	12,593	609	1,502,160

Gel N116 Shown in Fig 3

Quantfification method

	Rectangle				Grid				Peak		Loading control
Line	Band count	Band area	Bkg count	Bkg area	Band count	Band area	Bkg count	Bkg area	Peak area	Peak height	EtBr ATBV
N2	747,265	1,855	574,791	1,458	737,355	1,836	715,824	1,836	1,510	76	2,647,274
AP3.6	1,019,240	2,520	637,416	1,767	972,716	2,400	870,643	2,400	2,936	40	2,574,019
AP3.10	955,256	2,310	580,374	1,650	969,600	2,365	863,834	2,420	3,164	122	2,837,443
CB3.H1	2,076,309	2,596	630,739	1,798	1,917,088	2,242	825,260	2,301	26,032	996	2,507,085
CB3.10	2,798,547	2,784	592,295	1,680	2,552,178	2,296	831,754	2,296	40,343	1,506	2,316,965
CR2.4	1,810,788	2,610	514,745	1,624	1,643,134	2,184	729,963	2,240	19,882	750	2,837,002
CR2.13	1,784,439	2,565	510,053	1,682	1,780,765	2,464	786,458	2,520	22,271	728	2,321,718
AR749.10	1,534,958	2,793	477,760	1,475	1,258,264	2,072	713,244	2,128	13,961	433	2,083,578
AR749.16	1,750,165	3,180	452,002	1,380	1,647,837	2,832	951,348	2,891	14,811	467	2,047,367
AB2.13	2,138,145	3,906	588,827	1,798	1,817,213	3,024	1,030,733	3,087	15,629	492	2,000,719
AB2.14	2,088,238	3,355	550,390	1,674	1,882,348	2,773	951,945	2,832	20,438	637	2,028,306
AR726.3	2,073,468	3,248	552,933	1,593	1,891,916	2,805	974,976	2,805	22,546	653	1,830,052
AR726.6	1,813,281	2,808	500,675	1,484	1,715,282	2,520	853,610	2,520	21,295	644	1,628,270
AR726.11	2,332,423	2,842	592,473	1,710	2,217,687	2,565	893,104	2,565	32,206	1,051	1,794,184

Gel N117 Shown in Fig 3

Quantfification method

	Rectangle				Grid				Peak		Loading control
Line	Band count	Band area	Bkg count	Bkg area	Band count	Band area	Bkg count	Bkg area	Peak area	Peak height	EtBr ATBV
N2	494,005	1,862	264,278	1,056	490,946	1,833	463,929	1,833	1,749	76	3,060,491
AP3.10	698,775	2,255	276,015	1,100	714,336	2,337	588,362	2,337	2,682	99	2,818,039
CB1.6	1,757,561	2,736	243,559	952	1,768,624	2,820	699,871	2,820	24,402	742	2,918,097
CB1.9	1,819,368	3,180	284,159	1,121	1,767,178	3,060	746,037	3,060	21,702	672	2,857,974
AP1.1	1,945,542	3,233	290,453	1,160	1,947,224	3,276	805,631	3,339	22,901	656	2,693,726
AP1.12	1,803,643	3,304	300,625	1,218	1,788,111	3,245	795,348	3,245	21,578	615	2,883,699
HT4.5	1,774,000	3,410	321,471	1,281	1,726,629	3,245	809,898	3,304	18,028	535	2,515,905
HT4.6	1,804,746	3,472	321,496	1,302	1,687,486	3,120	761,000	3,180	19,397	577	2,741,893
AR727.7	1,626,205	3,240	296,152	1,239	1,529,798	2,958	710,796	3,016	15,585	504	2,555,154
AR727.14	1,560,356	2,964	251,496	1,116	1,684,825	3,420	780,311	3,420	16,308	552	1,665,159
AR728.7	1,625,402	2,928	223,996	930	1,757,318	3,249	815,998	3,249	16,286	573	2,856,406
AR728.10	1,693,911	3,364	310,434	1,334	1,586,595	3,009	710,811	3,068	16,429	579	2,389,583
AB2.13	2,028,426	3,360	329,649	1,392	1,932,862	3,060	742,023	3,120	24,358	749	2,608,270
CR2.4	2,117,793	3,410	413,313	1,488	1,990,642	3,087	849,889	3,087	22,049	745	2,291,926
